# Biosynthesis of the *Paecilomyces marquandii* conidial pigment saintopin

**DOI:** 10.1186/s40694-025-00199-4

**Published:** 2025-06-05

**Authors:** Carsten Wieder, Sarah Galwas, Rainer Wiechert, Kevin Seipp, Alexander Yemelin, Eckhard Thines, Till Opatz, Anja Schüffler

**Affiliations:** 1 Institute of Molecular Physiology, Johannes Gutenberg-University, Hanns-Dieter-Hüsch Weg 17, D-55128 Mainz, Germany; 2 Institut für Biotechnologie und Wirkstoff-Forschung gGmbH, Mainz, Hanns-Dieter-Hüsch Weg 17, D-55128 Mainz, Germany; 3 Department of Chemistry, Johannes Gutenberg-University, Duesbergweg 10–14, D-55128 Mainz, Germany

**Keywords:** Natural products, Conidial pigment, Non-reducing polyketide synthase, Biosynthesis, Heterologous expression

## Abstract

*Paecilomyces marquandii* IBWF 003–21 produces vibrant purple pigmented conidia, the color of which can be attributed to the naphthacenedione natural product saintopin (**1**). The target compound was previously reported to exhibit potent topoisomerase-inhibitory activity, yet has not been extensively studied nor has the biosynthesis been elucidated. In an effort to elucidate the biosynthesis of **1**, we mined the genome of *Paecilomyces marquandii* for non-reducing polyketide synthases (nrPKS), introduced them into the heterologous host *Aspergillus oryzae* OP12 and identified a prime candidate for the biosynthesis of **1** we termed *stpA*. Deletion of *stpA* in the native producer *P. marquandii* abolished production of **1**, rendering conidia hyaline in color. *stpA* phylogenetically clusters with clade V nrPKS, canonically requiring trans-acting metallo-β-lactamase-like thioesterases (MβL) for product offloading, however, no MβL is encoded in the vicinity of *stpA*. Instead, a BLAST-search revealed a single MβL, *stpB*, encoded elsewhere in the *P. marquandii* genome, accompanied by a flavin-dependent monooxygenase (FMO), *stpC*, and an *O*-methyltransferase, *stpD*. Heterologous coexpression of *stpA* and *stpC* sufficed for reconstituting **1** biosynthesis in *A. oryzae* OP12 even without additional coexpression of *stpB*. Coexpression of *stpC* alongside the decaketide-synthase *adaA* involved in TAN-1612 biosynthesis also resulted in the production of **1**, which implies that the formation of **1** proceeds *via* a decaketide precursor that is subsequently shortened. While the structure and biosynthesis of **1** are unique compared to other fungal naphthacenediones, further research is necessary to elucidate the elusive mechanism underlying the formation of **1**.

**Supplementary Information:**

The online version contains supplementary material available at 10.1186/s40694-025-00199-4.

## Graphical abstract

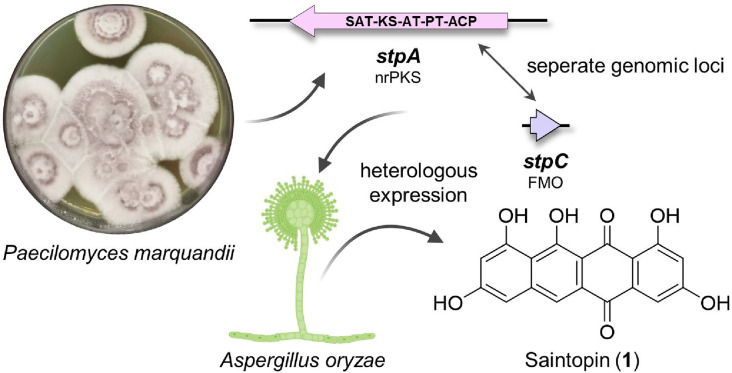

## Introduction

Fungal polyketides have been extensively studied for their structural diversity and promising biological activities exploitable for medicinal and agricultural applications. Notable examples include the blockbuster cholesterol-lowering drug lovastatin [1], the antifungal agent strobilurin A produced by *Strobilurus tenacellus* that was used as the lead structure for the development of β-methoxyacrylate agri-fungicides [2, 3] as well as the fungistatic drug griseofulvin commonly used for treatment of onychomycosis [4, 5]. Most fungal polyketides are synthesized by type I iterative polyketide synthases (PKS), multidomain enzymes that catalyze repeated decarboxylative Claisen condensation to produce polyketides of varying modifications and chain length [6–8].

PKS are divided into reducing and non-reducing types based on their inherent set of domains. All PKS share essential ketosynthase (KS), acyltransferase (AT) and acyl-carrier protein (ACP) domains and can include additional optional *C*-methyltransferase (CMeT), reductase (R) or thioesterase (TE) domains. Non-reducing PKS additionally canonically harbor a starter acyltransferase (SAT) [9] and product template (PT) [10] domain responsible for initial substrate loading and product cyclization, respectively, but lack reductive domains found in reducing PKS. The function of the SAT domain is essential in some cases, particularly when loading unusual starter units such as hexanoyl-CoA [9] or in hr/nrPKS hybrid pathways [11], but has been shown to be dispensable without compromising function in recently described basidiomycete nrPKS [12]. The product template domain has been suggested to act as a substrate tunnel that prefolds the nascent polyketide to facilitate aldol-condensation mediated cyclization [13], therefore being responsible for the (poly-)cyclic structure of non-reduced polyketide products.

**Fig. 1:**
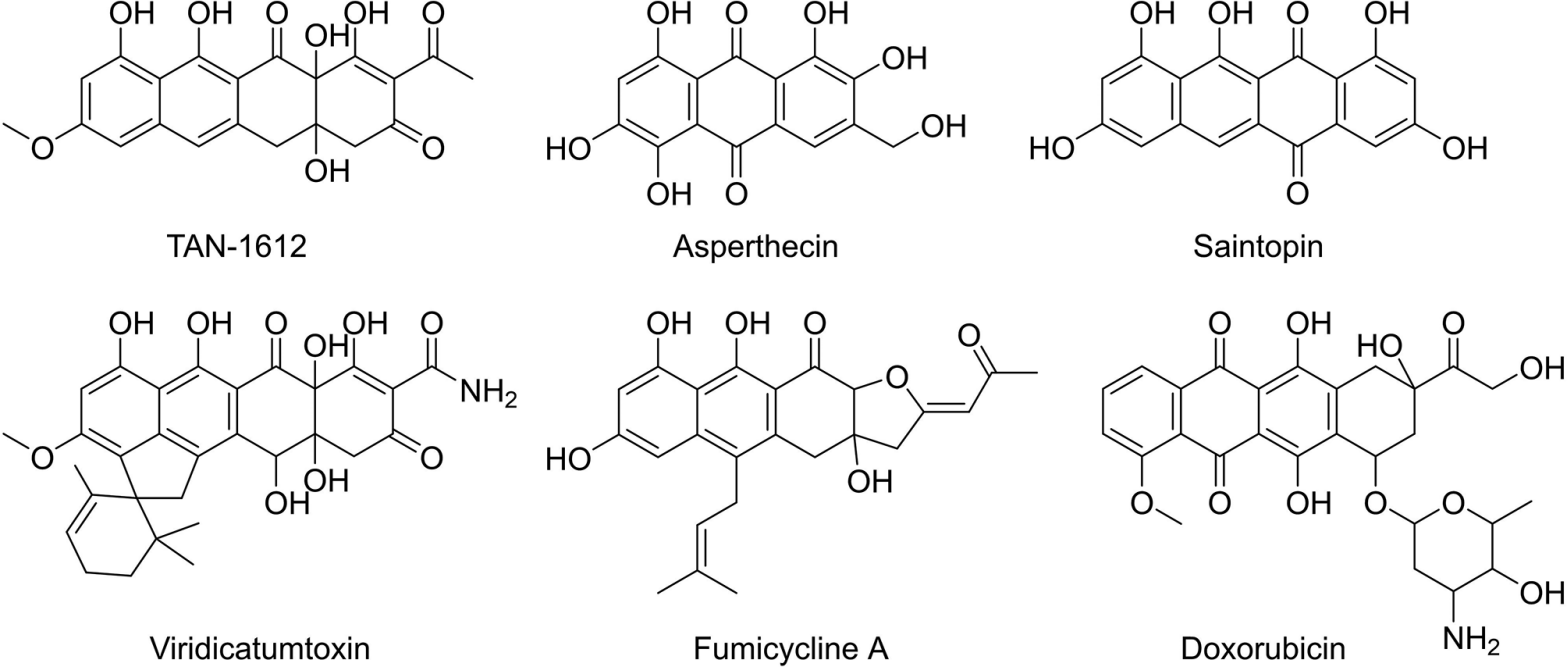
Selected structures of fungal naphthacenediones and related polyketides

Phylogenetically, nrPKS cluster into 12 distinct clades, each clade sharing characteristic features in domain architecture, polyketide length and cyclization pattern [12–15]. In contrast to most other nrPKS, clade V nrPKS lack a terminal R- or TE-domain for product offloading, the function of which is commonly complemented by a metallo-β-lactamase-like TE (MβL) protein encoded in the genomic vicinity of clade V nrPKS, firstly described in atrochrysone carboxylic acid biosynthesis [16]. Furthermore, clade V has recently been subdivided into three subclades (V-I, V-II and V-III) [12, 15]. While clade V-I and clade V-III PKS produce octaketides such as atrochrysone/endocrocin [16] and unusual heptaketides such as alternariol [17] and the benzophenone precursor of griseofulvin [18], clade V-II PKS produce nona- and decacetides such as asperthecin [19, 20], TAN-1612 [20], viridicatumtoxin [21], fumicycline [22], neosartoricin [23] and hancockinone A [24] (Fig. 1). Besides MβL, clade V-II nrPKS additionally cluster with flavin-dependent monooxygenases (FMOs). These FMOs catalyze C2-hydroxylation, which is indispensable for cyclization of the fourth ring in the case of naphthacenediones like TAN-1612, as their absence alternatively results in hydrolytic release of a tricyclic product [20].

The fungal naphthacenedione saintopin (Fig. 1), isolated for the first time in 1990 from *Paecilomyces* sp., has previously been shown to exhibit potent topoisomerase I & II inhibitory activity, making it a promising candidate for development as an anti-tumor agent [25]. Indeed, the structurally related bacterial anthracyclines such as doxorubicin (marketed as Adriamycin^®^, Fig. 1) are already being used to treat various cancers, albeit at the high risk of cardiotoxicity [26]. Despite the promising activity originally reported, only few studies on saintopin have been conducted since. While saintopin is structurally related to other fungal naphthacenediones it does harbor unique structural features, such as the lack of side chains and the resulting planarity (Fig. 1), implying an alternative or extended biosynthetic route. Here we report the isolation of saintopin from *Paecilomyes marquandii*, which is responsible for the conidial pigmentation in the producing organism, as well as identification and heterologous reconstitution of saintopin biosynthetic genes in *Aspergillus oryzae*. Saintopin biosynthesis only requires two enzymes and features an elusive mechanism of polyketide cyclization diverging from other characterized fungal naphthacenedione pathways.

## Results

### Isolation of the *Paecilomyces marquandii* conidial pigment saintopin

**Fig. 2:**
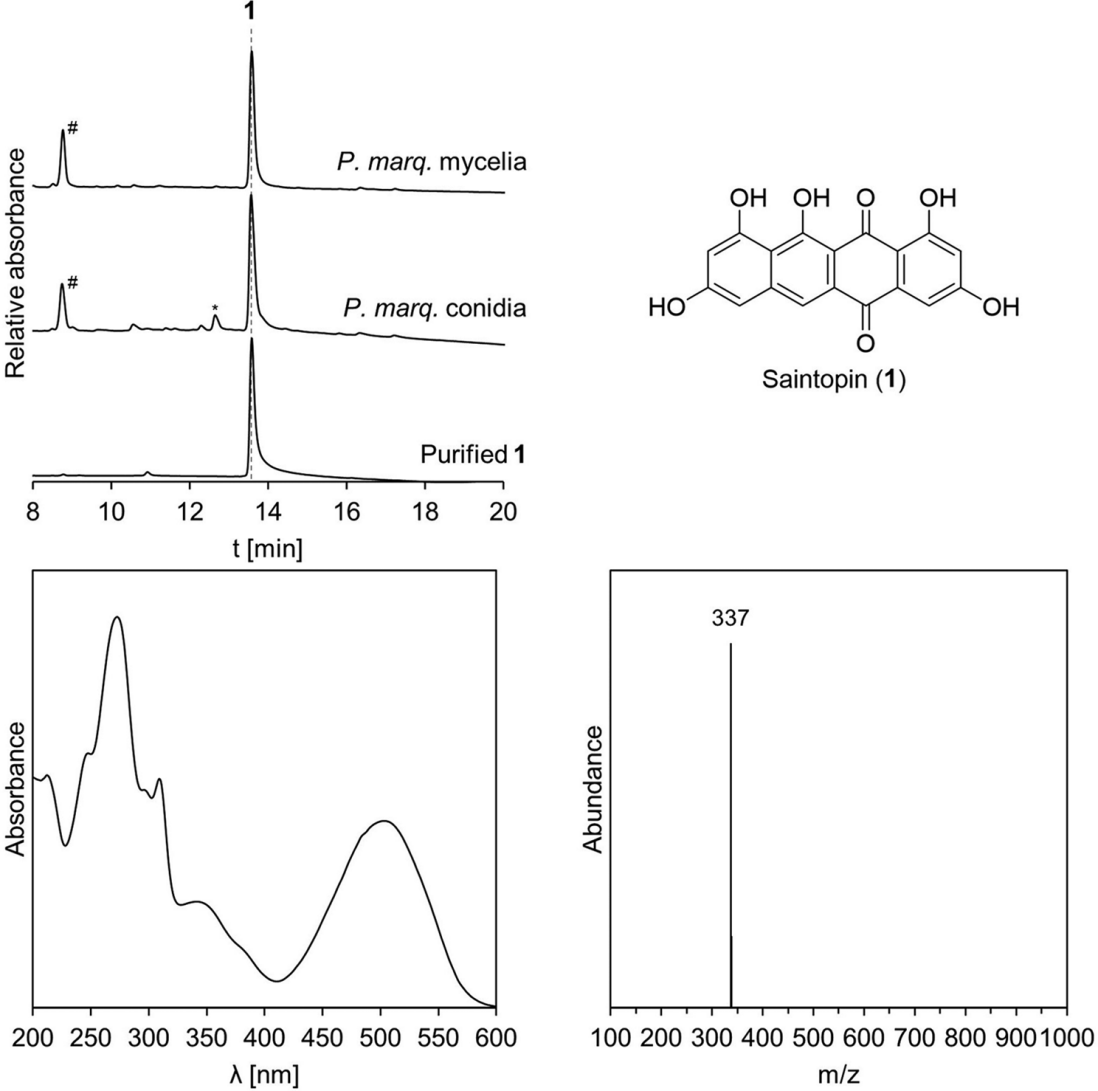
Saintopin (**1**) is produced in *Paecilomyces marquandii* conidia and mycelia. Chromatograms (250 nm) of *P. marquandii* conidia extract, mycelia extract and purified **1**. Structure, UV/Vis- and neg. ionization mass spectrum of **1**. * corresponds to compound **10**. # unrelated (non-purple colored) compound

A fungus with vibrant purple conidial pigmentation was isolated from a contaminated agar plate in Mainz, Germany. Sequencing of the barcode ITS region (Table S4) identified the isolate as *Paecilomyces marquandii* (synonym: *Marquandomyces marquandii*) and the strain was deposited at the Institut für Biotechnologie und Wirkstoff-Forschung gGmbH (IBWF, Mainz, Germany) with reference number IBWF 003–21. The major metabolite produced in conidia of *P. marquandii* was identified to be compound **1**, the UV/Vis spectrum of which did align with the purple color of the conidia (Fig. 2). Fortunately, compound **1** was also produced in liquid culture and preparative amounts were obtained from the extraction of the mycelia of *P. marquandii* from a 10 L scale-up fermentation. The purple pigment was isolated from the crude extract using chromatographic techniques and the metabolite identified as saintopin (**1**) by NMR spectroscopy and HRESIMS analysis (Fig. 2).

### Heterologous expression of nrPKS genes identifies a prime candidate for saintopin biosynthesis

To identify the biosynthetic origin of saintopin (**1**), the genome of *P. marquandii* IBWF 003–21 was sequenced and assembled into 42.3 Mb, distributed across 406 contigs, with an N50 of 0.7 Mb and a GC content of 51.6%. The length of the largest contig was 2.6 Mb. The results indicated a high-quality genome assembly. A total of 6,617,776 clean reads were obtained from Illumina sequencing, enabling the prediction of 12,761 protein-coding genes, 8,987 of which had functional annotations. AntiSMASH analysis supplemented by manual curation using FunBGCeX results revealed the presence of 81 secondary metabolite (SM) biosynthetic gene clusters (BGCs), comprising 23 nonribosomal peptide synthetases (NRPSs), 16 polyketide synthases (PKSs), 14 terpene synthases (TPSs), 5 hybrid and 23 other BGCs (e.g., ribosomally synthesized and post-translationally-modified peptides (RiPPs), isocyanide synthases (ICS)).

Given the aromatic structure of saintopin (**1**), we hypothesized that its biosynthesis is mediated by a nrPKS. Compound **1** shares structural similarity with other fungal naphthacenediones, such as TAN-1612 and viridicatumtoxin (Fig. 1), however differs in functionalization and substitution, suggesting a different biosynthetic pathway. Among the PKSs identified, seven were categorized as non-reducing. One of these was excluded from further analysis due to high similarity with the sorbicillin BGC of *Acremonium chrysogenum*. The six remaining nrPKS genes were cloned into expression plasmids and introduced into the heterologous host *Aspergillus oryzae* OP12 *pyrG*^–^ [27] to investigate product formation. This strain features a coupled promoter system, which amplifies gene expression and therefore results in higher SM production.

**Fig. 3:**
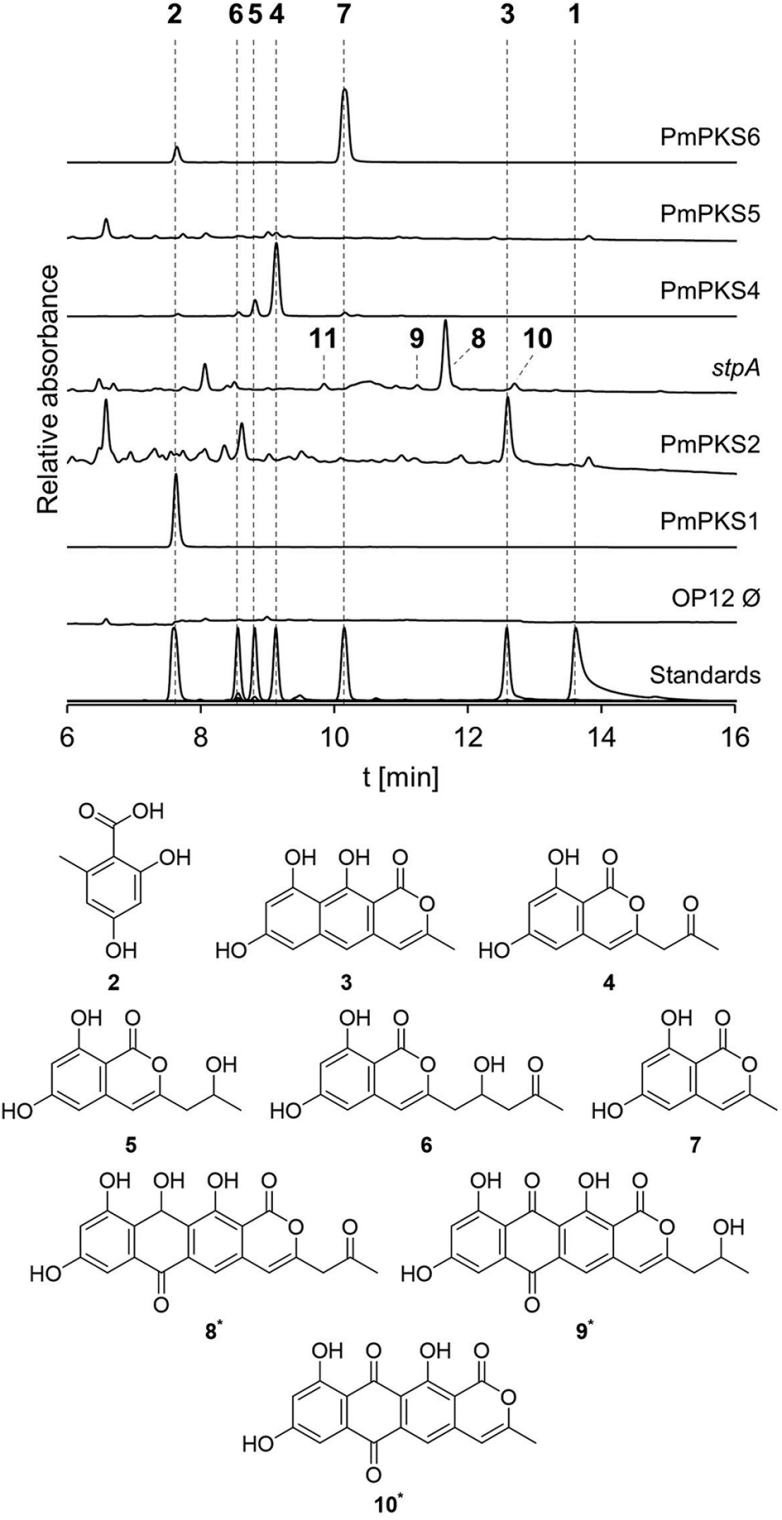
Heterologous expression of *P. marquandii* nrPKS genes in *A. oryzae* OP12 results in production of different chain length aromatic polyketides. Chromatograms (250 nm) of mutant culture filtrate extracts and purified metabolites. Structures of **2**–**7** were elucidated by NMR and HRESIMS. * Structures of **8**–**10** are proposed. Ø, empty plasmid control strain

Five out of the six nrPKSs produced detectable products in the heterologous host, as evident from the comparison with the empty plasmid control strain (Fig. 3). Most products were purified from scale-up fermentation of the mutant *A. oryzae* strains and their structures were elucidated by NMR and HRESIMS analysis. While PmPKS1 and PmPKS2 are strictly orsellinic acid (**2**) and nor-toralactone (**3**) synthases, respectively, PmPKS4 and PmPKS6 produced multiple products of different chain lengths. OP12_PmPKS4 predominantly produced 6,8-dihydroxy-3-(2-oxopropyl)-1*H*-2-benzopyran-1-one (**4**), orthosporin (**5**), minor amounts of citreoisocoumarin (**6**) and saccharonol A (**7**). OP12_PmPKS6 produced compounds **2** and **7**. No products were produced by OP12_PmPKS5.

OP12_*stpA* produced only traces of several compounds, the major one being **8** (379 Da [M–H^+^]) and even smaller amounts of **9** (381 Da [M–H^+^]), **10** (337 Da [M–H^+^]) and **11** (355 Da [M–H^+^]) (Fig. 3, Figure S2). Unfortunately, we were not able to obtain sufficient amounts of the compounds produced by OP12_*stpA* for structure elucidation, as yields were too low and production was not scalable. Compounds **8**, **9** and **10** shared the same UV/Vis spectra, suggesting similar molecular structures (Figure S1). The UV/Vis and identified masses of **8** and **10** align with compounds that were previously identified when the clade V nrPKS AdaA was assayed in vitro [20]. Based on this congruence, we propose the structures of **8** and **10** as the previously reported deca- and nonaketide anthrapyrones (Fig. 3). As compound **9** also shares the same UV/Vis and differs from **8** by only + 2 Da, we propose **9** to be the reduced congener of **8**. The structure of compound **11** remains ambiguous. Interestingly, upon reinvestigation of *P. marquandii* extracts for compounds **2**–**11**, compound **10** could also be detected in the conidia extract of *P. marquandii* (Fig. 2). In conclusion, *stpA* seems to be the only nrPKS encoded in the genome of *P. marquandii* producing polyketides of sufficient chain length required for **1** production, making it the prime candidate for the biosynthesis of **1**.

### Deletion of *stpA* abolishes saintopin production in *P. marquandii*

**Fig. 4:**
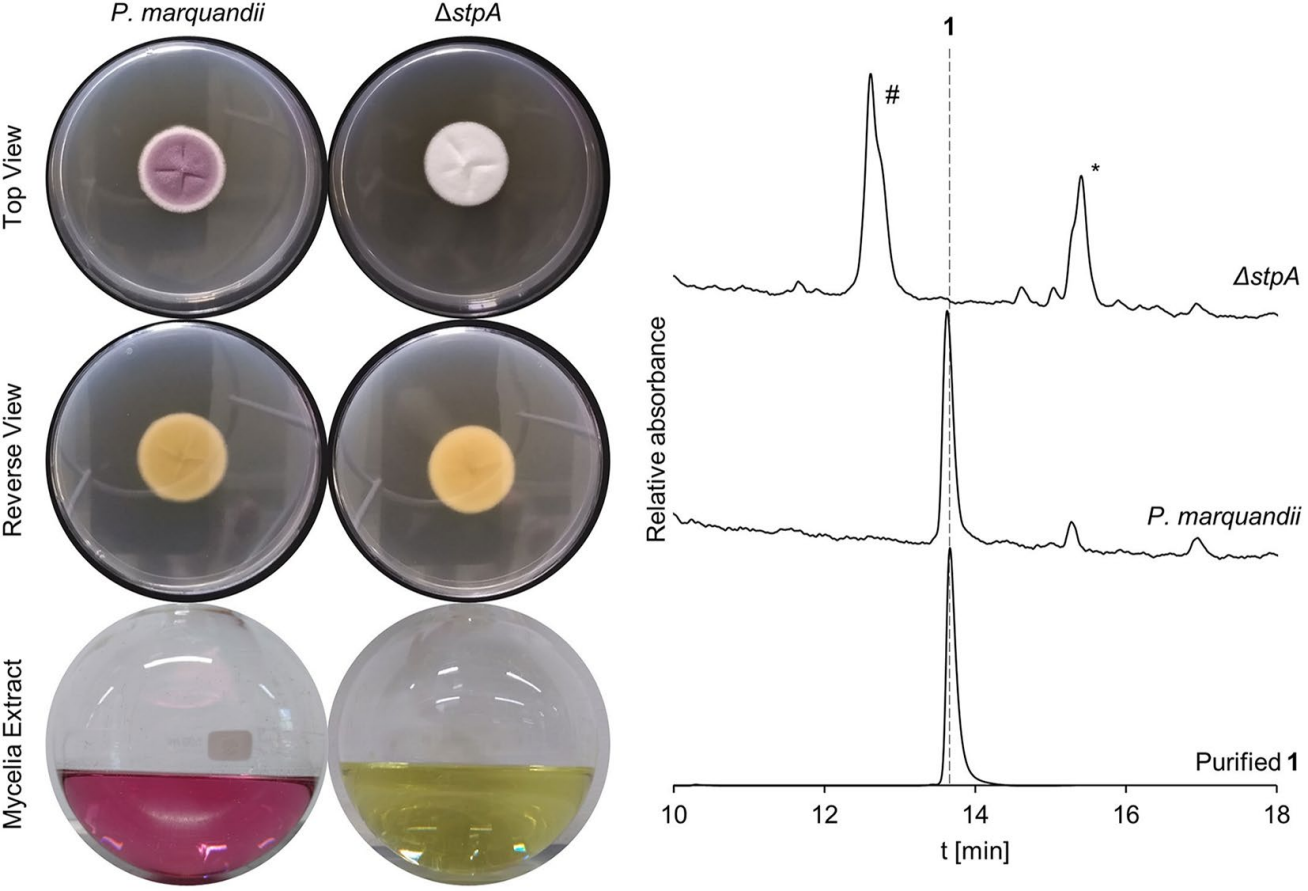
Deletion of *stpA* results in the loss of conidial pigmentation and abolishment of **1** production. Morphology of *P. marquandii* and *ΔstpA* cultures with their respective mycelia extracts and chromatograms (250 nm). * Presumably trichodimerol by comparison to an internal standard. # Unidentified compound

To assess the involvement of *stpA* in saintopin (**1**) biosynthesis, we first sought to establish a transformation protocol for *P. marquandii*. Fortunately, employing previously established protocols frequently used for protoplasting aspergilli did also successfully yield protoplasts from *P. marquandii* mycelia, albeit after prolonged incubation. However, *P. marquandii* seems highly insensitive to the most commonly used selection agents (as apparent by pre-tests, data not shown, e.g., hygromycin, phleomycin, BASTA, nourseothricin). Despite background growth of untransformed colonies on the transformation plates we eventually succeeded in obtaining a single *ΔstpA* mutant by using geneticin (G418) as a selection agent at 1000 µg/mL (Figure S3). *ΔstpA* exhibits an albino phenotype, lacking conidial pigmentation, which is due to the abolishment of the production of **1** (Fig. 4).

### Heterologous reconstitution of saintopin biosynthesis

**Fig. 5:**
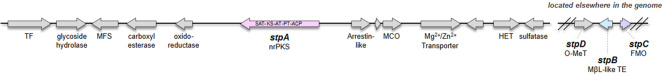
Scheme of the *stp* BGC

With *stpA* identified as the core gene involved in the biosynthesis of **1**, we next sought to reconstitute additional accessory genes in *A. oryzae* to elucidate the biosynthetic pathway. StpA has a SAT-KS-AT-PT-ACP domain architecture and phylogenetically clusters with clade V nrPKS, which lack an intrinsic offloading domain and therefore require trans-acting MβL for product offloading. More specifically, StpA belongs to clade V-II enzymes such as AdaA and VrtA, that are involved in the biosynthesis of the fungal naphthacenediones TAN-1612 and viridicatumtoxin, and the respective biosynthetic pathways require action of an FMO for cyclization of the fourth ring. However, neither a MβL nor a FMO are encoded in the vicinity of *stpA*. A BLAST search of the MβL-coding gene *adaB* identified a single homolog in the genome of *P. marquandii* we termed *stpB*. Interestingly, homologs of the FMO *adaC* and the *O*-methyltransferase *adaD* are encoded next to *stpB*, which we consequently termed *stpC* and *stpD*, respectively (Fig. 5; Table 1).

**Table 1:** Proposed function of *stp* genes

Gene	Size (aa)	BlastP hit*	Identity (%)	E-value	Proposed protein function
*stpA*	1824	D7PHZ2.1	69.01	0.0	nrPKS (SAT-KS-AT-PT-ACP)
*stpB*	319	Q4WA58.1	60.00	1e-141	metallo-β-lactamase-like thioesterase
*stpC*	421	D7PHZ9.1	59.40	0.0	FAD-dependent monooxygenase
*stpD*	244	D7PHZ7.1	60.99	3e-100	*O*-methyltransferase

*Uniprot as reference database

We next performed sqRT-PCR analysis (Figure S4) of all genes surrounding *stpA* and the trans-cluster encoding *stpBCD* with cDNA derived from cultures producing or not producing **1**. Unfortunately, the results did not show any correlation between gene expression and production of **1**, implying an alternative regulatory mechanism, making it difficult to assess genes involved in the biosynthesis of **1**. To experimentally probe accessory genes involved in the biosynthesis of **1**, we co-introduced the homologs necessary for the production of naphthacenediones in other biosynthetic pathways, namely *stpABC*, into the triple auxotrophic *A. oryzae* OP12 3Δ [28] and performed metabolite analysis as previously.

**Fig. 6:**
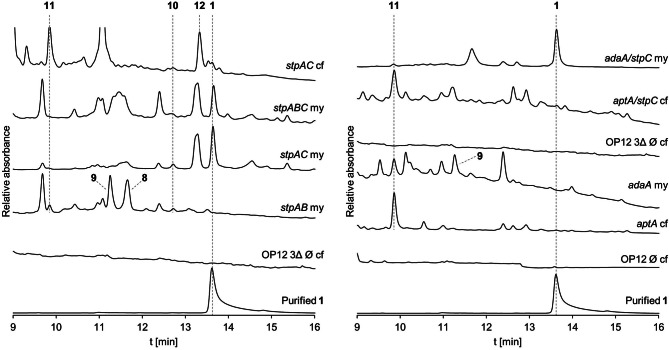
Heterologous coexpression of *stpAC* suffices to reconstitute saintopin (**1**) biosynthesis in *A. oryzae*. Chromatograms (250 nm) of mutant mycelia and culture filtrate extracts and purified **1**. my, mycelia extract. cf, culture filtrate extract

Coexpression of *stpA* and *stpB* did not yield any new compounds, other than those already produced by solely *stpA*, namely trace amounts of **8**, **9**, **10** and **11** (Fig. 6). Intriguingly, the coexpression of *stpABC* but also *stpAC* resulted in the production of **1** (Fig. 6) and various other, presumably shunt products (**13**–**18**, Figure S5). Compounds **10** and **11** were still produced in OP12_*stpAC* and OP12_*stpABC*, whereas compounds **8** and **9** could not be detected. An additional compound **12** (323 Da [M–H^+^]) was detected predominantly in culture filtrate extracts of OP12_*stpAC* and OP12_*stpABC*. Attempts towards purifying **12** were unsuccessful, since all fractions purified by preparative HPLC additionally contained **1**. The mass difference of 14 Da between **12** and **1** might indicate spontaneous oxidation of a non-quinone precursor we termed presaintopin (**12**) in the presence of oxygen to afford the quinone moiety in saintopin (**1**). This would explain the presence of **1** in purified fractions of **12**. During preparation of mycelia extracts, compounds are exposed to atmospheric oxygen for a longer period of time as compared to culture filtrate extracts, resulting in complete oxidation of **12** to **1**, and non-detection of **12** in mycelia extracts. Indeed, during the preparation of mycelia extracts of *P. marquandii*, OP12_*stpAC* or OP12_*stpABC*, the extracts are initially orange-colored and will only turn increasingly purple with prolonged incubation, indicative of conversion of **12** to **1**.

**Fig. 7:**
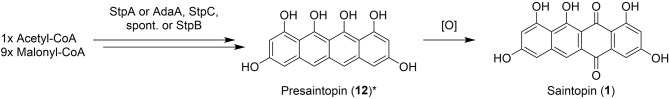
Proposed biosynthesis of saintopin (**1**). * Structure of **12** is proposed

The mechanism underlying the formation of saintopin (**1**) is elusive as the reaction catalyzed by StpC is not readily apparent from the structure of **1**. To gain first mechanistic insights into the formation of **1**, *stpC* was introduced alongside either the decaketide synthase *adaA* or the nonaketide synthase *aptA* into *A. oryzae* OP12 3Δ (Fig. 6). Expression of *aptA* and *aptA*/*stpC* resulted in the production of only **11**, with no apparent differences between the two mutants, whereas formation of **1** was not observed (Fig. 6). In contrast, expression of *adaA* and *adaA*/*stpC* resulted in the production of the same compounds observed for *stpA* and *stpAC*, respectively, with *adaA*/*stpC* producing **1** (Fig. 6, Figure S6). This implies the necessity of a decaketide precursor for the production of **1**. Based on these findings we propose a biosynthetic scheme for the production of **1** (Fig. 7).

## Discussion

In the present study, we successfully linked the purple color of *P. marquandii* conidia to the production of the naphthacenedione polyketide pigment saintopin (**1**). While most fungi produce pigmented conidia, the pigments differ between species. Many fungi produce melanins for conidial pigmentation, oligo-/polymers derived through oxidative polymerization from various monomeric precursors, such as dihydroxynaphthalene (DHN), L-dioxyphenylalanine (DOPA) or the more recently described aspulvinone E [29, 30]. Amongst others, melanins have been associated with photoprotection, they have shown antioxidant properties, they contribute to temperature- and osmo-tolerance and they are vital for cell development and virulence in pathogenic fungi [29, 31, 32]. Irrespective of the type of pigment produced, conidial pigmentation plays a vital role in protection against UV-induced DNA damage [33]. It was previously shown for another *Paecilomyces* species that production of the conidial pigment YWA1 increases tolerance towards UV irradiation [34]. Furthermore, bis-naphthopyrone pigments produced by *Fusarium* species display non-toxic antifeedant activity against fungivorous animal predators [35]. While the exact biological and ecological function of **1** in *P. marquandii* remains unclear, it likely plays a similar protective role described for other conidial pigments.

To elucidate the biosynthesis of saintopin (**1**), we first performed heterologous expression of nearly all nrPKS encoded in the genome of *P. marquandii* in the heterologous host *A. oryzae* OP12 [27, 28]. Whereas five of six nrPKS produced different polyketide products of varying chain lengths (**2**–**11**), expression of PmPKS5 did not lead to the production of a polyketide product. This might be due to the involvement in a hybrid/cooperative biosynthetic pathway, where the nrPKS is primed by e.g., hrPKS- or FAS-derived precursors [11, 36]. Production of polyketides of different chain lengths in varying abundancies from a single nrPKS, as is the case for PmPKS4 and PmPKS6, is frequently observed [37–39]. The reduced polyketides **5**, **6** and **9** are shunt products that likely derive from the action of an endogenous, promiscuous reductive enzyme of *A. oryzae* such as e.g., AoiI, involved in the reduction of **4** to **5** in the *A. oryzae* 8-methyldiaporthin biosynthetic pathway [40]. Moreover, compounds **8**–**10** are proposedly quinones that derived from their respective non-quinone precursors through spontaneous oxidation in the presence of oxygen, similar to the proposed conversion of **12** to **1**. Spontaneous quinone formation has previously been observed for other aromatic polyketides such as e.g., endocrocin [16] and flaviolin [41].

Heterologous expression of PmPKS2, PmPKS3, PmPKS4 and PmPKS6 yielded large amounts of their respective polyketide products, more than sufficient for isolation and structure elucidation. In contrast, heterologous expression of *stpA* only led to production of trace amounts of the nona-/decaketides **8**–**11**. Since clade V nrPKS do not harbor an intrinsic offloading domain, product release from StpA likely only occurs *via* slow spontaneous hydrolysis or α-pyrone formation in the absence of a trans-acting offloading enzyme. The low yield of **8**–**11** hindered the production of sufficient amounts for isolation and structure elucidation. While the structures of **8**–**10** were proposed based on previously reported products produced by AdaA in vitro [20], the structure of **11** remains ambiguous. Nevertheless, **11** is assumed to be a nonaketide, as it was also produced by OP12 expressing *aptA*, which reportedly is a nonaketide synthase [20]. As the compounds produced by OP12_*stpA* are the only polyketides sufficiently similar in size to **1**, we deleted *stpA* in the native producer. This resulted in the loss of conidial pigmentation and abolished the production of **1**, which unequivocally linked the biosynthesis of saintopin (**1**) to the nrPKS *stpA*.

As *stpA* does not suffice for **1** production, we aimed to investigate the accessory genes required for biosynthesis. Homologs of the MβL *adaB*, FMO *adaC* and *O*-methyltransferase *adaD* involved in TAN-1612 biosynthesis [20] were found located elsewhere in the genome of *P. marquandii* instead of clustered alongside *stpA*. While encountered only infrequently in fungi, there are reports on BGCs that are split across multiple loci in the genome, such as e.g., the austinol biosynthetic genes in *Aspergillus nidulans* [42]. As no correlation between **1** production and expression of *stpABCD* and any of the genes encoded in the vicinity of *stpA* was observed in sqRT-PCR analysis, *stpBC* were coexpressed alongside *stpA* in an effort to identify biosynthetic intermediates. Unexpectedly, coexpression of *stpAC* did already suffice to reconstitute the production of **1** in *A. oryzae*, whereas additional coexpression of *stpB* did not result in any apparent differences. Apart from **1**, OP12_*stpAC* and OP12_*stpABC* produced various, presumedly shunt products (**13**–**18**) that might arise from **12** or **1** *via* the action of endogenous *A. oryzae* enzymes, which might serve as a strategy to cope with the reported toxicity of **1** [25]. The biosynthesis of **1** is proposed to proceed *via* the non-quinone intermediate **12** that spontaneously undergoes quinone-oxidation in the presence of oxygen.

To gain first insights into the rationale underlying the formation of **1**, the nona- and decaketide synthases *aptA* and *adaA* were heterologously expressed in *A. oryzae* OP12 with and without additional coexpression of *stpC*. Expression of *aptA* and *aptA/stpC* resulted in the production of only **11**, which was contrary to our expectations, as AptA was previously reported to produce **10** in vitro [20]. Heterologous expression of *adaA* resulted in the production of the same nona- and decaketide products that were also produced by *stpA*, and coexpression of *adaA/stpC* similarly resulted in the production of the same compounds produced by *stpAC* and *stpABC*, including **1**. This implies the formation of **1** to proceed *via* a decaketide (C_20_) precursor, although the structure of **1** contrary suggests a nonaketide (C_18_) origin. Therefore, the decaketide precursor seems to be deactetylated during the StpC-mediated formation of **1**.

**Fig. 8:**
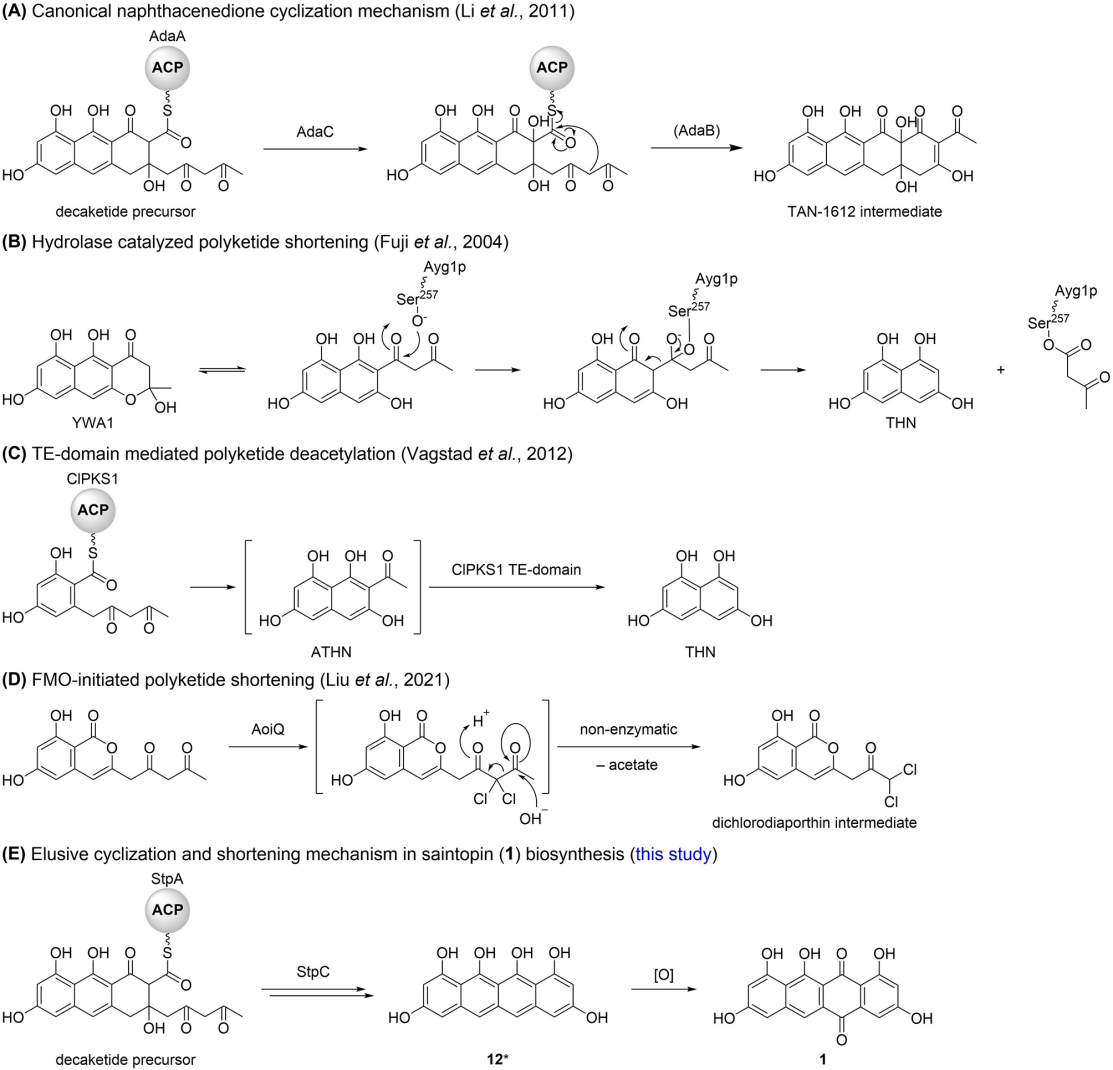
Cyclization and polyketide shortening mechanisms in polyketide biosynthetic pathways [20, 45, 47, 50]

Intriguingly, **1** does not contain a methyl- (e.g., atrochrysone carboxylic acid), acetyl- (e.g., ATHN) or longer chain poly-β-ketone (e.g., YWA1) side chain usually present in non-reduced polyketide scaffolds that arises from the mechanism of terminal cyclization. The only other fungal-derived polyketide sharing this structural feature is the melanin precursor THN. It is usually synthesized from the precursors ATHN [43] or YWA1 [44–46] through enzymatic polyketide shortening (deacetylation or deacetoacetylation) by hydrolases such as Ayg1p [45] (Fig. 8B). However one enzyme, ClPKS1, was described to synthesize THN directly. It was previously assumed that THN is synthesized by ClPKS1 from solely malonyl-CoA [41], facilitating the late decarboxylation of the starter malonate-unit and therefore elimination of the sidechain during second ring cyclization, this hypothesis was however later proven incorrect. Even though ClPKS1 is indeed a true THN synthase, it was shown that ATHN emerges as a bound intermediate, which however is deacetylated by ClPKS1 unique TE domain during Dieckmann cyclization [47] (Fig. 8C). While it was recently shown that basidiomycete nrPKS are capable of synthesizing polyketides solely from malonyl-CoA [12], no enzyme has been reported to date that catalyzes decarboxylation-facilitated terminal cyclization as initially proposed for ClPKS1 [41]. Recently, another intriguing polyketide shortening mechanism has been reported to occur in dichlodiaporthin biosynthesis [50]. The flavin-dependent halogenase (FDH) domain of the bifunctional enzyme AoiQ *gem*-dichlorinates the 1,3-diketone moiety of a polyketide substrate, resulting in deacetylation facilitated by spontaneous nucleophilic addition of water at the terminal carbonyl moiety and subsequent cleavage [50] (Fig. 8D).

The fungal naphthacenediones reported so far, namely TAN-1612, viridicatumtoxin [21], hypomycetin [48] and anthrotainin [49], all share the same basic scaffold implying a common biosynthetic rationale. While viridicatumtoxin biosynthesis has only been proposed based on the deletion of a few biosynthetic enzymes [21], TAN-1612 biosynthesis has been investigated in detail [20], offering insights into the canonical mechanism for fungal naphthacenedione formation. The nrPKS involved in TAN-1612 biosynthesis, AdaA, belongs to clade V-II nrPKSs. Therefore, AdaA lacks a terminal offloading domain and is dependent on the trans-acting MβL AdaB for product release [16]. First and second ring cyclization in TAN-1612 biosynthesis are catalyzed *via* PT domain mediated aldol condensation whereas the third ring cyclization occurs *via* spontaneous aldol cycloaddition, resulting in the conservation of a hydroxyl moiety at position C15. The cyclization of the fourth ring is in turn catalyzed by the MβL AdaB but requires C2-hydroxylation introduced by the FMO AdaC (Fig. 8A). The other related fungal naphthacenediones also share this structural feature of C2- and C15- hydroxylation, whereas **1** lacks both hydroxylations. While AdaB is dispensable for TAN-1612 formation, as fourth ring cyclization can also occur spontaneously after C2-hydroxylation, coexpression did substantially improve productivity in the heterologous host *S. cerevisiae* [20]. In contrast, no apparent differences in productivity were observed between *stpABC* and *stpAC*. Although cross-complementation of AdaA with AptB was reported to be unsuccessful [20], it cannot be ruled out that StpA could potentially be cross-complemented by the *A. oryzae* intrinsic (solely encoded) MβL DiaB (AO090701000529), canonically involved in dichlorodiaporthin biosynthesis [50]. Alternatively, StpB might be entirely dispensable for the production of **1**.

The two related fungal decaketides fumicycline and neosatoricin are proposed to be released prior to FMO-catalyzed C2-hydroxylation [22, 23], resulting in the formation of non-naphthacenedione products, which suggests a diverging interaction between the FMOs and nrPKSs (on-line vs. post-synthesis modification). As saintopin (**1**) lacks C2-hydroxylation but formation of **1** is dependent on StpC, the function of StpC might be entirely different from other reported naphthacenedione FMOs. StpC seems to somehow facilitate both polyketide shortening and cyclization of the fourth ring in **1** biosynthesis. Although compound **8** is only produced in traces when only *stpA* is expressed, it cannot yet be ruled out, that oxidative transformation of **8** to **1** (or the non-quinone precursor of **8** to **12**) occurs post-synthesis. While heterologous reconstitution of saintopin (**1**) biosynthesis provides first evidence for a unique biosynthetic pathway (Fig. 8E), further research will be necessary in the future to elucidate the function of StpC and the elusive mechanism underlying saintopin (**1**) formation.

In summary, we identified the conidial pigment produced by *P. marquandii* as saintopin (**1**) *via* purification and structure elucidation. In an effort to unravel the biosynthesis of the unusual naphthacenedione **1**, we performed genome sequencing, genome mining and heterologous expression of nrPKS genes in *A. oryzae*, identifying a candidate nona-/decaketide nrPKS, *stpA*, involved in the biosynthesis of **1**. Establishing the transformation of *P. marquandii* enabled the deletion of *stpA* in the native producer, which led to the loss of conidial pigmentation and the abolishment of the production of **1**, unequivocally linking the biosynthesis of **1** to *stpA*. As no correlation was found between gene expression and the production of **1**, we reverted to heterologous coexpression of candidate accessory genes in *A. oryzae* to identify additional genes involved in the biosynthesis of **1**. Intriguingly, the coexpression of solely *stpA* and the FMO *stpC* led to the production of **1** in the heterologous host. As coexpression of the decaketide synthase *adaA*, but not the nonaketide synthase *aptA*, alongside *stpC* also led to the production of **1**, formation of **1** is proposed to proceed *via* a decaketide intermediate that is subsequently shortened. Although StpC seems to facilitate polyketide shortening and fourth ring cyclization in the biosynthesis of **1**, the underlying mechanism remains elusive as of yet.

## Methods

### Strains & cultivation

*Paecilomyces marquandii* strain IBWF 003–21 was isolated from a contaminated agar plate in Mainz, Germany in 2020 and is deposited at the Institut für Biotechnologie und Wirkstoff-Forschung gGmbH (IBWF, Mainz, Germany). *P. marquandii* was routinely cultivated on solid YMG (2% malt extract, 1% glucose, 0.4% yeast extract) agar plates at room temperature. *Aspergillus oryzae* OP12 *pyrG*^*–*^ and OP12 3Δ were routinely cultivated on GG10 (1% glucose, 10 mM glutamine, 0.152% KH_2_PO_4_, 0.052% KCl, 0.052% MgSO_4_∙ 7 H_2_O, 1 mL/L Hutners trace elements) supplemented with 10 mM uridine (OP12 *pyrG*^*–*^) or 10 mM uridine, 0.0001% PABA and 0.05% arginine (OP12 3Δ) at 30 °C. Spore suspensions of fungi were prepared in PBS supplemented with 0.01% Tween 80 and filtered through miracloth. *E. coli* strain NEB DH5α was used for plasmid propagation. All mutant strains used in this study are listed in Table S1.

### Bioinformatic analysis

The genomic DNA was sequenced using paired-end Illumina HiSeq 2500 with 2 × 150 bp reads. Initial quality assessment of the raw sequencing data was performed using FastQC (version 0.11.9) to ensure high data integrity. Following quality assessment, the reads were filtered for quality, and the assembly was carried out using SPAdes (version: 3.15.2). The filtered reads were assembled using SPAdes, with the following command:

/opt/anaconda3/envs/de_novo_assembly/bin/spades.py -1 BGC/1.fastq.gz -2 BGC/2.fastq.gz -o BGC/spades_kmers_set_careful_assembly1 -t 4 -k 77,99,121 --careful --only-assembler.

The SPAdes assembler was used with k-mer sizes of 77, 99, and 121, along with the *--careful* flag to minimize mismatches. The quality of the assembled contigs was evaluated using QUAST (version: 5.0.2), and the assembly with the highest N50 and largest contigs was selected for further analyses.

For gene prediction, the best assembly was processed using pretrained fungal models in Augustus, as implemented in Blast2GO. Functional annotation was performed by executing BLAST searches against the following databases: NR (NCBI non-redundant protein sequences), InterPro and Swiss-Prot.

IGV (version: 2.14.0) was used to visualize the assembled genome. The resulting GFF file, along with the scaffolds from the best assembly, was then utilized in antiSMASH (version: 7.1.0) analysis by leveraging specific profile hidden Markov models to identify fungal biosynthetic gene clusters (BGCs) encoded within the genome. The results were further compared and supplemented by FunBGCeX (Fungal Biosynthetic Gene Cluster eXtractor), a genome mining tool designed for fungal natural product discovery. FunBGCeX detects biosynthetic proteins by referencing a manually curated database of fungal natural product biosynthetic gene clusters (FunBGCs). Identified secondary metabolite (SM) BGCs were further investigated and characterized through manual curation of gene annotations using NCBI BLASTp analysis.

### Plasmid construction

Genomic DNA of fungi was prepared using the GeneJET Plant Genomic DNA Purification Kit (Thermo Fisher). PmPKS1, PmPKS2, *stpA*, PmPKS4, PmPKS5, PmPKS6, *stpB*, *stpC*, *aptA* and *adaA* were amplified from genomic DNA of *P. marquandii* IBWF 003–21, *Aspergillus nidulans* FGSC A4 or *Aspergillus niger* FGSC A1144 using the Q5-HotStart DNA-Polymerase (NEB) and assembled into *Nco*I restricted plasmids his_SM-Xpress_URA [27], SMX-Xpress_Ura [27], SM-Xpress_pabA [51] or SM-Xpress_argB(mut) [28] using the NEBuilder^®^ HiFi DNA Assembly 2 × Master Mix (NEB). For deletion of *stpA*, flanking regions (795 bp and 856 bp, respectively) were amplified from genomic DNA, the G418 resistance cassette was amplified from pC-G418-YR (Addgene plasmid No. 61767) [52] and assembled into *Sma*I-restricted pUC19 to make pKO_*stpA*_G418. The KO-cassette was retrieved from the plasmid using *Not*I prior to transformation. Expression plasmids were linearized prior to transformation using appropriate restriction enzymes. All oligonucleotides used for cloning are listed in Table S2.

### sqRT-PCR

RNA of *P. marquandii* was isolated using the RNeasy Plant Mini Kit (Qiagen). DNA was removed from RNA samples using DNaseI, RNAse-free (Thermo Fisher) prior to reverse transcription into cDNA using the RevertAid First Strand cDNA Synthesis Kit (Thermo Fisher). DreamTaq HotStart MM (Thermo Fisher) was used for all sqRT-PCRs according to the manufacturer’s instructions. cDNA concentrations were normalized to the signal intensity of the bTub amplicon. All sqRT-PCRs were carried out using the same reaction conditions. All oligonucleotides used for sqRT-PCR are listed in Table S3.

### Fungal transformation

Protoplast transformation of *A. oryzae* has been carried out as previously described [27]. *P. marquandii* was transformed analogously however took longer for protoplast formation (~ 6 h). Transformed *P. marquandii* protoplasts were regenerated on GG10S1.2 (GG10 supplemented with 1.2 M sorbitol) supplemented with 1000 µg/mL geneticin (G418) at room temperature for 10–14 days until single transformant colonies appeared.

### Fermentation, metabolite extraction, analysis & purification

After fermentation (see below), cultures were separated into mycelia and culture filtrate through vacuum filtration or filtering over miracloth. Culture filtrates were acidified with a few drops of 6 N HCl (improved solvent solubility of most aromatic polyketides), extracted with an equal amount of ethyl acetate and extracts were dried under vacuum at 45 °C. Mycelia and conidia were extracted with methanol: acetone (1:1) shaking at 120 rpm for one hour before filtration and drying under vacuum at 45 °C. Extracts were redissolved in MeOH and applied to HPLC-MS analysis using a LiChrospher 100 RP-18 (125 mm × 2 mm, 4 μm, Merck KGaA).

For secondary metabolite screening of OP12 mutants, spores were routinely inoculated into 50 mL 2% starch medium (2% soluble starch, 20 mM glutamine, 0.152% KH_2_PO_4_, 0.052% KCl, 0.052% MgSO_4_∙ 7H_2_O, 1 mL/L Hutners trace elements) and incubated shaking at 150 rpm and 30 °C for 2 days.

For isolation of RNA, *P. marquandii* was cultured in 500 mL of YMG, PDB (Difco) and CZ media (3% Sucrose, 0.3% NaNO_3_, 0.1% K_2_PO_4_, 0.05% MgSO_4_∙ 7 H_2_O, 0.05% KCl, 0.01% Fe_2_(SO_4_)_3_) shaking at 120 rpm at room temperature for 96 h. Samples of each culture were taken after 48 h, 72 h and 96 h, respectively, the mycelia was washed with H_2_O and snap-frozen in LN_2_. The mycelia was lyophilized and subsequently used for RNA extraction and metabolite extraction.

For comparison of metabolite production, *P. marquandii* WT and *ΔstpA* strain were cultured in 500 mL of YMG media for 4 days shaking at 120 rpm at room temperature.

For the isolation of **1**, *P. marquandii* was cultivated in 5 × 2 L YMG-medium shaking at 120 rpm for 6 days at room temperature. The mycelia was lyophilized and extracted with MeOH: acetone (1:1) overnight twice. The extract was dried *in vacuo* and pre-fractioned *via* SPE on a Bond Elut C18 column (Agilent) before subsequent purification using preparative HPLC.

For the isolation of **2** and **7**, the mycelia of an overnight 250 mL YEPD preculture of OP12 PmPKS6 was transferred into 2.5 L of 2% starch media and incubated shaking at 120 rpm at 28 °C for 3 days. For the isolation of compound **3** the mycelia of overnight 250 mL YEPD precultures of OP12 PmPKS2 was transferred into 2 × 2.5 L of 2% starch media and incubated shaking at 120 rpm at 28 °C for 3 days. For the isolation of compounds **4**–**6** an overnight 250 mL YEPD preculture of OP12 PmPKS4 was inoculated into 2.5 L of 2% starch media and incubated shaking at 120 rpm at 28 °C for 3 days. The culture filtrates were extracted with an equal amount of ethyl acetate and the crude extracts applied to preparative HPLC.

Compounds were purified by preparative HPLC on a Sunfire C18 column (100Å, 5 μm, 19 mm × 250 mm, Waters GmbH) running isocratic flows of ACN and 0.1% formic acid in H_2_O at 17 mL/min flow (eluent composition and yields are listed in Table 2). Crude extracts applied to preparative HPLC were dissolved in DMSO.

**Table 2:** Preparative HPLC eluent composition & compound yields

Compound	% ACN	Yield
Saintopin (**1**)	48	5.8 mg
Orsellinic acid (**2**)	37	42 mg
nor-Toralactone (**3**)	30	7.2 mg
6,8-dihydroxy-3-(2-oxopropyl)-1*H*-2-benzopyran-1-one (**4**)	28	104.7 mg
Orthosporin (**5**)	28	41.8 mg
Citreoisocoumarin (**6**)	28	1.8 mg
Saccharonol A (**7**)	37	71.1 mg

### Analytical chemistry and structure elucidation

#### Thin layer chromatography

Analytical thin-layer chromatography (TLC), 0.25 mm silica plates (60 F254) from Merck were used, and the detection was reached by fluorescence quenching under UV light (λ = 254 nm) or by staining with potassium permanganate reagent (solution of KMnO_4_ (3 g), K_2_CO_3_ (20 g), 5% NaOH (5 mL), and H_2_O (300 mL)) followed by heating to 400 °C.

#### NMR spectra

Measured NMR spectra were, unless otherwise mentioned, recorded at 296 K on a 600 MHz Bruker Avance-III 600 spectrometer with a 5 mm TCI cryoprobe. After prior referencing to the residual solvent signal (CDCl_3_: 7.26 ppm & 77.16 ppm; DMSO-d_6_: 2.50 ppm & 39.52 ppm; DMF-d_7_: 2.75 ppm & 29.76 ppm for ^1^H NMR and ^13^C NMR, respectively), all chemical shifts (δ) are reported relative to residual solvent [53]. Coupling constants were reported in Hz and the signal multiplicities were abbreviated as follows: s (singlet), d (doublet), t (triplet), q (quartet), qd (quartet of doublet), m (multiplet).

#### Infrared spectra

Infrared spectroscopy was performed on a Bruker Tensor 27 FTIR spectrometer including a diamond ATR unit and are reported in terms of absorption frequency 𝜈̅ [cm^–1^].

#### Mass spectra

HRMS was conducted on an Agilent G6545A Q-ToF with ESI, APCI or APPI source coupled with an Agilent 1260 Infinity II HPLC system. If not described otherwise, spectra were recorded using positive ionization mode.

#### Optical rotations

Optical rotation measurements were accomplished with a Perkin-Elmer 241MC polarimeter at λ = 589 nm. A solvent-filled cuvette was used for instrument calibration [54].

## Electronic supplementary material

Below is the link to the electronic supplementary material.

Supplementary Material 1

## Data Availability

No datasets were generated or analysed during the current study.

## References

[1] Alberts AW, Chen J, Kuron G, Hunt V, Huff J, Hoffman C, et al. Mevinolin: a highly potent competitive inhibitor of hydroxymethylglutaryl-coenzyme A reductase and a cholesterol-lowering agent. Proc Natl Acad Sci U S A. 1980;77:3957–61. 10.1073/pnas.77.7.3957. 6933445 10.1073/pnas.77.7.3957 PMC349746

[2] Anke T, Oberwinkler F, Steglich W, Schramm G. The strobilurins–new antifungal antibiotics from the basidiomycete Strobilurus Tenacellus. J Antibiot (Tokyo). 1977;30:806–10. 10.7164/antibiotics.30.806. 563391 10.7164/antibiotics.30.806

[3] Sauter H, Steglich W, Anke T, Strobilurins. Evolution of a new class of active substances. Angew Chem Int Ed. 1999;38:1328–49. 10.1002/(SICI)1521-3773(19990517)38:10%3C1328::AID-ANIE1328%3E3.0.CO;2-1. 10.1002/(SICI)1521-3773(19990517)38:10<1328::AID-ANIE1328>3.0.CO;2-1 29711574

[4] Oxford AE, Raistrick H, Simonart P. Studies in the biochemistry of micro-organisms: Griseofulvin, C(17)H(17)O(6)Cl, a metabolic product of Penicillium griseo-fulvum Dierckx. Biochem J. 1939;33:240–8. 10.1042/bj0330240. 16746904 10.1042/bj0330240 PMC1264363

[5] Gupta AK, Foley KA, Versteeg SG. New antifungal agents and new formulations against dermatophytes. Mycopathologia. 2017;182:127–41. 10.1007/s11046-016-0045-0. 27502503 10.1007/s11046-016-0045-0

[6] Hertweck C. The biosynthetic logic of polyketide diversity. Angew Chem Int Ed. 2009;48:4688–716. 10.1002/anie.200806121. 10.1002/anie.200806121 19514004

[7] Cox RJ, Simpson TJ. Fungal type I polyketide synthases. Methods Enzymol. 2009;459:49–78. 10.1016/S0076-6879(09)04603-5. 19362635 10.1016/S0076-6879(09)04603-5

[8] Chooi Y-H, Tang Y. Navigating the fungal polyketide chemical space: from genes to molecules. J Org Chem. 2012;77:9933–53. 10.1021/jo301592k. 22938194 10.1021/jo301592k PMC3500441

[9] Crawford JM, Dancy BCR, Hill EA, Udwary DW, Townsend CA. Identification of a starter unit acyl-carrier protein transacylase domain in an iterative type I polyketide synthase. Proc Natl Acad Sci U S A. 2006;103:16728–33. 10.1073/pnas.0604112103. 17071746 10.1073/pnas.0604112103 PMC1636523

[10] Crawford JM, Thomas PM, Scheerer JR, Vagstad AL, Kelleher NL, Townsend CA. Deconstruction of iterative multidomain polyketide synthase function. Science. 2008;320:243–6. 10.1126/science.1154711. 18403714 10.1126/science.1154711 PMC2480491

[11] Chiang Y-M, Szewczyk E, Davidson AD, Keller N, Oakley BR, Wang CCC. A gene cluster containing two fungal polyketide synthases encodes the biosynthetic pathway for a polyketide, Asperfuranone, in Aspergillus Nidulans. J Am Chem Soc. 2009;131:2965–70. 10.1021/ja8088185. 19199437 10.1021/ja8088185 PMC2765542

[12] Löhr NA, Rakhmanov M, Wurlitzer JM, Lackner G, Gressler M, Hoffmeister D. Basidiomycete non-reducing polyketide synthases function independently of SAT domains. Fungal Biol Biotechnol. 2023;10:17. 10.1186/s40694-023-00164-z. 37542286 10.1186/s40694-023-00164-z PMC10401856

[13] Liu L, Zhang Z, Shao C-L, Wang J-L, Bai H, Wang C-Y. Bioinformatical analysis of the sequences, structures and functions of fungal polyketide synthase product template domains. Sci Rep. 2015;5:10463. 10.1038/srep10463. 25995122 10.1038/srep10463 PMC5386248

[14] Li Y, Image II, Xu W, Image I, Tang Y. Classification, prediction, and verification of the regioselectivity of fungal polyketide synthase product template domains. J Biol Chem. 2010;285:22764–73. 10.1074/jbc.M110.128504. 20479000 10.1074/jbc.M110.128504 PMC2906267

[15] Throckmorton K, Wiemann P, Keller NP. Evolution of chemical diversity in a group of Non-Reduced polyketide gene clusters: using phylogenetics to inform the search for novel fungal natural products. Toxins (Basel). 2015;7:3572–607. 10.3390/toxins7093572. 26378577 10.3390/toxins7093572 PMC4591646

[16] Awakawa T, Yokota K, Funa N, Doi F, Mori N, Watanabe H, Horinouchi S. Physically discrete beta-lactamase-type thioesterase catalyzes product release in Atrochrysone synthesis by iterative type I polyketide synthase. Chem Biol. 2009;16:613–23. 10.1016/j.chembiol.2009.04.004. 19549600 10.1016/j.chembiol.2009.04.004

[17] Saha D, Fetzner R, Burkhardt B, Podlech J, Metzler M, Dang H, et al. Identification of a polyketide synthase required for alternariol (AOH) and alternariol-9-methyl ether (AME) formation in Alternaria alternata. PLoS ONE. 2012;7:e40564. 10.1371/journal.pone.0040564. 22792370 10.1371/journal.pone.0040564 PMC3391263

[18] Cacho RA, Chooi Y-H, Zhou H, Tang Y. Complexity generation in fungal polyketide biosynthesis: a spirocycle-forming P450 in the concise pathway to the antifungal drug Griseofulvin. ACS Chem Biol. 2013;8:2322–30. 10.1021/cb400541z. 23978092 10.1021/cb400541z PMC3821396

[19] Szewczyk E, Chiang Y-M, Oakley CE, Davidson AD, Wang CCC, Oakley BR. Identification and characterization of the asperthecin gene cluster of Aspergillus Nidulans. Appl Environ Microbiol. 2008;74:7607–12. 10.1128/AEM.01743-08. 18978088 10.1128/AEM.01743-08 PMC2607171

[20] Li Y, Chooi Y-H, Sheng Y, Valentine JS, Tang Y. Comparative characterization of fungal anthracenone and Naphthacenedione biosynthetic pathways reveals an α-hydroxylation-dependent Claisen-like cyclization catalyzed by a Dimanganese thioesterase. J Am Chem Soc. 2011;133:15773–85. 10.1021/ja206906d. 21866960 10.1021/ja206906d PMC3183131

[21] Chooi Y-H, Cacho R, Tang Y. Identification of the viridicatumtoxin and Griseofulvin gene clusters from Penicillium aethiopicum. Chem Biol. 2010;17:483–94. 10.1016/j.chembiol.2010.03.015. 20534346 10.1016/j.chembiol.2010.03.015 PMC2884005

[22] König CC, Scherlach K, Schroeckh V, Horn F, Nietzsche S, Brakhage AA, Hertweck C. Bacterium induces cryptic meroterpenoid pathway in the pathogenic fungus Aspergillus fumigatus. ChemBioChem. 2013;14:938–42. 10.1002/cbic.201300070. 23649940 10.1002/cbic.201300070

[23] Chooi Y-H, Wang P, Fang J, Li Y, Wu K, Wang P, Tang Y. Discovery and characterization of a group of fungal polycyclic polyketide prenyltransferases. J Am Chem Soc. 2012;134:9428–37. 10.1021/ja3028636. 22590971 10.1021/ja3028636 PMC3904230

[24] Li H, Shu S, Kalaitzis JA, Shang Z, Vuong D, Crombie A, et al. Genome mining of Aspergillus Hancockii unearths cryptic polyketide hancockinone A featuring a prenylated 6/6/6/5 carbocyclic skeleton. Org Lett. 2021;23:8789–93. 10.1021/acs.orglett.1c03283. 34747627 10.1021/acs.orglett.1c03283

[25] Yamashita Y, Saitoh Y, Ando K, Takahashi K, Ohno H, Nakano H. Saintopin, a new antitumor antibiotic with topoisomerase II dependent DNA cleavage activity, from Paecilomyces. J Antibiot (Tokyo). 1990;43:1344–6. 10.7164/antibiotics.43.1344. 2175304 10.7164/antibiotics.43.1344

[26] van der Zanden SY, Qiao X, Neefjes J. New insights into the activities and toxicities of the old anticancer drug doxorubicin. FEBS J. 2021;288:6095–111. 10.1111/febs.15583. 33022843 10.1111/febs.15583 PMC8597086

[27] Geib E, Baldeweg F, Doerfer M, Nett M, Brock M. Cross-Chemistry leads to product diversity from atromentin synthetases in aspergilli from section Nigri. Cell Chem Biol. 2019;26:223–e2346. 10.1016/j.chembiol.2018.10.021. 30527997 10.1016/j.chembiol.2018.10.021

[28] Wieder C, Künzer M, Wiechert R, Seipp K, Andresen K, Stark P, et al. Biosynthesis of the antifungal Polyhydroxy-Polyketide acrophialocinol. Org Lett. 2025. 10.1021/acs.orglett.4c04656. 39842789 10.1021/acs.orglett.4c04656 PMC11791885

[29] Langfelder K, Streibel M, Jahn B, Haase G, Brakhage AA. Biosynthesis of fungal melanins and their importance for human pathogenic fungi. Fungal Genet Biol. 2003;38:143–58. 10.1016/S1087-1845(02)00526-1. 12620252 10.1016/s1087-1845(02)00526-1

[30] Geib E, Gressler M, Viediernikova I, Hillmann F, Jacobsen ID, Nietzsche S, et al. A Non-canonical melanin biosynthesis pathway protects Aspergillus terreus conidia from environmental stress. Cell Chem Biol. 2016;23:587–97. 10.1016/j.chembiol.2016.03.014. 27133313 10.1016/j.chembiol.2016.03.014

[31] Jacobson ES. Pathogenic roles for fungal melanins. Clin Microbiol Rev. 2000;13:708–17. 10.1128/cmr.13.4.708-717.2000. 11023965 10.1128/cmr.13.4.708-717.2000 PMC88958

[32] Cordero RJ, Casadevall A. Functions of fungal melanin beyond virulence. Fungal Biol Rev. 2017;31:99–112. 10.1016/j.fbr.2016.12.003. 31649746 10.1016/j.fbr.2016.12.003 PMC6812541

[33] Braga GUL, Rangel DEN, Fernandes ÉKK, Flint SD, Roberts DW. Molecular and physiological effects of environmental UV radiation on fungal conidia. Curr Genet. 2015;61:405–25. 10.1007/s00294-015-0483-0. 25824285 10.1007/s00294-015-0483-0

[34] Lim S, Bijlani S, Blachowicz A, Chiang Y-M, Lee M-S, Torok T, et al. Identification of the pigment and its role in UV resistance in Paecilomyces variotii, a Chernobyl isolate, using genetic manipulation strategies. Fungal Genet Biol. 2021;152:103567. 10.1016/j.fgb.2021.103567. 33989788 10.1016/j.fgb.2021.103567

[35] Xu Y, Vinas M, Alsarrag A, Su L, Pfohl K, Rohlfs M, et al. Bis-naphthopyrone pigments protect filamentous ascomycetes from a wide range of predators. Nat Commun. 2019;10:3579. 10.1038/s41467-019-11377-5. 31395863 10.1038/s41467-019-11377-5 PMC6687722

[36] Watanabe CMH, Townsend CA. Initial characterization of a type I fatty acid synthase and polyketide synthase multienzyme complex NorS in the biosynthesis of aflatoxin B(1). Chem Biol. 2002;9:981–8. 10.1016/S1074-5521(02)00213-2. 12323372 10.1016/s1074-5521(02)00213-2

[37] Löhr NA, Eisen F, Thiele W, Platz L, Motter J, Hüttel W, et al. Unprecedented mushroom polyketide synthases produce the universal anthraquinone precursor. Angew Chem Int Ed Engl. 2022;61:e202116142. 10.1002/anie.202116142. 35218274 10.1002/anie.202116142 PMC9325552

[38] Watanabe A, Ebizuka Y. Unprecedented mechanism of chain length determination in fungal aromatic polyketide synthases. Chem Biol. 2004;11:1101–6. 10.1016/j.chembiol.2004.05.015. 15324811 10.1016/j.chembiol.2004.05.015

[39] Zaehle C, Gressler M, Shelest E, Geib E, Hertweck C, Brock M. Terrein biosynthesis in Aspergillus terreus and its impact on phytotoxicity. Chem Biol. 2014;21:719–31. 10.1016/j.chembiol.2014.03.010. 24816227 10.1016/j.chembiol.2014.03.010

[40] Nakazawa T, Ishiuchi K, Praseuth A, Noguchi H, Hotta K, Watanabe K. Overexpressing transcriptional regulator in Aspergillus oryzae activates a silent biosynthetic pathway to produce a novel polyketide. ChemBioChem. 2012;13:855–61. 10.1002/cbic.201200107. 22447538 10.1002/cbic.201200107

[41] Fujii I, Mori Y, Watanabe A, Kubo Y, Tsuji G, Ebizuka Y. Enzymatic synthesis of 1,3,6,8-tetrahydroxynaphthalene solely from malonyl coenzyme A by a fungal iterative type I polyketide synthase PKS1. Biochemistry. 2000;39:8853–8. 10.1021/bi000644j. 10913297 10.1021/bi000644j

[42] Lo H-C, Entwistle R, Guo C-J, Ahuja M, Szewczyk E, Hung J-H, et al. Two separate gene clusters encode the biosynthetic pathway for the meroterpenoids Austinol and dehydroaustinol in Aspergillus Nidulans. J Am Chem Soc. 2012;134:4709–20. 10.1021/ja209809t. 22329759 10.1021/ja209809t PMC3350773

[43] Wheeler MH, Abramczyk D, Puckhaber LS, Naruse M, Ebizuka Y, Fujii I, Szaniszlo PJ. New biosynthetic step in the melanin pathway of Wangiella (Exophiala) dermatitidis: evidence for 2-acetyl-1,3,6,8-Tetrahydroxynaphthalene as a novel precursor. Eukaryot Cell. 2008;7:1699–711. 10.1128/EC.00179-08. 18676950 10.1128/EC.00179-08 PMC2568069

[44] Tsai HF, Fujii I, Watanabe A, Wheeler MH, Chang YC, Yasuoka Y, et al. Pentaketide melanin biosynthesis in Aspergillus fumigatus requires chain-length shortening of a heptaketide precursor. J Biol Chem. 2001;276:29292–8. 10.1074/jbc.M101998200. 11350964 10.1074/jbc.M101998200

[45] Fujii I, Yasuoka Y, Tsai H-F, Chang YC, Kwon-Chung KJ, Ebizuka Y. Hydrolytic polyketide shortening by ayg1p, a novel enzyme involved in fungal melanin biosynthesis. J Biol Chem. 2004;279:44613–20. 10.1074/jbc.M406758200. 15310761 10.1074/jbc.M406758200

[46] Watanabe A, Fujii I, Tsai H, Chang YC, Kwon-Chung KJ, Ebizuka Y. Aspergillus fumigatus alb1 encodes naphthopyrone synthase when expressed in Aspergillus oryzae. FEMS Microbiol Lett. 2000;192:39–44. 10.1111/j.1574-6968.2000.tb09356.x. 11040426 10.1111/j.1574-6968.2000.tb09356.x

[47] Vagstad AL, Hill EA, Labonte JW, Townsend CA. Characterization of a fungal thioesterase having Claisen cyclase and deacetylase activities in melanin biosynthesis. Chem Biol. 2012;19:1525–34. 10.1016/j.chembiol.2012.10.002. 23261597 10.1016/j.chembiol.2012.10.002 PMC3530136

[48] Breinholt J, Jensen GW, Kjær A, Olsen CE, Rosendahl CN, Romerosa A, et al. Hypomycetin - an antifungal, tetracyclic metabolite from hypomyces aurantius: production, structure and biosynthesis. Acta Chem Scand. 1997;51:855–60. 10.3891/acta.chem.scand.51-0855.

[49] WONG S-M, KULLNIG R, DEDINAS J, APPELL KC, KYDD GC, GILLUM AM, et al. Anthrotainin, an inhibitor of substance P binding produced by Gliocladium catenulatum. J Antibiot (Tokyo). 1993;46:214–21. 10.7164/antibiotics.46.214. 7682212 10.7164/antibiotics.46.214

[50] Liu M, Ohashi M, Hung Y-S, Scherlach K, Watanabe K, Hertweck C, Tang Y. AoiQ catalyzes geminal dichlorination of 1,3-Diketone natural products. J Am Chem Soc. 2021;143:7267–71. 10.1021/jacs.1c02868. 33957045 10.1021/jacs.1c02868 PMC8434754

[51] Wieder C, Da Peres Silva R, Witts J, Jäger CM, Geib E, Brock M. Characterisation of Ascocorynin biosynthesis in the purple jellydisc fungus Ascocoryne sarcoides. Fungal Biol Biotechnol. 2022;9:8. 10.1186/s40694-022-00138-7. 35477441 10.1186/s40694-022-00138-7 PMC9047271

[52] Sidhu YS, Cairns TC, Chaudhari YK, Usher J, Talbot NJ, Studholme DJ, et al. Exploitation of sulfonylurea resistance marker and non-homologous end joining mutants for functional analysis in Zymoseptoria tritici. Fungal Genet Biol. 2015;79:102–9. 10.1016/j.fgb.2015.04.015. 26092796 10.1016/j.fgb.2015.04.015 PMC4502460

[53] Fulmer GR, Miller AJM, Sherden NH, Gottlieb HE, Nudelman A, Stoltz BM, et al. NMR chemical shifts of trace impurities: common laboratory solvents, organics, and gases in deuterated solvents relevant to the organometallic chemist. Organometallics. 2010;29:2176–9. 10.1021/om100106e.

[54] Lippke G, Thaler H. Die Spezifische Drehung des sorbits und des Sorbit-Molybdat‐Komplexes. Starch Stärke. 1970;22:344–51. 10.1002/star.19700221005.

