# Supplementary material for: Biosynthesis of the Paecilomyces marquandii conidial pigment saintopin
Source: Fungal Biol Biotechnol. 2025 Jun 5;12:11. doi: 10.1186/s40694-025-00199-4 (PMC12139152; doi:10.1186/s40694-025-00199-4)
Supplement: Supplementary file 1 — Supplementary Material 1 [file 40694_2025_199_MOESM1_ESM.docx]

**Supporting Information**

**Biosynthesis of the *Paecilomyces marquandii* conidial pigment saintopin**

Carsten Wieder ^1,2,*^, Sarah Galwas ^1^, Rainer Wiechert ^3^, Kevin Seipp ^3^, Alexander Yemelin ^2^, Eckhard Thines ^1,2^, Till Opatz ^3^, Anja Schüffler ^2,*^

^1^ Institute of Molecular Physiology, Johannes Gutenberg-University, Hanns-Dieter-Hüsch Weg 17, D-55128 Mainz, Germany

^2^ Institut für Biotechnologie und Wirkstoff-Forschung gGmbH, Mainz, Hanns-Dieter-Hüsch Weg 17, D-55128 Mainz, Germany

^3^ Department of Chemistry, Johannes Gutenberg-University, Duesbergweg 10–14, D-55128 Mainz, Germany

*Correspondence: [cawieder@uni-mainz.de](mailto:cawieder@uni-mainz.de), [schueffler@ibwf.de](mailto:schueffler@ibwf.de)

**Table of Contents**

[Additional figures 2](#_Toc181196453)

[Additional tables 6](#_Toc181196454)

[Analytical data 10](#_Toc181196455)

[^1^H- and ^13^C{^1^H}-NMR spectra 14](#_Toc181196456)

[References 22](#_Toc181196457)

# Additional figures


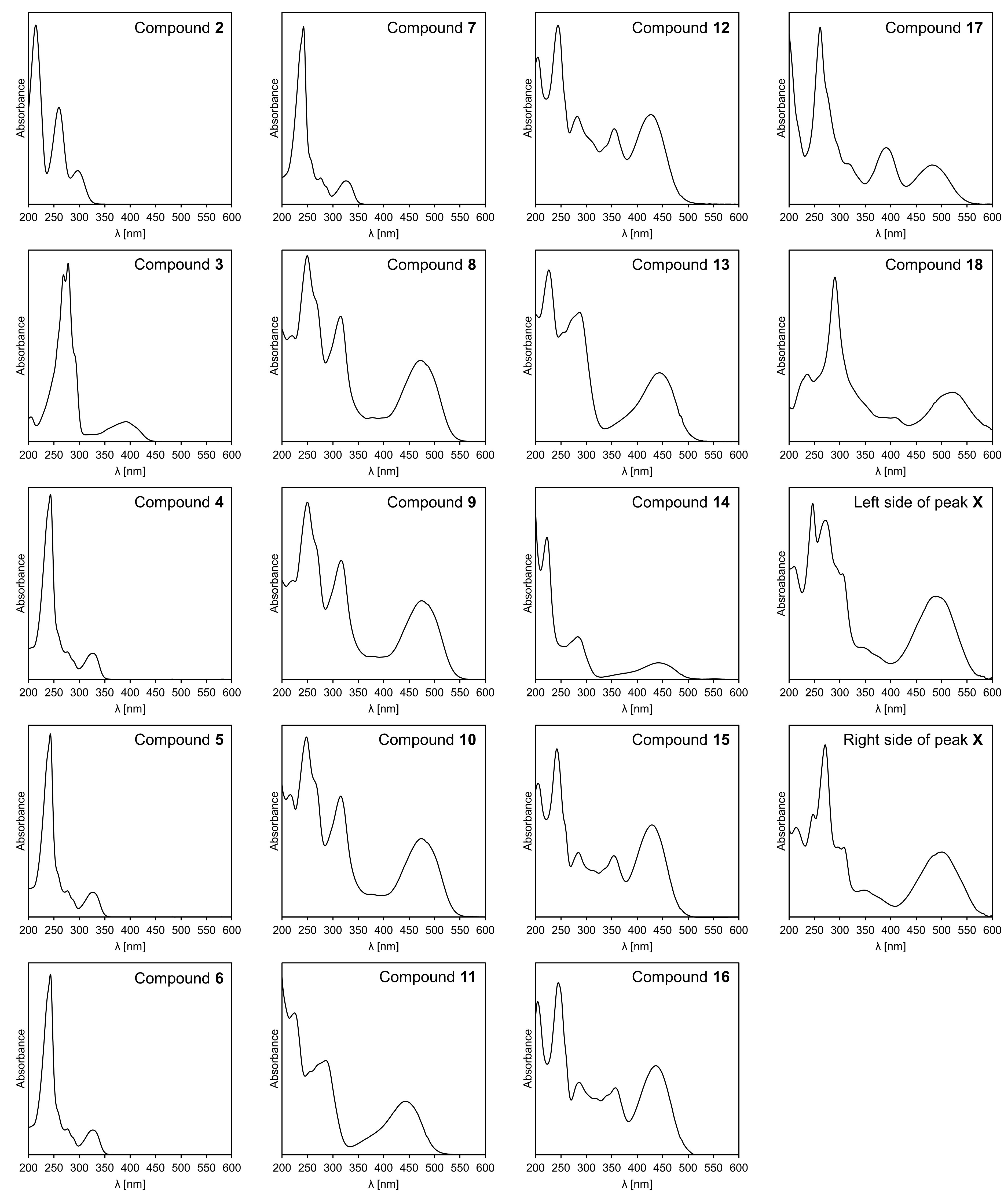


Figure S. 1: UV/Vis-spectra of purified compounds **2**–**7**, detected compounds **8**–**18** and peak **X**


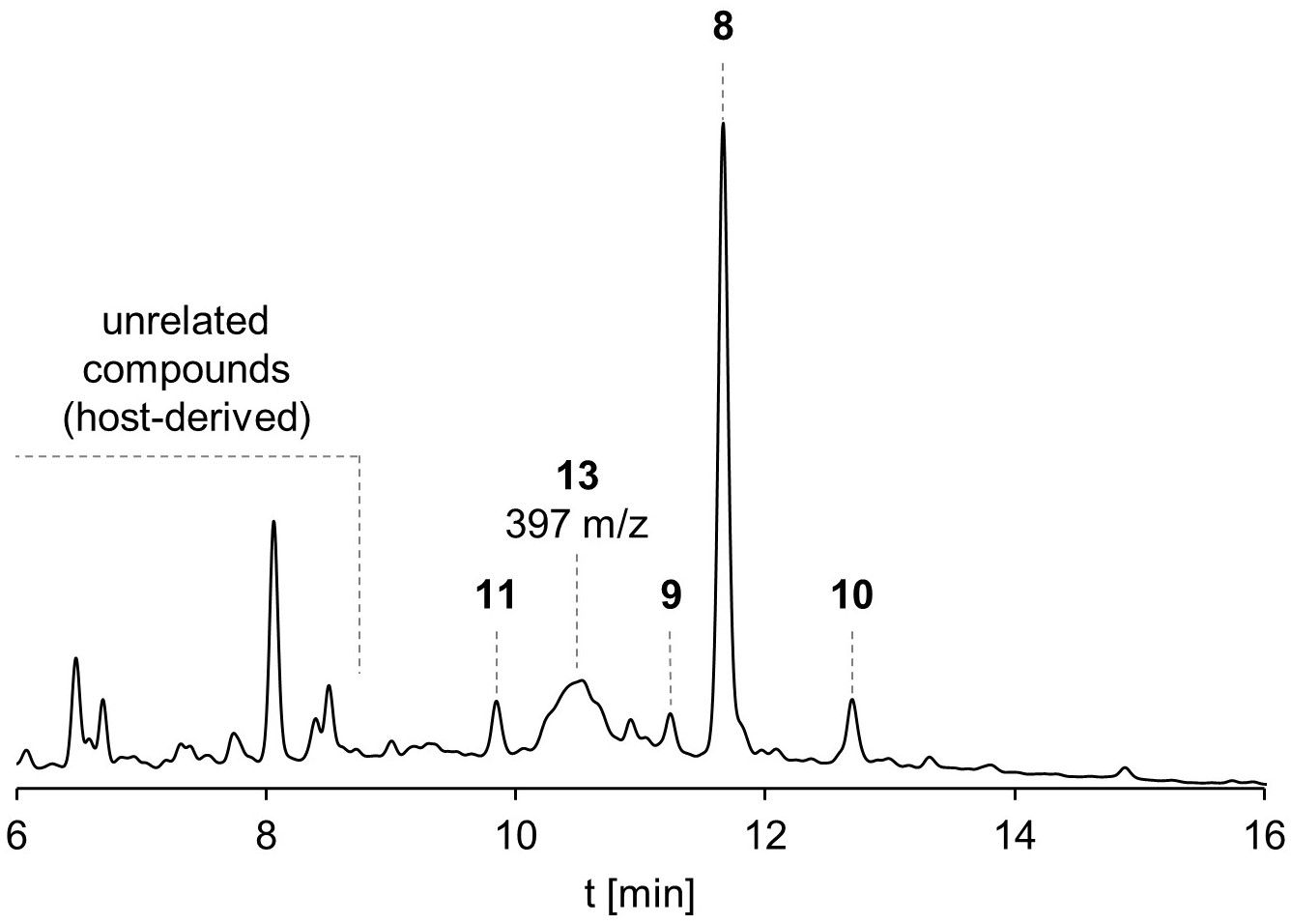


Figure S. 2: Magnified chromatogram (250 nm) of the OP12_*stpA* culture filtrate extract and assignment of an additional presumed shunt product (**13**). Mass data was recorded in negative mode. The UV/Vis spectrum of **13** can be found in Figure S. 1.


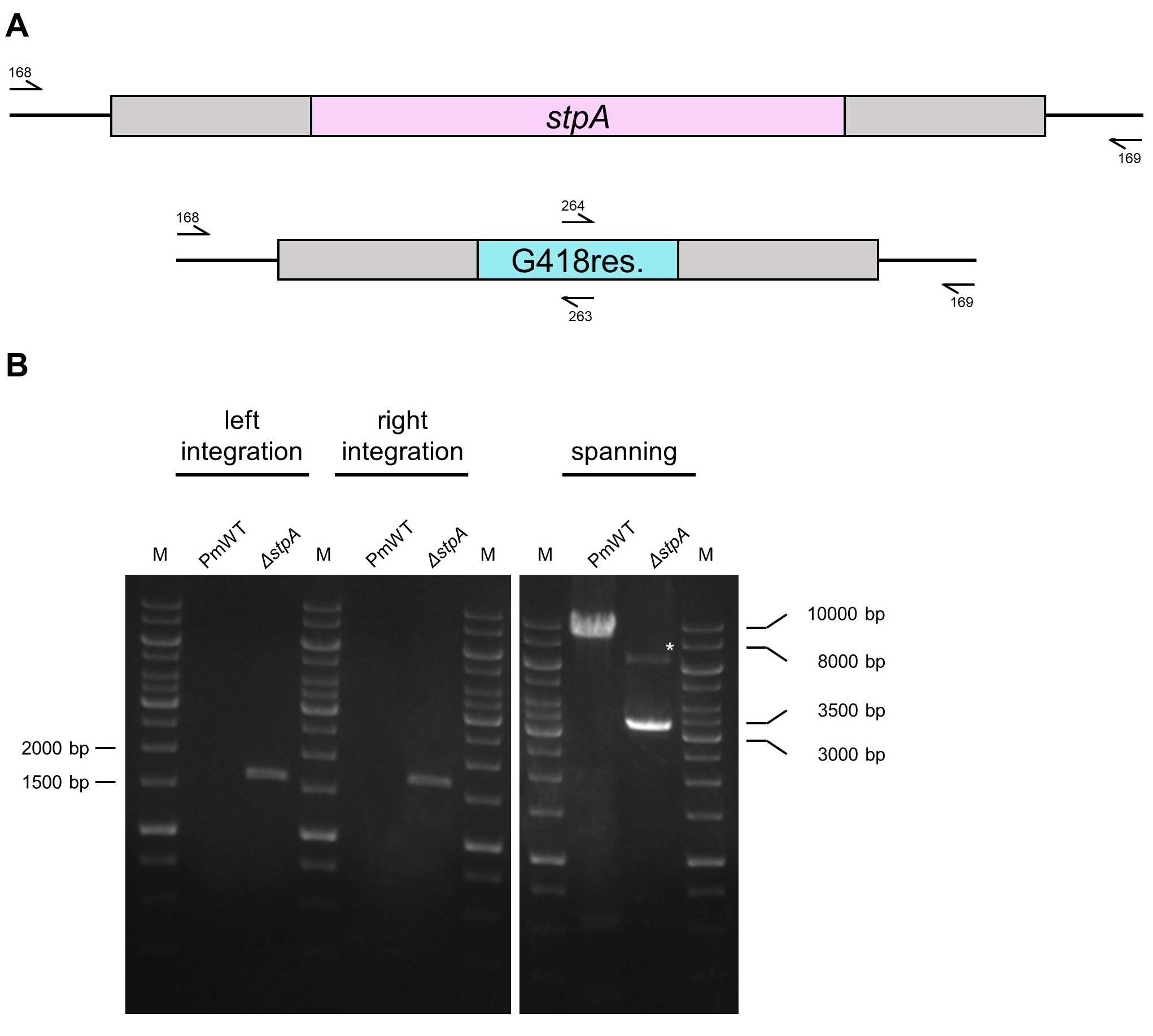


Figure S. 3: Verification of stpA-deletion. (A) PCR-strategy for verification of deletion. The grey boxes represent the flanks used for homology directed deletion of stpA. Primers 5’- and 3’- of the flanks and within the G418 resistance cassette were used to amplify sequences from PmWT and *ΔstpA* DNA. (B) PCR-amplicons observed are in accord with the deletion strategy, confirming identity of the ΔstpA deletion mutant. * denotes an off-target amplicon


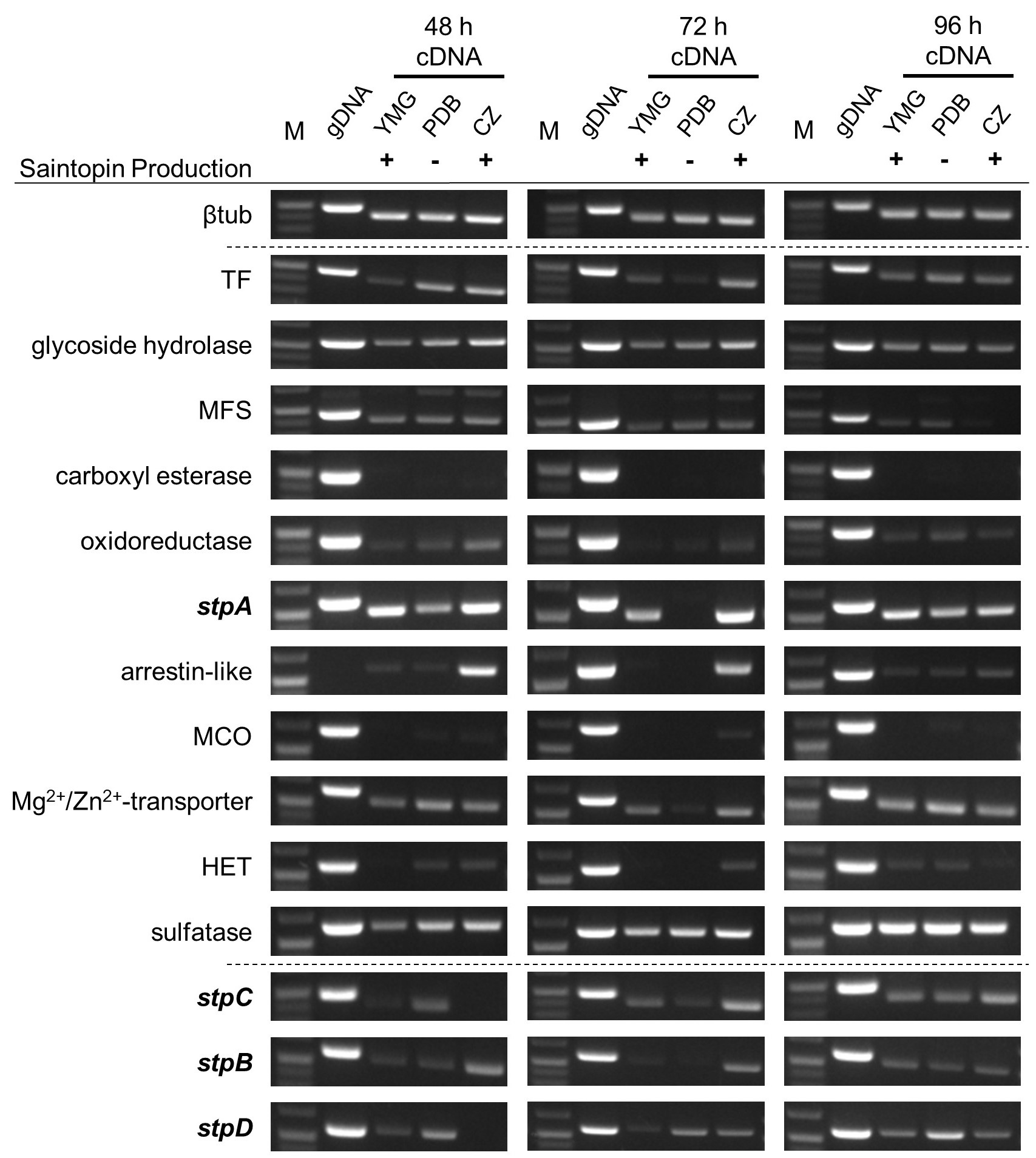


Figure S. 4: sqRT-PCR of stpA clustered and non-clustered genes. *P. marquandii* was inoculated cultivated in either YMG, PDB or CZ media for 4 days shaking at 120 at room temperature. After 48, 72 and 96 hours, 50 mL samples were taken for isolation of RNA and evaluation of saintopin (**1**) production by HPLC. RNA was reverse transcribed into cDNA and cDNA concentrations adjusted to produce similar intensity β-tub signals. Primers were designed intron-spanning as far as possible. Homologs of other napthacenedione biosynthesis genes are highlighted in bold.


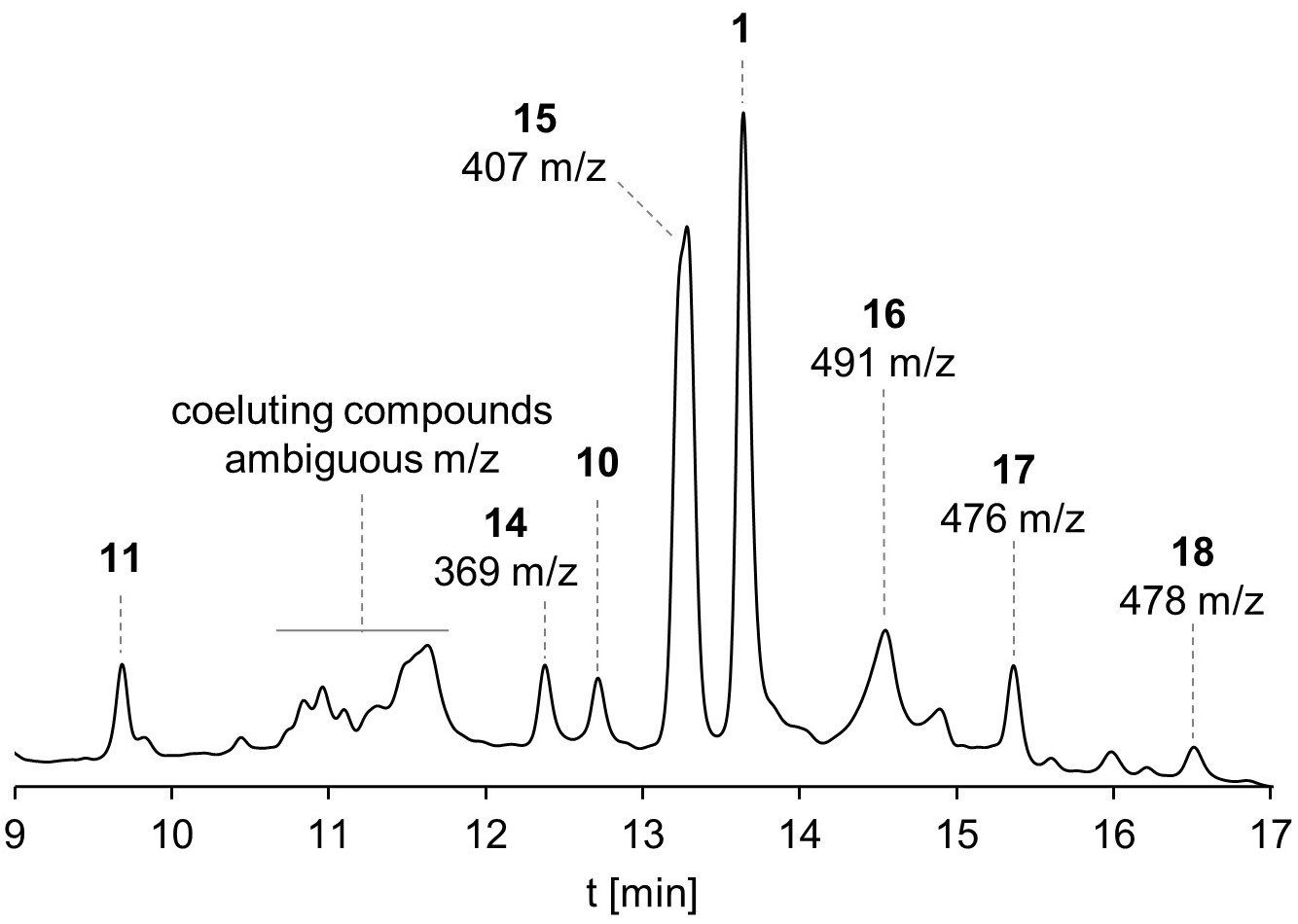


Figure S. 5: Magnified chromatogram (250 nm) of the OP12_*stpAC* mycelia extract and assignment of some additional presumed shunt product (**14**–**18**). Mass data was recorded in negative mode. The UV/Vis spectra of **14**–**18** can be found in Figure S. 1.


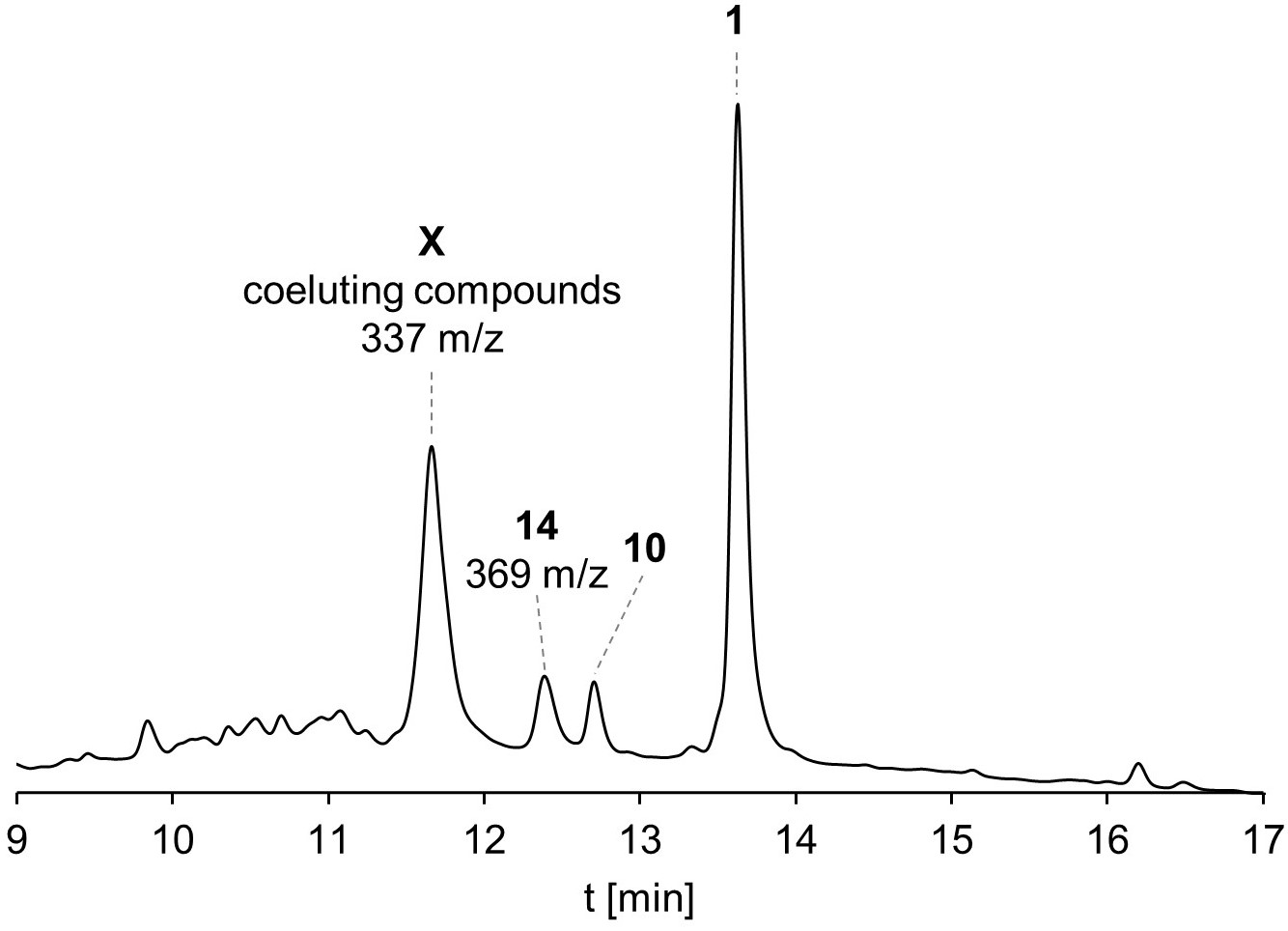


Figure S. 6: Magnified chromatogram (250 nm) of the OP12_ *adaA/stpC* mycelia extract and assignment of some additional presumed shunt product (**14**, Peak **X**). Mass data was recorded in negative mode. The UV/Vis spectra of the left and right side of Peak **X** can be found in Figure S. 1.


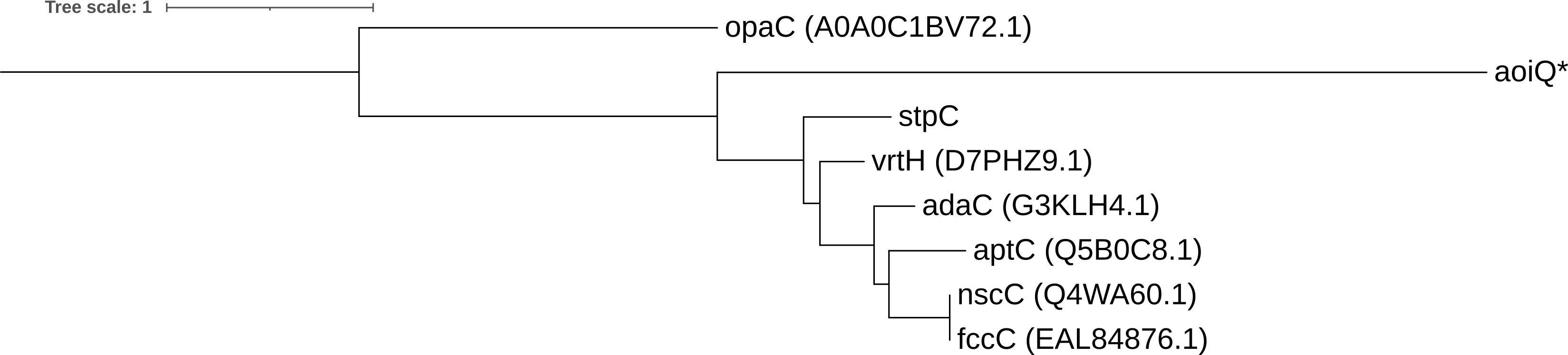


Figure S. 7: Phylogenetic analysis of flavin-dependent monooxygenases involved in naphthacenedione and dichlorodiaporthin biosynthetic pathways. The amino acid sequences of the flavin-dependent monooxygenases were aligned using the MUSCLE algorithm [1] on the EMBL-EBI webpage. A maximum likelihood phylogenetic tree was created using W-IQ-Tree [2] and visualized with iTOL [3], rooting the tree at the midpoint. *opaC* was included as an outgroup. *only the FDH domain [4] of *aoiQ* was used for the alignment

# Additional tables

Table S. 1: Mutant strains used in this study

| **Strain** | **Genotype** | **Produces** | **Source** |
| --- | --- | --- | --- |
| OP12 *pyrG^–^* | PamyB:terR_ptrA; *pyrG^–^* | / | [5] |
| OP12 3Δ | PamyB:terR_ptrA; *pyrG^–^*, *ΔpabA*, *ΔargB* | / | [6] |
| OP12 empty plasmid | PamyB:terR_ptrA, *pyrG*^+^ | / | This study |
| OP12 3Δ empty plasmids | PamyB:terR_ptrA, *pyrG^+^*, *pabA^+^*, *argB^+^* | / | [6] |
| OP12_PmPKS1 | PamyB:terR_ptrA; PterA:nHis-PmPKS1_URA | **2** | This study |
| OP12_PmPKS2 | PamyB:terR_ptrA; PterA:PmPKS2_URA | **3** | This study |
| OP12_*stpA* | PamyB:terR_ptrA; PterA:nHis-*stpA*_URA | **8, 9** | This study |
| OP12_PmPKS4 | PamyB:terR_ptrA; PterA:nHis-PmPKS4_URA | **4-7** | This study |
| OP12_PmPKS5 | PamyB:terR_ptrA; PterA:nHis-PmPKS5_URA | / | This study |
| OP12_PmPKS6 | PamyB:terR_ptrA; PterA:nHis-PmPKS6_URA | **2, 7** | This study |
| OP12_*aptA* | PamyB:terR_ptrA; PterA:*aptA*_URA | **11** | This study |
| OP12_*adaA* | PamyB:terR_ptrA; PterA:*adaA*_URA | **8, 9** | This study |
| OP12_*stpAB* | PamyB:terR_ptrA; PterA:nHis-*stpA*_URA, PterA:*stpB*_pabA; *ΔargB* | **8, 9** | This study |
| OP12_*stpAC* | PamyB:terR_ptrA; PterA:nHis-*stpA*_URA, PterA:*stpC*_argB(mut); *ΔpabA* | **1, 10** | This study |
| OP12_*stpABC* | PamyB:terR_ptrA; PterA:nHis-*stpA*_URA, PterA:*stpB*_pabA; PterA:*stpC*_argB(mut) | **1, 10** | This study |
| OP12_*aptA/stpC* | PamyB:terR_ptrA; PterA:*aptA*_URA, PterA:*stpC*_argB(mut); *ΔpabA* | **11** | This study |
| OP12_*adaA/stpC* | PamyB:terR_ptrA; PterA:nHis-*stpA*_URA, PterA:*stpC*_argB(mut); *ΔpabA* | **1, 10** | This study |

Table S. 2: Oligonucleotides used for cloning

| **Oligo** | **Sequence** | **Purpose** |
| --- | --- | --- |
| oCW126 | Accatgcatcatcatcaccatcaccatggagcttacactagccatgtcct | Amplification of PKS1 for cloning |
| oCW170 | cggttcagattgaaatcactgctgttatctcatgttgtgtaagcaatat |  |
| oCW273 | catttaacaaacttctcatcacagcaccatgggacatccagggagcttgt | Amplification of PKS2 for cloning |
| oCW277 | ccttacgccttccattcatc |  |
| oCW278 | gatgaatggaaggcgtaagg |  |
| oCW143 | cggttcagattgaaatcactgctgttatcctaagccatagcctggcgaa |  |
| oCW172 | accatgcatcatcatcaccatcaccatggatacgatacggttggcacagc | Amplification of *stpA* for cloning |
| oCW135 | cggttcagattgaaatcactgctgttatcttagcagttctggtcaac |  |
| oCW173 | accatgcatcatcatcaccatcaccatggagtcaccgccgcagacgtcgg | Amplification of PKS4 for cloning |
| oCW174 | cggttcagattgaaatcactgctgttatcctataaaaaggcaataatga |  |
| oCW175 | accatgcatcatcatcaccatcaccatggagccaagatattttgccacgt | Amplification of PKS5 for cloning |
| oCW176 | cggttcagattgaaatcactgctgttatcttagggcttatcagacatga |  |
| oCW177 | accatgcatcatcatcaccatcaccatggatatcgatctgagacaacaga | Amplification of PKS6 for cloning |
| oCW178 | cggttcagattgaaatcactgctgttatctcacttgcctacattattgg |  |
| oCW164 | catttaacaaacttctcatcacagcaccatgGCGACACCCAGTCCTCTCG | Amplification of *stpB* for cloning |
| oCW165 | ctatacggttcagattgaaatcactgctgcCTAGTCAGAATCAGACACCA |  |
| oCW249 | catttaacaaacttctcatcacagcaccatgGCCATCAAGAGCCCAGCCG | Amplification of *stpC* for cloning |
| oCW250 | ctatacggttcagattgaaatcactgctgcctaagccgtcactgttccgg |  |
| oCW331 | Catttaacaaacttctcatcacagcaccatgtctgcccctacgaagctgg | Amplification of *adaA* for cloning |
| oCW332 | ctatacggttcagattgaaatcactgctgctcagcaatactgctccaacc |  |
| oCW333 | Catttaacaaacttctcatcacagcaccatgaaagacaatacgcatagca | Amplification of *aptA* for cloning |
| oCW334 | ctatacggttcagattgaaatcactgctgcctaacagtactgctctaacc |  |
| oCW151 | aggtcgactctagaggatccccGCGGCCGCgcgaacatgtagattggtgg | Amplification of left flank for pKO_stpA_G418 |
| oCW152 | CAAGCCCAAAAAATGCTCCTTCAATATCAGcaacggacttgtcacctctc |  |
| oCW153 | ttattataaggagaggtgacaagtccgttgCTGATATTGAAGGAGCATTT | Amplification of G418 cassette for pKO_stpA_G418 |
| oCW154 | atggttgaggtgttaccatggttgaccgacCATCATGCAACATGCATGTA |  |
| oCW155 | CATCAGACAGTACATGCATGTTGCATGATGgtcggtcaaccatggtaaca | Amplification of right flank for pKO_stpA_G418 |
| oCW156 | agtgaattcgagctcggtacccGCGGCCGCgacaagagtccagtccgagt |  |

Table S. 3: Oligonucleotides used for sqRT-PCR

| **Oligo** | **Sequence** | **sqRT-PCR Amplicon** |
| --- | --- | --- |
| oCW121 | ATGATGGCCGCTTCTGACTT | β-tub |
| oCW122 | CTCCTCTTCCTCCTCGTCAA |  |
| oCW218 | ctggattgcattctggct | TF |
| oCW219 | gatcgaagccggataaga |  |
| oCW220 | gacgtcaacacatacagc | glycoside hydrolase |
| oCW221 | ccagactgctacgaatct |  |
| oCW222 | gcaaaccagaagaagcca | MFS |
| oCW223 | cgtacaagctgaacttgg |  |
| oCW224 | gctcaatggatatgcgct | carboxyl esterase |
| oCW225 | cacgcggttgttcaagaa |  |
| oCW226 | ggaatcagttgcgtgatg | oxidoreductase |
| oCW227 | cggcatcggttcatacat |  |
| oCW228 | gatggcaagtcgatgcat | *stpA* |
| oCW229 | ctatctcgacaaggtgac |  |
| oCW230 | gccgccatcgaaattaac | arrestin-like |
| oCW231 | ccttgatagtaggtacgg |  |
| oCW232 | gttctcggagatcaacac | MCO |
| oCW233 | ggtatatctcgctctgca |  |
| oCW234 | caacatataccgcgccta | Mg2^+^/Zn^2+^-transporter |
| oCW235 | gtagtccttgtcgtactc |  |
| oCW236 | ccacttatcaacccacga | HET |
| oCW237 | gtggtagaagtgcatcca |  |
| oCW238 | ccttccctctcatagtca | sulfatase |
| oCW239 | cgtttgcccttcatcatg |  |
| oCW240 | GACGGCTGTGTTTGAGAA | *stpB* |
| oCW241 | GATACGACGAGAGCTCTT |  |
| oCW242 | GTCCAGGCTGTTCATGTA | *stpC* |
| oCW243 | CCTCTGTCTCACGTTGAA |  |
| oCW244 | GCCAAGGAATCGTACAAG | *stpD* |
| oCW245 | GCGCCTTCTCAAAGTCAA |  |

Table S. 4: ITS sequence of *P. marquandii* IBWF 003-21

| **Fungal strain** | **ITS sequence (ITS1F+ITS4)** | **BLAST hit** | **Identity/E-value** |
| --- | --- | --- | --- |
| IBWF 003-21 | GGAAGTAAAAGTCGTAACAAGGTCTCCGTTGGTGAACCAGCGGAGGGATCATTACCGAGTTTTCAACTCCCAAACCCACTGTGAACATATACCTTTGTTTTCGTTGCCTCGGCGGTTCGCGCCGCCGGGTGACACCTAAACCCTGATTTTAATTACAGAAGTCTTTCTGAGTAAAAACATTCTAAATGAATCAAAACTTTCAACAACGGATCTCTTGGTTCTGGCATCGATGAAGAACGCAGCGAAATGCGATAAGTAATGTGAATTGCAGAATTCAGTGAATCATCGAATCTTTGAACGCACATTGCGCCCGCCAGTATTCTGGCGGGCATGCCTGTTCGAGCGTCATTTCAACCCTCAAGACCCCTTCGGGGGACTTGGTGTTGGGGACCGGCACAGGGGCCTGCCTGCTTGTCTTGCAGCGCCCTCGCCGCCCCCGAAATGAATTGGCGGCCTCGTCGCGGCCTCCCCTGCGTAGTAGCACAACCTCGCAACGGGAACGTGACGGCGGCCACTGCCGTAAAACAACCCAAACTTTATTAGAGTTGACCTCGAATCAGGTAGGAATACCCGCTGAACTTAAGCATATC | *Paecilomyces marquandii* | 95.77%/ 0.0 |

Table S. 5: Predicted gene sequences of *P. marquandii* nrPKS and accessory genes. Predicted Introns are underlined

| **Gene** | | **Predicted gene sequence** |
| --- | --- | --- |
| *stpA* | atgtacgatacggttggcacagccatggccgacggcaccccagccaggctcgtgtactttggaaacgagttccccaatgatgatctcagcgatatctttcgcaagctgcaccagcatagcaaggatcggaggttccgcctgctgtctgcttttcttgacgaagtaattctcgttctgcagcaagaattcgcaaaactcccccaccatgttcgaagccaggtgcctcactttgacaacatcgtgaccctttcagagatgggattccttcgggagctcgggctcggggctgccatggaaagcgctttcctcctcacgctgcaagttggtttgttcattgggtaagtcaagtcaccatgaacctcaagtcattgtcggcgcgaggctaatgaagaaaacaagacaccacgaggcaaaggatcaagagctcaatctgcccaagggtcccacgatgctggctggtctcggcgtcggactcttcactggcgcatccgtagctctgtctacctctcttgcagaggtggtcaagaatgcggccgagctgctgagggtttccttcagactcggagtctacattggcgatttcagcagcaagttggagtcgccacagcctgacggcagcctccagagctggtcgcatgtcatcaccggcatgacggaagagtccgtgagcagcgaggcgtctcgactcaacgaagagctcgggagccacgccatctccaagctcttcatcagcgccgccgacaagacatccgtcagtctcagcgggcctccttctcgaatcaaggctgctttccagcactcgagcgagcttcgatactccaagtccatccccctgcctgtctacgatggactttgccacgccaagcacgtctatggccagagggactacgacgccgtcatggacagcgagaactcgctcgttcccatgtcgcgcaagctgcagcttccgctcatctcgcccaggacgggcaagccgttcttggccaccgaggcaggcgacctgatgaaggagattgccagcgagttgatgaccagtgccatctatctcgacaaggtgacggacggcatcctcggccacgtcgactcggtttccaccgccgacgagctctatctcgccacgttccgcacttccatcgccttcaagggcattctgcagactctggagaacacgttcccagacgtgaaaatcctgaagagcgacatggcggactgggtccatgaagactttggcgacagacggccaggtaatgtcggatgctccaagcttgccattgttggtatggcttgccgcatgcccggaggcggtaatgatcttgaccagttctgggagctcatggaacagggccgggatgttcacacgactgttccccttgatcgatttgatctggcgactcattacgacccttctggcaacactgggaatgcggctacgacgccgtttggaaactttatcgaccgtcccggctttttcgacgctgcctttttcaacatgtctccaagagaggtaattcccccccttttccccaatcttcttagcctccaaagtggccgacatcatactaacagcgtctggtgtaggctgagcaaaccgatcccatgcatcgacttgccatcgccactgcctatgaagccatggaaatggccggcatggtcacaggacgcaccgcctcgacacgccgtgagcgcattggcacttactacggccaggccagtgatgattggcgtgagctcaacggcgcgcagaacatcggcacgtacgcagtcccgggcggcgtgcgaggcttcatcgcaggacgcatcaactacttcttcaagttctcgggtccgtccttctgcgtcgataccgcctgctccagctccatggctgccatccagctagcttgcacggcgctctgggccggtgagattgacaccgccgtggccggaggcgtcaacatcatcaccgaccctgacaactactgcggcttgggcaatgcccatttcctgtccaagacggggcagtgcaaggtctgggacaaggacgccgatggctactgcagagccgacgccatcggatccgtggtgctgaagcgcctggaagatgccgaggcggacaacgacaacatccttggtgttgttaccgccgccgcgacgaaccactgcgccgatgccatctccatcacgcatcctcacgcaggacatcagaaggagaactaccgtcgtgtgctccataatgccggcgtgaaccccctcgatgtcagctttattgagatgcacggcacgggaacccaggctggcgatgccatggagtccgagtccgtcttggacgtctttgctcccttgcgacctcgccgccgcgctgatcagaagctccttctcggcgcggtgaaaagtaacattggacattctgaagcagccgcaggcgtctcctccttgatcaagatgctcctgtgcttcgaaaagagcctgattcctccccacgttggcatcaagacggagatcaacccgcgcatccccaaggacttggatcgccgcaacgccaacatggccatggagttgacgccgtgggttcgaccagccgggaagaagcgcattgccatggtcaactcgtttggtgctcatggcggcaataccacgctgctgcttgaggatccgtctgagagagatagaccgcgtctcagcctcgagagtgccgatggtcgtgctttataccccatcgtcatctctgccaagagcaagaagtctctgcaagccaatatcgaaggtcttctcggctacctcgagaagaaccccggattggacctggccgacgtgtcttacaccacctgtgcacgacgcactcactacaatctgcgtgttgcaacttcggcttccaccgtttctggactgcagaagttcctccgaaatgctattgacaacaaggtcggcctcgagaccaaggctatcccgcccaatattccctcggtggtgctcacctttaccggccagggtgcttcttacaagggcatccgtcaagacctgtttgacgaggtccccttcttccgtgaccaagtccttcaacttgaccagctcgttcagcgcttgggcttcccatccgttgtcccagccatcactggcagtgatgacgacgaggtccaatctcccgtcattagccagctcagcatcgtggtcttggagattgccctcgctcgattctggtcctacatgggcgtgaagccgagtgctgtcatcggccacagcctgggagaatacgccgcccttgccgtcgctggtgtcttgtccgcctcggatgttttgtacctcgttggtcggcgtgcacagattacacaggagctatgcactccttatagccgcgccatgctgtccgtcctgggagatcttgacgatatcacccaggtgttgaaggctagcccagagaccaaggccgtcgagtacgaggtttcctgccagaatactcatgtcgataacgtgctgggagcttcccgagaggatattgaatctattcaaaaggtcctcgaggccaaggccttcaagtgcacgcgtctggaacttccctttgctttccacacttgccagatggacgctgtcattgacgagctcgaggccctggccgagactgttcctttcaaggcaccgagtatccccgttctgtctaccatgcttggaactgccgtgtttgacggaaagaccatcaacgccaactacctccgccgtcagacacgcaacactgtcagatttgcagacgctattgaggctgctcgtgacatgggcattgttgatgaccaaaccgtctgggttgacgttggtcctcaccccgtctgcgtcggcttcgtgaagaagctcattcctacagcaaggattggctcgtcttgccgacgcaacgaggacaacatctccaccgtcgtaaagactctggtcaccctgcacgctgccggcatcactccccactggaacgagtactaccgacacaacgagacagcttacactctgttgcatcttcccaagtatgcttggaacgagaccaactactggattccgtactacggcacgtggacccttgataaggctttcgtcaagttcggacgcaaagacggcagcgtccctgcgcccgtctctgctgctcctagtttccgcacgtcgacgattcaccaagtcaccagtgaaaccatcgaagcctccacggcgtctctccacgtcctctctgatatccagcatcccgagttccttgctgctgtttacggacacacaatgaacaactgcggcgtggccacctcttccatctggacagacatggcatgggccgtgggtgagtacctgtacaagaaactacagcccgacgtcaaggaagtccacatgaacatccttgatctcgaggttctccacggccaggtggccagcaagaccaagggcgcctaccagccgctggctctcgaagccaacctggacttggacacgcaaatcatgtcgctggcctggtacgacgtcagtgtcgagactggcgagcgtgacgccgagagcttcgctactgcttccgtacggttcgaggacccggacgtctggacttccgagtggaatcgccagacgcacctggtgcagggacgcatcgagaccctgcagcagctcgccaacgagaacaaggccaaccgcatctccaagcgcatggcctacaccctgttcaagaaactcgtcgactacgccgagcactaccgcggcatcgacaacatgatcctccacgagtacgaggcagttgcggacatcacgctggcaaatgaccgtcacggcacatggcacactcctcctcactggatcgacagcgtctgccatctcgccggcctcatcatgaacggcagcgacgcgtccaacaccgacgactacttctacgtcacgcccggtgcggacgcgttccgtctgctgaagcccctcgaggcgggcggcaagtacaggagctacgtgcgcatgttccccgtccccatcgaggcgggaatgcacgccggtgatgtctacatcctgcaagacgacaccatcgtcggtgtcttgacgcagatccgcttccgtcgcgtacaccggctcctgctggaccgcttcttctctgccccaacgggagacaaggtcaagaaccgcgacgctcatcagactcggcccgctgcgtccgcgcctcccaagaagacagctgctcctgcgcccgtgaaagctgcccctgcccctgcgccggcgcccaagccagcgcccaaggctgccccgctggtcatgcaaatccacgagactgcgagcgtgagcgatagtctgtccagctcttctacgctgtctagcagcgaggcaagccccatgggcaacacgacgcctcaaacgggtaacactacccctcagacgggcaacgttacgcccaagatggaggagctggatacgggtgttgtcggccagtgtctccagatcatgtcccgcgagaccaacctcgagatgagcgagctgtccgccgacgctgcctttacgcatctgggcgtcgactcgctcatgtcgctggtgctgtccgagaagttccgcaacgagctcggtgttgatatcaagagctccttgttcctcgagtgtcctaccgtcggcgagttcaaggagtgggttgaccagaactgctaa | |
| *stpB* | atggcgacacccagtcctctcgaagaatcattttggcaggagtacctatcagaccaggcggctaaactgccgcctctgtctcacgttgaagatgtaacggaccgggtggttcgtatcatgggtggaaatccagggacgatgaagctccagggcacaaacacatatctcgtcggcacaggacagtctagaattctcatcgacacaggcgaagtatgaaaaaagttcacaaatctctcatcatttcagtacactgacctggtttcagggctccccagagtggattcaacgcctcatccgggtgcttcaagatcgtggcctcgacatctcccatgtcctactcacccactggcacagagaccacacgggcggcgcgcccgacctcgtagcctttgatcccagcttcgcgcacaggatctacaagaaccagccggaccgtgaccagaaccccatccaagacggacaggtcttcgccgtcgaaggcgccaccatccgcgccgtcttcacccccggccacgcggtagaccacatgtgcttcctcctggaggaggaaaacgccctcttcacgggcgacaacgtcctcggccacggcttctccgtggtgatggatctggccgtctacatgaacagcctggactacatggccgccaagggatgcgcgacgggatacccgggccacggcgccaagattgccaacctgcctgccaagatgcacgagtacatccaccacaacgaggttcgtatccagaaggtcctgtctgcgctgacctggaagggcacggggatgaagggcgggatgaccctgcaggagattatacgctccatctacggcgacgtgcagggggacattgcggactatgcgctggcgcccttcttgacgcagatattgtggaagttggcggaggatggcaaggtcgggttctcgccgggggagcccaagaaggcgaggtggttcgggctggggggtgttcgcaaggcgacttctattgctgcgtcggtggtgtctgattctgactag | |
| *stpC* | atggccatcaagagcccagccgcccccgtcctcatcatcggcgcaggtttgtcaggcctcgccgccgggcgaattctggcaaaccacggcgtcccaactatcgtgttcgaagagtcctctccggagctcggtgagagctttgccatgggactgcgcgactggtgctaccagccacttctcgaggctcttggcggcgtgtccctcaagagcatgatcaaggccgtggccccggatcgacacgttggcggaagcggcctggtcgatttctgtatgcgcgacaacgcgactggcaacattctcgtaacaactcccgacgataagaggccggtggtgacgcgggcgaaccgcaatgccgttcgcgcttggctcgccgactgcggcgacgatgacctggatgtgcgataccaccacaagttggcgagcgtatctggcaccctgggaaacatgacggctgtgtttgagaacggagcgcagtacaacggctgtttgatcatcgctgccgatggtgttgactctgcaggtgagtgacgtgacttacacggtcaaaacttgcctccctggcaactatcaccagacacttttcttacacacttgtacagttcgcactcagctgctgcctggcctcgttccccagattctccctgcggtaatctacaacggcaggttccagctatcgcgccaagagttcgaccagcacatcaagccacgcacaaacggctccaacgtcctctcgggcatcggcgacggcttcaacaccaacatcacggtgtgcaacatgaccgacgccaaggtcaacctcgactggtcgtactcgagacgcgctcgcggcgacaccgacgccttgttcgacaactcggaaggcgccaagacccggggcgtgacgggcaccatccccgaagcgctggtggaagagctctcgtcgtatcagctggcggatccttggggccggtacgtcaacgccgaggctatcaagcagcattccgtcttccagtgggtcagccgatgcgtcttcgtccccacggaggaggccaagaagaatggacagcagggcgtggtctttgtgggcgatgcgtggcacacgatgcctgtgttcagcggcgaggggggaaaccacggcgttctggatagtgttgagctcgccatggccatcgtggagaaggattcgctggagcaggccatctctgcgtactatgacggggcgcgccatcgtgtcgaggaggcggttaagaggtccaagacgcgcttcttctggctgcacaggccgatggcagagtggcaggaggtttcggagaagagaaagatggcggaggaacgggttgccaaggccggaacagtgacggcttag | |
| *stpD* | Atgcctcctccggcatcaaccgccacggccgagtccaagccgaaggccaaagcaaaggcgagtgcaaaggccaaggaatcgtacaagacaaaacgcttccactacgatcactgggttctcggcatcgtcagcacctacgcctgggggtgtcccacgtcgcgatacaccctgcctcagtacagggcaaacatcggcgagaaccatctcgacattggcatcgcaacgggctactacctccgccacggcggcatcccactcacaaccaacatcaccgtcgtggacacgaacaagccggccatggagtacgccctggagcgtctcggccgtcgtgatgcgcgcgccatcgtcgccgacatcctgaagccgctccccatccaggacaagttcgactccgtctccatgtactacctcctcaactgcatcccagccggcgtcgagcacaagtgcgctgttttctcgcacatcaggaacaacatgacgcccaatggtgtcattcatggcgccagcattgtgggaaagggcgtccgggtggacaataagtttgctgcgcgaatgcgagggaagcttctggcggcgggaatctttcagaacaaggaggacagcccgtttgactttgagaaggcgctgagacaaaactttcacgaggtggaggtgagggttgttggcaccgtgtttttgtttcgggcggcctgtccaagattggatgacgcagatgccacatcgccagattcttag | |
| PKS1 | atggcttacactagccatgtcctcctattcccccatgaacacacggccttgctcgaggccatacacgacttgagcgtgcgagcccgaactagacccaagcttcgcacctttctcgacaaagcgtctcgagttctacacagagaggttgtggctctgacacggccagagcgagacagcattggcgaattcgacgatcttgtcgaccttgccgagcgccatgtgagaaaacctgatgtggtagttgaaactgccctcttgacatcttttcagatcggtcagcttttggtgtatgtcttacaagaatatagctataacagacaggcctacatagtgctagagctaactgtttcttcagcttcgcagagaatcaccctggagcgttgtcagacaaaccagtagacacctttttccctgttggatatggcatgggattgattgcagccgcagtcgctgcaacagcaacgactgaggatgctattatcgccttgggtttggaagccgttgctgttgccgctcgtctggctgccgatttgcaacggacaagtaaggacattgaggattcggaggacgcctggtcgcgtgtaattgcaggtgttggccaggaggaactcgaacagcaactggcaatggctaatgctaattgtcggccactccaaactgcatatatcgcccagattttgcctgatgccattgtcgtcatgggcccgccatcaaccctcgacacgctctcccatggccagacctggaggatggcccaacccacagcacggtctaccgttccggcgcgatgcatcatgttcggccctcatctgccccctatagacgatgccaaggttgtgggaaccgcgccagtcctcgaggaagccccttgccaacggccactatactcagggcacactactgtgcgcgaggctctcgcacccaacacgtgcaaaacactgggcgacgccttacgagttgcggtagccaccattgctcaccggccggtccaggttgagctcgcatttctcgatttggcggctgggcttcgagaaaccggcaacaaggatgttgctctgacgacagttggctcgagtgcagttacatccaaattgtgtagcattctggagaaacatggtcaaaatatcactcttgccgagtgcgaaccagcccttgcggcatttggcaatgaccttgatagcgtctcccggcacgaaattgccgttgttggaatgtcggggcgcttccccgaaagtgactcgctagatgagctctggagtttgctttcctctgggaagacgacgcacagggaagtaccaccctctcgcttcaatgttgatgacttttatgacccatctcgccgcacgcataacgccctcctctctcgccatggatgcttcatccggaagccgggcgattttgaccatcgtcttttcaatatatcgccgcgggaggccgctcagatggatccggtgcagcgcatgtttctgatgacgacctatgaggccctggaaatggccggctattctcccaccgacgacggaaaagaccccatgcgcatcgccacctactttggccagacggtagatgactggaaaaccatcaacgaacagcagggtatcgacacgcattttctgccggccgtaaaccgcagttttgccccgggccgcattgcacactacttccggtgggccggcggcttctatagcatcgacacgggctgctcttccagtgccacagctctttgtctggctcgtgaggcccttgcttcgggcgagtgtgatgccgcggtggtgggtggtggaacgctcctcaacgcacccgagtggttcgccggtctcagtcaaggaggattcctatcgccaacgggtgcctgcaagaccttctcagatgctgccgatgggtactgcaggggcgagggtgttgccgtcgtcgtgctgaagcgcgtggccgacgccgtccgaaacaaggacaacatcctcgccgtcgtcgctggcgccgcccgcaactccaatgccggcgccggatccatgacttaccctggcgagcaagcacaagcggctctgtatcgccgcgtgctgcgccaggccggagtgcgtccgcaagatgtcagcgttgtcgagatgcacggcacggggacgcaggccggcgacaaggtcgagatgcaagccttgcagcacgtcttcgcatctcctgtccaggcccgacggcagcgaccgcttgttgtcggggccatcaaggccagtctcggtcacagtgaagctgcgtctggcgtggtgtcgttgatcaagtccatcctcatgttgcgccacaacacgatccctccacagcccggccagccgtttacgttgaaccctcacctgaggcctatcatgggtgctgatattcagctcgccaacggccagacatggacgcgcaacggcacaactcctcgctacgccctggtcaacaacttcgatgccgctggcggcaacgtgagcatgttgcttcatgacacgccatcctttgctgaacagccgcctccgactcggcccgatgagcgatcgcgccatgtcgtcgtcacgtctggccggaccgtgacatcacaaaaagccaacaaatcccttctacgggcgtacctggcaaaatacccagacgtctccttggctgacctggcgtacactacgaccgcccgccgcatgcatcacgttcaccgagaagcgtacgtggccaggtcaagtgaggaactgctgcgtcagttggatcagcggtccgcagacgacgaaataagctcagagccaacgccttcagtggtgtttgcctttacaggccagggcagccaatacatgggcatgggcggtgggctataccgtacatcaccaactttcagacgacttctcgactcgtatcagagcctctgcaacgcacaggggctcccctgtaatatactggatgtgatcctcgcgtcaggcgaagatgtaaactccgaatgggcagcccgtgacatgcaggtagccaccgttgcgctcgagattgcgcttgcccgatactgggaattgctaggcgtgcggcctacacttgtcatcggtcatagcttgggtgaatacgcagcgctctgcattgctggggtgttctctgtgggagatgccctggcaatggcgtacgaacgtgcagccctcatctttacgagatgtgagccggccgaagcgagcatgctggctgtcggactccccgcaagcaccgttcgctggcgcctgagggattcggccgccgtagctggatgtgaggttacttgtgttaatggaccttcgagcactgttgttggtggccctgtcgctgctgtcgaagctctgcaggtctatctcaactcagacaacgcaacagttgccaccagtcgcctgcgcgttccgttcgctttccatacctgtcagatgaatcctgtgctggatgacctcgagttggccgccaagagtgtgaagttcaacactcccgatcttccggtggcgtctagtcttctcggccgagttgtgcaaccgggcgaggacggcgtcttcaacgcacgatacctgcgccggcacactcgcgagcctgttgtctttcttgatgcggtgcgtgcctgcgaggaacaagggctcatccaggaccagtccctcatcattgagatcggccctcacccagcgtgcatcgggctgatatcgtctagccttcagaaggtgactgtcaggggctaccccagcctgcgccgtggacgtgacgactgggagtccatctcccaatgcgccgccgctgcatattgcacgcagctccccgtggcgtgggcagagttccacaaggatcacgtcgacagcctccgcatggtgaccgatcttcccacatatgcatttgataaacaggagttctggcattcgtacaagacgaaggcggtgcttgaaagccacgccagcaaagcatcccctgtttcaccacctgcgtcgtctcgattgtcaactacatgtctgcattctgtagaacagtaccgcagagacggctcccgacttctcgcgacatttggcgtcgacctgactgaccggcacctatcctcggctattggtggccatgttgtcgatggagtagccatctgtcctgccagtatcttcatggacatggcttacacagcggccgcgtaccttgataacaagagtcgcaatgcttcttcaagctcttcattgggaggatacgaactaaccaatttgagcatgctcactccattggtgctccgcgacgacgttggactgcctcgagtctttgtggacgcggtccttgacccgtcaactgacgccgtctctgttcgattcctatcccgcaaggatgaagtcgcttccccggttgagcatggttcctgcttgattcgtttgaaccagcccgaggctgccacaacccatacctggtctcgaatacgatcgctggtcaaggcacgcgtacgaagccttgatgagtcggcacgccccaagcaagttcatgcgatggataagtcactcttctacaagctattttcagaaatcgtcgactactccgtccccttccatgctgttgaggaggctactgtagcggcggactttcaagatgccgtcatgaccatccattgtgattcggccgcagaacttggcagctttacctgcaacccctttgccattgatgccctcgtccatatagctggcttccttctcaatgtcaacgtgtccaaacccaagaacgaggttcacattgctaatcacatcgggtcattgcgcgtgctcggtgaccttgaatcagatggacctttccgtgcctacacggccatacgagatcaagatgccaaaacaggtacaagcctgtgcgatgtgtacctcactaatgctcaggacaagctcgtggcgctctgcacggatatttgcttcaagaggctcgatcgcgatttcttcgccatactgacaggctcagctcgcgcggtgcctgcgaaaccacgcacaaaccagacgacgccgaggtgcaggtggcaagacaggagctcaagcagctcagacgacacgtcctcagaaacggatacgcccgacgcgtcgtcggtaagcagcctgtcagacacggtagatctctctgcagccctgctcgccattgttgcggagcattgcggcatgacagtgcacgagttgagaaggtccaaaggcgtgacattttcgcagtttggtgtcgattcgcagatgagtatcagcatactcgccgagttccagaggacaaccgacgttgagctgcctgctgcctttttcatcaacttcccgacgccggacgccgtaaataaggagctgggcaacgagcagctggaagagacaaaggtacaagaacgaaagccaagaagtccgcaccagctaccggcgaaggtcaggcgcggcacagagcgcagcccagaacccatgggctcgagcgcgcatctgttaagtctcgtcgccgaatcactgggcctcgaagctggtgatctcacatcttcgacgaagttccaggccattggcatggattcgatgctgagcatcagaatcacatcacagttctaccgagatactggaatcgagctgccagctgccttcttctctgagcacccgacagttgcggccgcatgtgccgaactggatgggccggtagagaccgcggccccggacgagcgattaaggaactcttcagctgtgagcacgttgcatattcccatcatccctactgtcagtgcggcgaatgtgagcgcgctggcgagacagcacaagattgacaatgctgtctcccgagcggtcttgatccaggggcaatcatgctcctgcgatgctccattgttcatgacgtgcgacggcccaggcacagtcgagacgtacatccacctgagcgctctccctaatggccgccgtatctatgctctcgagtcgccattcctcgagtccccagagacgtttgacctatcgattcaggaaatggcttccatatttatccgcaccatccgcaggttccaaccccacgggccatacctaatcggcggctggtcagcaggctccgtgtacgcctacgaagtcgcacatcgcctcaccacacagggcgagaccatcggggcgctcatcatcctcgacatgcgacccccctgccctagcccgatcagcattgtcaccgacgatgtctttgacaaggctggtgtttttgagggcatcaaccgtgcccgggacctccccaaggacctattactcaagaaaaaggcacatctaatgggcacgtgtcgcgccatgtctcgctataatgcgccagcttttgctctcaaccgccagccacgccaagttgcagttgtctgggcgaagctcggagtagagaacagcaaggatgcccctatcgccgccatgtgtcgccctggtgtcgacattggcaagaagccggaacaaatggggccagcagagttcgagcgctacttgagttcttggttctacggccgacgtgaacaatttggcactaatggatgggaagatctcctcggggataatatatccgttcatgctgtcgacgggggtaagcttatccacctgcttacatagtagggagagctctgttgctaatatacatacagatcatttctccatgatgttcccgccgttctccacggccgttggaaagattgtctctgatactgtagcgctaggggtgggtagaacatgattcgaagagcggtggacatggcatgtgttgtgagcatatgagcaagactctatcttcattggacataaccaatttgcctatattcttttacatttattttgaagcgttgcaagcaatatcgtatggttgtcattatcgctattacacaaagctcaacacagacctcacagagatccacgtggctgtgggctgtgatattgcttacacaacatga | |
| PKS2 | atgggacatccagggagcttgtacctgtttggggaccagacattcgacgcagaacctcatctttacaagctcctagaatcgaagaatagcaatgccatcttacgagactttctcgaaaaagcctacgatgctctccgcgtgcagattttccaactgccaaaacccatgcgcgacgaattgcccagattcacaagcatagaagatgtgctcctctcgaaacgctgcatggggagcagtggttgcgtgccgttggacatggcagtaacttgcatatatcaactgagttctttcattctcaggtaaagttcgtcacaaagaatactatccacttcagttttctaacatgggtgccgtatagtcaagcaggaaaagaataccccacatccgaagatgcctgtattctcggtctttgcactggcgccctggctgcggtggctgtcagctgcagccgatctctcacagaactgaccccaagggcggtcacggctgttgtaatcgcatttcgtgttggagtttgcgtctcacagatgagactgcgtcttgtatccccatctgagctttctcgaacatggtttatgatggtggctgggcagtccgcttctcaagcagtgcacaggttccgtgaagaaagttcgctgcctcttacagcaaaaccgtttatcagtgcatatacacccaatggcctggcagttagcggaccacctagctcactagacgagcttgtgtcgtctacacactttcgagggctcaagtacaaagcaaagcacatatatgcgccctaccacgcaccgcatctttactccatggcagatatcgaggacgtattggcgccgcttgatatggatcgagggcctcagtcactaaagaaaatcccccttctctctagtgctggcgattggatccaggaggcgaatcctggggccttgtttaaagaggcactggaacaaattctcctacagcctatacgctggaacaaaatcatggacgggttacgcacaggtccgggttccttgtgggaagcaaagacgtgcaagattgaggtattcggatcccgggctgaccagctcatctattccgccctgaaacagtcaccaacacattctcgcatgttgtcgtctcgatcatcaatacacaagaaaaccgaccttggcagcttggatgaatctggaatccaatccaggcccaagattgccatcatcggcatgtctggaagattccctggtggtgccaataacaatgagcagttctgggatctgcttcgggaaggactggacgtccacgaggaggtaccgcctctgcactggtccaaagcccatgtagatgcaaccggaagtcgtaaaaatactggcgcaacaccctacggctgctggttgagtgatccagcttcttttgacgctaggtttttcaatatctcccctcgcgaggcaccccagatcgatcccgcgcagagaattgcattgatgactgcatatgaagctctggaacaggcaggagttgtcccagatgctacgccatcgacaaggcgggatcgggttggtgtgttctacggtgttacgagcaatgactggatggaaacaaatagtgctcagaatatcgacacatactttataccggggggcaaccgcgccttcatccccggtcgtatcaactactttttcaagttcagtggccccagctatgccatcgatacagcttgttcctccagtcttgctgctatccatacagctgccaactctctctggcagcgagacatcgacacggctattgctggtggcactaatgtgctgaccaaccccgacttcactgctggactagaccgtggtcacttcctctcccgaactggtaattgcaagacattcgatgactctgcagatggctactgccgtggcgaaggagtaggcaccgtgatcctcaagcggctggaagatgctgtggccgagaatgatcccattttgggggtcatcctaggagcatatacgaaccactcggccgaggcggaaagcattacacggcctcacaccggtgctcaacgtgccatcttttccaaaattctcaatgcaagcgccgtcagcgccgaaaccgtcgggtatgtcgagatgcatggcacgggaacccaggccggcgacgctaccgagatggccagcgtgctcgaagttttcgctcctcaaaaccagccagctcgacaatctccagtgttcctcggctcagcaaaatgtaatatcggccatggtgaagctgcttccggtgtgtccagcctgataaaggtgctgttgatgatgaagaataacctcattccaccgcatgtcgggatcaagacgaaaatcaaccacaagtttccaacggatctgaaggagcgcaacgttcacatcgccagagaagctgtcccatggcaagcttctggctcaaaattgaggcgtgcatttgttaataacttttctgccgcgggcggcaactctgctttgctcatcgaggaagcgccatcgccaacacacggagatgaccaacaacatgttcccggcgcgaacctcgtcgccatttcagccaaaacgggcccctcccttcagggaaatctccggtctctacaggagtttctgcaaagaaacaaggataatatcaaagttggccagctctcatacaccacaacagcgcgtcgtgttcaccatccccaccgtgtcatgcttgctggctcttcggtcgatgagcttctgggtcagatcgacattgctgttcgcgatcaattgggcatgacacgacccaagaataagcagcccaaagtcatcttcgctttcacgggccaaggagctcagttccctggcatgggtcgacaaatgatggaaacgtatgccatgttccgcacagaactccgtcagttggaccacattgcccgcaatctcggcttcccatccattattcccgtgtacatggccagcgaggatcaggccttggacgagtttccgcctattcaagtccagcttgccagtgtctgcatgcaaattgccttggcacggctctgggcgtcatggggcatcatgccagaggccgtcgtcggccatagcctgggtgaatacgctgctctgaatgtcgcaggcgtcttgtccgactcagacgccatttatcttgtcggcactcgtgccaagcttctgcaggatctctgtacccgcgggacacactcgatgttggttgttcgggcatcggccgataccatttcgaccacacttaagaatacgccttaccagattgcatgcatgaactctcccattgagaccgtcttggcaggaccagatcaagaaatcactctggcgtctcaaattctcggtgatgctggtatcaagacgaccaagctaaagattccttacgccttccattcatctcaaatagacccgattttagcgcctttcgaaaaatctgctcattctgtgagatatctcaccccaaaatatcccgtcttgtgtccgctggatggaactcttgtcaccactgatggaacatttgggccagcttatctcggccgccattgtcgccaacctgtcaacctcctccaggctcttcaaacggctcgtcaaggaagtattatcaccgagcagactattcttctcgaggtcggaccccatcctgctgtttcaggcatggtaaaggccactcttggccaacaaatgaccgtgatttccgccctcaaccgcaagtcagccaaggacgtcctcgccgacgcattgaaggtgctctatcaagccggcgccgacattgcatggacagtctaccataacgacttcaaggcttctcagagggtcataaccgggctaccggcttacagttgggatttgaagccgtactggattcaatatgtgaatgactggtcgcttcgaaaaggtgatccaccacttgtggttaatgagactgagaagacgagggaaaagcttgaaagcaccactattcaccgcgttgtagaggagtcttcgtccaactgtggcaccaaacagcatattattgtcgaagcagacatagcacggtctgacttgaatcctcttgttcaggggcacatggttgatggcatccctctatgtactccctcggtgtatgccgacatggctctcacagttggcaggtatcttgtcttacagaagcagggctctggggtcaacaccagcaacaatcatgtggatgtatcagatatgactgtttttaaagcgctcattgcaaaaccgcaaggcccacaaccgcttcaggggcatattgaagtggactggaagagcaaccaagcagccatgaagtttgtcagcttcgatagcaagggaaaacctcaggagcacgccaagtgcaccattcgattcaccgaccgctctctcctgctggacaagctacaaaaagaatccgcatcgataaagacgagtatgcagcgcctcagacaaggcatcgcgcaagagacagctgcgcgtttcaaccggcccatggtctatcgcatgatccgcccgcttgcacaattccacgacgattaccgtgccattgatgaggttgtcctcgacagcaacactctggaggcttcggcctctgtgagcttcggagccgtcaaaaaaggtggccagtttgctctacacccagctatcattgatgcgtttacgcaatcatgtgggttcaccatgaactgcaacgaccacacagacctggacaaggaggtgtatatgaaccatgggtggggccgattccaagttttcgaggatatacattttgacagaccctatacttcgtacacgagaatggtcgaaggcaaggacaagttgtggtatggagatgtggtgatattcgatggcgagaccattgtggccgcctttcagcaaatcgacatccagtgcgttccgcggcgagtacttcaggttattctctctctggaaagtggcatcagtcccagcaagcgcggcgctgcaggcgctccagtctcgtcaaaatctcccaagacagcgtcgaccaccaagactgccattgccagcaacctcaacaaggccaccattccggaagtagaaactgcacgatcttcttcgagaatctcgacagccctacagatcatcgcggatgagagcggagttgccttggaagagctgactgacaacagcgctttcgccgacctgggcgtcgactctttacttggtttaacaatctccgcgcgttttagagaagaactggacttggatctggaattcgacgctctttgcatcacttaccctactattggtgagctgaagagctttattgcgccagcagcaatggataccggcaaaactgaagtgcagcatgaatcctcagcgctaatatccgacaaagcgtatgaaacggtagccttttcccacttaccaacaaatgacgtggcggtattgaaaagcaaaagtgatcataaccaaatcgacttcacgaatgtgctcaatatagtttcagaggaaagtggcatcgcgttggaggagttgacaggcgatacctacttcgccgatgctggcgtggattcactcttatcactcgtcattgtcagtcggttccgcgaggagctagacattgacatccccatggactcactctttgttgactgcgcgactgtcgatgacttgagatcgtttctactcaccacgccgtcctctggctcttctcgttccgacgagaacacagacggtaattcaatacccacgactggcaccgacaagatgtttgatacaaatgtccaagtacatgctattactctcacaccaccaacctcgggagcgtcatcagacactgaagactgcgaagagttacaaccattggtagttgtaccaagggcgtcttctctcgtccttcagggaaaccccaaaacagcatccaagaccctattccttttccctgatggagccggttctgcatcgtcctacgcgtccataccgaccatagcatccagcacttgcgtcatagctctcaattctcccttcctcaaagaagcggacaaactacgcgcggctttcctcgatgatattgtcatagatggttatctcgcggaaattcgtcgccgccaaccttttggaccgtaccacctcggcggctggtctgccggtggtatcctcgcttaccgcgcagcgcagattctgatcaagagtcaagaacaagtatccagcctcaccttgattgactcgccccctcctctcaaagggctcgatcgcctcccgcagcatttctacgattactgcgaaagcctgctcttgtttggacacaagtcctcgtccccgttgagtaccggcaagcccccagagtggctcatcccgcacttcaatgctacgattgatcttctccacgagtatcgcgcggaaccgatgcaaaacaaaggcctgacaagtatccccaaagtgaacatcgtctgggcagcggcttgtattgctgataacccgcgtcttccgaaattaccgccgcatccggacgataccggagggatgaagtttctcctggagcggcgtacagactttggtgggaatggctgggagaccttgtttcccggtagtgagagcagcattactattaacaaggtagatggggcgcaccacttctcaatgatggttagttcttccctggctgattagccatttcgccaatttgtttgtctaagatgacgcctacatcgaatatcttgctaactgctgcgtcttcatatagcgtgaaccgtactctcagaagctggcagagttccttcgccaggctatggcttag | |
| PKS4 | atggtcaccgccgcagacgtcggccatagggagccggaaatcgtcgcgtttggtgatctagcaagctctgaatgcattgttcccctccaaaacctactccgttcgaaaagcaatgccgacctcaccgatttctttgcccgagttggctttggacttcgacagtatctgggaagtctgtcatcgtcaactcaagagcttttcccaaacttcacaactctcaatgacctcgtctacagatgggaagaagctaccggtcgaccagcattgcacgtctttcttttgagtgttttgcaaagtgcccagtttatttggttagtcccgctacttccagacatagtagctggttgcaccactcgcctactcctccaattgtatcaaataatattctaacttagctttgcagtcactacagtgggaaacaagccagagagtttccttcagcagctagtacatgcatcattagcagttgtgtgggagagtttgttgccgccgctgccagttgttccaagtcaacatccagctttgttcccttggctgtagaggcggcactcaccgcgttcaggacgggattgaagtcgtacctaattggcagcgcaatcgcagccggaagtaaagatgtgatagagacatggtctgtatctgtgtcgccgactacggatgccagcgttgaagaaatgcttagcatgttcgcggaatccagaggggtatgcttatctgaagttcaccaattgcgcccatggtcactaacaagaccaacataggaaactgaaagttgcaggtcaatctgggtcagtgcctcaacaccaacaggagccacgtctcttggcggaaagccgtcagtcttatcgagctttgtcaatgccaacaaagacaagctcagagtcaaaccattgaacgttacctcaccatttcatgccccccatctattttcagagcgagatgtcgatgaaattatgagagacgttgctgcagatgtcgggaaagacgcaatgcacaggatcacgttgatatcgtcccagacggcatcagaggcggaaacagatttgactttcatcggacgccttcgaatggcagtacatggcgtcttaagggaccaattggaccttaacctaatatttgaatcatgcggcacgtatctcacccagctgggaagccgcagatgtcgattgacaacattcacatacggtgcgacaccgatccttgcagcaacattggccaatgccttcacaatacaactggatatagaagacgcctctcaatccagtgttgatgagtctctccccaccgctcagttttcggaatctaagatcgcaattgtcggctattctgggcgcttcccttcagctgaatcaaatgaggcattctgggaattacttcgcgccggtcgtgatgtacaccgcgaggtgcccagtgatcgttttgactggcggacacattatgatgcagacgggaaaataccaaacacaagccgcgtcaagaacgggtgttgggtggacaatcctggtctttttgatgcccggttcttcagcatgtcgcctcgggaggccgagaatacagatcctgctcagagacttgccatcttgactacatacgaagcaatggagatggctgggtttgtcccaaacagaacccccagcagccaagccgaccgggttggcgtttttctcggatcgacaagcgatgactggcgtgaaataaatgcgtcgcaaaacgttgacacctacttcataccgggtggaaatcgagcctttggaccaggacgcattagctattacttccggttatcaggtccaagcatcagcgttgacacggcatgctcctcgagcttcgttgccatccaagcagcctgtagctatctacgcagtggacagtgtgacaccgcaattgctggcggtacaaacatcttgaccaacccaaacatcttctccgggctagacagagctcacttcttgtcgactacaggcaactgtaacgcgtttgacgacgaggcggatgggtactgcagagctgacgcggttggtactgtgatgctgaagcgattggaggatgcagaggctgacaacgatcccatattcggtgtcattgtggaaacaaacataaatcattgcgggcacactgagagcatcactcgaccacacgagggagatcaagccgctctcttccaacaaatcctgcggcaagccaactacaaccctttggatgttagtgtcatcgaaatgcacgggactggaacacaagccggcgacgcgacagagatgaactcgatcctatcaacctttgtgccgcagagagaacgcaccaaagccgagccaactagaccactttacattggttcagccaaggccagcgttggccattctgaagctgcctctggtgttacctccttgatcaaggtgctgcttatgctcaagcacaacgaaataccaccacactgtgggatcaagacaaagattaaccgaagctaccccctagatctgacggaaagaggcgtacacattgccatgaaacctactccctggctgcggcattcaaaagagacaaagcgcgctgcctttttgaacaatttcagtgctgctggtggcaacactgcaatgctgttggaagaagccccacgaaaactgagaaatatcgataacccgagtggcttgcatagtcatctggttgccgtcactgcaaaaacgcccaagtcgctggttggcaatgcacactcgcttgtggcatggctgcgaaagaacggtgatgccgcgcttgcagacctctcttatactactaccgcacggaggatgcagcatgactatcgaatgatggtatcggcccagagtattccctcactaatacaaagcctagagtcactttcctaccaaaagccgcagtctctcaaggcgatcctgcctccgaccaaacgacccaagctggtattcatcttcaccgggcaaggaagcttgtacatcggcatgggcaaaaatctgtaccagacttacaactctttttgtcaggatatccttcaactcaaccacctcgctgagagctgtgggtttctttccttcattggactaattgacggttcaacgagcgttgatgtccacactgtcagcgcagtcacatctcaactagccttgctatgtgttcaaatagcgctggtaaagctcctgggtaggtggcgaatccgtcccggtgctgtcatcggccacagccttggtgaatatgctgcattatatgcggctggcgtcatcagcgccggagatgcgatttatttagtgggaaagcgtgccacattgttggagcaacactgcaagaaggggacccatggaatgcttgttgttaaaggttcgcaacaagaaacagagcgcctgcttcagtcggtcgaaaacgactttgaagtagcttgtgcgaaccaccccactgcacatgttgtggccggtccgaaagatcaaattcctgccatcgtcgccgaagctcgcaaagcaggcttgtctacagtcgaattgaatgtgcccttcgccttccacacgaaccagatcaacccgattttgtcggagtttgacatggcggctgcgcaagcagggctcgtgttcaacccaccgaaaataccggtcatttcgcctctgctgaaaagagttgtctgccaaggcgaggagaacacgctcaatatgtcatatcttgttcgagctagtcgtggtatggtcgacttttctacagccttgaacacggcgcaacgggatggcctgttcggtgctagggcaatctggctcgaagtaggagctcaccctctttgcggcactatggtcaaacagactctcgggcctgaagaggaggttttgatcacattgaaagaaaaggtggaattgtccaagactttactcaaatctgtggaggccttgtatctcgccggctgggatatagactggaatgagtatcatcgcgagctgcccgtcacaccccaagttctggaacttcctcattacgcatgggatttgaagaactactggatcgactacaaaaatgacttttgcgtcaacagaaatggtcatctcacaaagcccgatgcgattgaagcgccaataaggtacaagtatatttctcccgcggcgcagaaggtgattgaagaggttcatgagaagagcgagtcttccatgacggtggaatccgacatttttgaccccgacttgctacctgtcttacacggccatgctgtcaatggcgcatttctctgtccatctgtaagatgttcacccccatcttcatgatgcttgattactgatatgtttctttcatagtcgctgtatgccgagattggcctcacaattggcgcttacctattaagtgagcaaaagatgtgtcgggagactacaggtctggaggtccgcgatctccatattgaccagcctctcatcgccaagcctgatgaaaagacacactgctttcgagtccaagttcatgcggattggacctcaaacaacattacccttgccattttcagcgtcgatacctctggcagcaaaacggtttcccacgctacgctgaatgtttgtgttgcacctcaccagcggtggctcgcagattggaagcgcaacacctatctcgtcacatccagattgaaagcactcgcttcggacgaaaattcacaaaagctcagacgacgcatggtatacaagctatttgcctcactggtagattatgatcaagcgttcaagggtatgtccgaggttcttttgaacagcgacgagttggaggcggtgtcaacggtcaatttccaggtttctgactcacggggcgaattcggcatcgatgctagatggatcgatagcttgggtcaagtttcggggttcatcatgaatgcgaatgacaccatgaacaatcaagagaaagtattcatcaaccatggatgggaaaaattgcggtatgcagagaaactcgatgcgtcgaaaacgtaccaagcatattgtcgcatgcagcttgccgagaagacgacctacattggtgatgtctttgttcttgacgacgatcgaatcgtcgctgtttacgaaggcgtgaaggtgagccaagcctgtgacatgctgcttcgatacctgttacttacctacctacttagtttattggcatgggccgtcaggtactcaaccatgtcttgcctcccaaaaggtctgacactggcaaaacgcctattgtaccaacaccagtcaggcgtgtcgcaagcctctccaatctgaatgagaatgtcaggcagagtcgttctcttgaacgggtccgacgcaccgaaagcaagatgcctgacgctcacatgggtgttgatcgtatgacggagatcatgtgccaggagattggtgttccggcgtccgatcttgagccagagtcgcaactcgccgacctgggtattgactcgttactgtccctgactatcacttctcgcatccgggaagaacttggaatggaggttctctcaacagctcttttggaatgtcaatcagtaggagaactgcaaagtctttttgggtgtagtgagagtttcatctctgcaacgacgactgattttgacagtttgtcgaacagcagcggggaatctggggatcacgaaagttctctctcgactcccagtgagatttgtggttctgaatggtcagaagatatgcctgttcgccgcacattgtgggagacgatcgctggcgagacggccacgtccatcgaggaactcacaccttctactaccattgccgacattggcattgattcgcttctcgccatcacaatgtcggtaagcctgtgcgagaaattcagcgcccacatcccgagcaccctaatcctgggctgcgagactcttcttgacatagagactgccttgtgcaaggtcctgggcattgtgcagggctcctgcgccgccagcccaagaaatcgtactgatttgcgtaatgttcctcgaaagccctcctccacggctggcgatttggggatttcggataacccaccagctacctcggcgcttctgagcgggtcaccaaggacggcaaagtccatcttgatcatgtttccagatggatcgggatcagccgcttcatacgctactttggctcctcagttaccaagagacaccgcattgtacggtctcaattgcccttggcgcaagaatggtctagaattgacccgttccagtatcactgtgcctcaaatggtagccagccagatcgtcgaggtgcgtcgcatcatagaatcccacaagcagcacggctggaggaacaacaccagtggcactccaaatttgatactgggcggttggtccgcaggaggtatcctcgcgtacgaagccgttcagcaatttgctgccgagggcattactattcgaaaattggtgttactcgactccccggaccctgtcaatatgagagcgccgccccgcaagatgtcttattttctcgaagcattgagcaagtccggtggacgtctggggaaagcgccagactgggttctcgaccactttgacggcatgatgggggtcttgggcgggtatcggccggcacctcttcccgagagccttgtgctagatacgctgctggtgtatgcacgaaagggtgcctggaacgagtacgacgaaccgaatacgtgccttcgggcgaacgaggatcatgatgtgagctggatcctccgggatcgaactgacttcacggcgggtggctggaagcgtttgttggggacacgacaactgtctgtcaagattgttgatgacgctgatcacttcagtctgatggaagatgagagaaggacgaaacgggttgggaatttcattattgcctttttatag | |
| PKS5 | atggccaagatattttgccacgtctttggggactgccacggtgaaaaatatgatctattgaacaaatatttcagtaagcagcaatctggcattctggcgacagccttgatagaccaagcagccatttccctgcaaagcgaagtcatatcatcgaaatatcctcagcaagcacctatctcccccttcaatactattaaacagctcaacgagcagcaccatgcatccaacgttaagcacttgggcatcgacaacgcgctgaattgtattctacagctctgccactacgtcgagtacgtcggattcgttcgtaattttaagttctagctaacgtgcatgcacagctatgttgaaaagtccgaacaggcgttctgtgaccctcatcagagtatattccttggaggtggcgtcggtctcctatgcgctgctctcatagcgtcaagcccaactccagctactttgatctcgaatgcacttcaagccatcttgctttcttttcgtctaggctcctatgtcgggtctgttgcggaaaggatctcgaacccgaatcaacgctccaatccggtgcaagagtgggcgtacaactttcctggcctcgatttggaatcagccgaactctatttgaacgagttcaatgagtccaatgtatgttcgtgagagcttgttccagcatgtgtcgtgctaacaggtagtaaatagactatttcaaaagtacgtcgagcgtacattagcggcatatcacgaggccatgtacagatatcagcaccacccagtattcttcagaagatcattgaccaaggggttttcacatgtaatccgattcgttgctccgtctacgggccatatcgggcgccccatttgcattcgtcaattgaagtggacgatattctgagcgcagctgacagtaatttacaagaacgggcaaacaaatgccggctcaagtcacccaccgtatcatctggagagacatggctcagtgggtgcgaaacaactcttgtcgtgcacgcagccacacaccagtatctaagccagcgctttttggcgtcgaaattagcacgcaactgtgcttctgcgcttacagcctccaagagcaagcattgcgctgttatagctcacggatgccatgaactcgcggaagaattgatcgtcaccctaactggtctgactagcatcggtttcacacatgagagatcttgtcaaacaacagagagaattgctggggaaaatagcccttgcgagttgaactggaactcaaagttagccatagtcggaatgggaggacgatttcctgatgcagacaataccgacgaactctgggacctgctctgcgatggtgatgacgtccacagaatcataccggaagacagatttcccgtcgaatcgcatgttgacccagagggcaaagcaactaatacttcctacacaccatacgggtgttgggtaaagtctcctggccttttcgaccacaagttcttcaacatgtctccaagagaggccttgcagaccgaccccatgcagcgactggcaattgtaaccgcttatgaggcgcttgaaatgtctgggtacactcctaatcgcactccatcaacaagactggatcgaatcgggacattttatggccaaacgtctgacgactggcgcgaaatcaacgccgcacaggagattgacacgtattatatcaccggaggaataagggcctttggacccggcaggattaactacttctttaaattcaaaggacccagttacaacattgataccgcgtgctcgtccagtgccgctgcgctacaggtggcatgcacctccttgctggcaaaagagtgcgatacagccgtggttggagggctgtccatcctcacaaatcccgacctcttctgcggcttgagccggggtcagtttctgtccaaaaaagggtcttgtgccacttttgatgatgcggctgacggttattgtcgagctgatgcctgtgcttctctcgtggtgaagcgactggaagatgccatctcggacaaggataaggttctggctgtcattctggggacggcgactaatcactcggctgattcaatatccatcacccatccacatggtccaacgcaatcttctctgtactctgccattcttgacaaggctggagtcgaccctattgatgttgactacatcgagatgcatggcactggcacgcaggctggagacagtacggaaatggcctcggttctcaacgtgttcgcgcctgagaataggagaagagatctagacagacccctctatctgggcacagtaaaaccgaacgtcggccatggtggagcggcatctggagtcacatcagtgatcaagctgctgaagatgctgcaacacgacacgataccacctcatgtcggcataaagacaggttcagtactcaacagagcatttcccaaagaccttgcagccagaaacgtcaacattgtgtttaaaaacaccccattcaagcgccgtgacggaaaaccacgtctagcgttcgtgaataactttagtgctgctggtgggaacacagctttgttgctggaagatgcccctccccagctgccgccagtcccggaccctcgtgacgcccatattatatcagttacaggaaagtcaaagtcggcgctgatgaggaacgcagagaaactggctgggtggatggctatgcatcccgaaatcccgatttctgacgtcgcgtacacaaccactgcaagacgcattcagtatgactggcgaataagtgtcgctgcttcgacactccaagaggcccaggcagcgattgaaacaagtattcgagatgataaggactgtcatgttggaagctcaaagccacctctttgttttgtattcacaggccagggttcctcgtataaggggatgggtcaagagtactacaaatcctactcctgcttccgtaagtggatttgcgacttcgagcgcattgcacatatgcatggatttccatcattcatgtctctcatcaattgcgacgatagcgagtcttgcgacacgtcgcccatcgccgtgcaactagcaattttgtgcttcgaaatggccgcactggaactctggctgtcctggggagtcaagccagccgctgttcttggccactcattgggcgagtatgcagcattatatgcctctggtgtcttgtcggcaagtgatacgatatatcttgttggcgagcgagccaagttggtcgtgcaacactgcagtcgtgatagccatgccatgatggcagtcaatgcacccgtcgccggtgttttgccaattctggatcagtacaacgttcaaatagcctgtttgaacagcccaaatgagactgtcctctccggaagctccattgacatatcacgggccgccgaacaattgaagacggatggtctatgttgcacgctgctgcgcgtgccctttgctttccacaccagccagatggatccaatactggacaagctggacagacttgctcataatgtgagcttttcaaacccagaggttcccttcgtatcatcactattgggtaggcctgttgatgagattggtccaacatatattcgagatcatgctcgcggagtagtgagattctccgatgcagtctgttcggcgcaaaagctggcttcaccggacagcgcgatgctctggctcgaagtcgggccgcactccagttgtaccaacatgatccggaccatcattggcactagcgcaattgcaataccaacagcaagacgaggagaatcgatgcacaaggtcctttgtcacagtatcagttgtttgcatggctgtggtgtagacttggactggaacgagtttcaccgagacttttcaaagtcaactacgctcgtggacctcccatcgtattcgtttgagaagaagaaccattggattccgtacgaaggcaattggacattgtcaagaagaaagggctcaaccagtgccgccctacttcaaaatagaaagctatcaactacatcggttcaccagatagtcaggcaggttgtcctggcaggcgttgccatgatagatacggaaacaaacctgtctcgggatgacatccggcccaccatcacaggccatcgttgcaatggagtggcgctttgtcccgcctcactctacgcagaaattgcaatggccgtctccgaatatgcatacaagctcatctgtccaggccatgggccacctggcttgaatgtggcggacctgagtatccaaaaggctcttttgttcgacgacaaggccgatgcccatattcttcattgtaccgttcagactagtggcactggtgaccatgcaagtgtcgtatttcatacgcgcaatgggttgctcaagaatgaacatgcacgctgtacggtatattacggagacttacgtgtgtgggctgccgagttggcaagagtcaactacctcgtcgaggcacgtattgatgcattagcatcaggtaacgctagtcatgcaatctcaaaatacaatcaacctgcggcctaccagagcttcgcgagtttcgtcgactatggtccccaataccaggggatgaaggaggtgttgttgtgtgctaaatcaggtgaagcgactgcagaactcagtttccaagcaagtaatgaggggaactttttcttcaatccgtattggattgatggctgctgtcatctctcgggattcatccttaatgccaaagaagccgcagattcgtccgaggaggtattcatctcagagggctgggagtctctgaggttcattgagcgtctccgtgagggaactacatatcggtcatacacacgcatgcaaccagtacatgggatgagtttggtcgcaggtgatgtgcacatccttagagacaaatgcatcattggcgtagcaaatggggtgaagttccgttctgttcccaagagggcgcttcatctactactgccaccaaccattacatgtcgcacaagcaactcccagcgtcagtcaattgcagaattggctctacaaactgacaatgtcaacccaaaaattacaaagaaagggcagggttacgcgacattcccagcgctgtatcgaactacagcacagacctctttgatggatgagtttcttcgaattgtacaagttgaaattggttgcgagactgaagacttgggtgacaatgctacctttacatcccttggctgtgactcactgatggtgttggttattgctagcagagtgcatgaggagttggagataaatattcgaccccaggcatttctggagtaccctaccattggggatttcaaagaatttctagctagtctgcgtatcacggacactagaagttcggatgcctctacttcgtctacggtatctggtgttaccgacgagccagatatcggacaattgtcggattccgagagctctacatctcgattaggtgacaagacctgcgaagagcaggaagaaacaaaaaagtctctggatactgcaatggcagacgctccgatcagaacaatcgagagggttgaatgcctcagggatcagtttcccaaccgcaaagccaaatcagttctgcttcaaggaagccgtcagatagcgtttaaaaacctgttcatgattccagatggcagtggctccgcgtattcctacagcgagcttggttcacttggttcagagtgggctatttggggattggtctccccgttcatgaagactcctcacgagtacacctgcggagttgaaggaatagcacgaaagtttcttgaggaaatgaagcaacgccaaccagttggcccatatgttttagcaggttggtccattggcggaactattgcttacgagataacgagacagttaactcggtccgatgaacaagtctcccacttgattctgatcgacgcaccatgccccctccttgacgatccattacccagacatttgggccgctggtttgctgatattggaatcatcggctcgaagatgggcgcgagcctctgtattccagactggattcttccacattttgatatgagcgccaaggcattcgcggattataagccggtacctatctctcgagaccactgtccgatcaccatggctgtttggtgtgaggatggcgtatgtcgaacgcagcaagatccacgcccgcatccatacccgactggccacgctctctttctgctggagaacagagaagactttggtccaaaccgctgggacgagctcctggcttcggaaaagatacagacaaaacgcatgcgcggaaatcattttacaatgatgcgtgaacccttggtatgcttcctgcttcaactgggtacaggtggaaactaattggtctgattagacacaggatttaactgcaattatttatcaggcggtcatgtctgataagccctaa | |
| PKS6 | atgtatcgatctgagacaacagatcggcggacgctcgtcgtgttcggcgaccaaaccagcaatgtaaccagcatcgccaagcaactcttcctgggcgttccccagagcgcagcagccacagagttccttcgcgatgcgagtgcggccctcatggctcagagagatcgtttacggccgtttcagcgcaaccatcttcctgatttcaagaacctccatgacatagcaaggatatacgacgaggccagcggcgtatgccatccatccatcaccagcgtcatgctgtgtgctacacagttgttgcaattgttcaggtcagtaatgttcacagtagcgccagagcatagcgttgcgtatcagcgtctgacgattcacagatatttcgaagaacatcgcccggggcccaacgagaaccatcacgtcactactgttgggttatgcaccggctcccttgccgccgcggctttcgcggcctctaacggggtcgaagatctcaggtggttggccgtcccgactatatccatggcctttcagctcggcctacaagctgctacggcgagcagcatgctctacgaacaagacgccgcgagactgggcagctggtccgtcgcaatacccaacatgcccgaggatgaggctatatctgccttgtccgtgtacgacgccggccatcatctatcgctgcgtcacagatgcttcgtcagtgccgtcggcctgaaaagtgtcaccatctcaggagctccgcccgcgttgagggccttcaccgacacccttgccgcgtcgcagcctgagcgaaggctcgttgagctacccgtgtttgcagcattccacgctgtccacattcacccatttctagacttccctagcttcctatccaggtgtggtgtcagccttcccctactggcccgcttcaaacccgtacaccgtctcttgtcgccgttgacgggcgagcctgtcgtagccaacgacgcctttgggctgttcaagacagctgtttgcgagacattgcaggcacccttgagactagatcttatagtcgaagggttcgccaagttcatccaggacgaaaagatctcaagcatctctgtcgaagttgtgtctcccacaacggtggcagatggtattgtggctgccctggtacaacaaactcaagccagcgtttcatcgagaccaatcgtggctctgccatccgctggtgagatccggagcaatccccctttcaacaatgtacctctggcaatcgttggcatgtctggacggtttccagacgccgacagtgccgaggcgctctgggaccttttggcccagaaaatcgacgcccatcgtgttgttcccaaggaccgcttcgatgtaaatgtacatgttgatcctgctggcaaggctaaaaacacctcctggacgccttatggctgttttattaaccgtcccggcgagtttgaccctcgattcttcaacatgagcccaaaagaagcgcttcaaacagaccctatgcaacgactagcgctcgtgacggcatatgaagctttggagcaggctggctttgtgccagatcgtacccggtcaagtgcgtcgcaccgggttggaactttctacggccagacgagcgacgactaccgagacgtcaattccgcgcaggacattggaacgtactttatcaccggtggcattcgagcctttggccctgtaagtcgaccctggctgctcctattcctggtcctagactcaaaagcctaacgtgcggtagggccgaataaactactttttcaagttctctgggcccagctactccgtcgacacggcgtgctcctcgagcctcgctgccatccagctcgcttgcactgccatctggaatggtgagtgcgataccgcagtcgccggagggctcagtatcttgacatcgccggacctcttctccggcttgagccggggccagtttctttccaagactggctcatgcaagacttttgacaacgccgcagacggatactgcagagcagacggtataggaacggtagtcatcaaacgactaaaagacgccgagagcgatcgtgacaatgtgctagctgcggtattaggcgctggcacaaaccattcggcacacgccatctccatcacccatcctcacgccgaaacccaatctgatctctatcgccaggttttgcagcaggccggcgtggatcctcacgatgtttcctacattgaaatgcatgggaccggaacgcaggctggggacggaacggaaatgaagtccgtgagcgatgtgtttgcaccgccgtcatctaaagcccgtgccagcgataatcccctctacgttggggctatcaaatccagcgtgggccacggtgaagcttcttccggagtcatatcgttgatcaaagccatgaccatcctgaagaagtctctgataccaccccaggccatcaagggcgagttgaacgtcggcttcccggatctgaaggcgcgaaatatccgcattgctaggcacatgacaccctttccagagaggaaggggatcaagaagacagtgctggtcaacaacttcagtgccgcaggtggcaatacagcactcttgatacaagagcctccagagacccggatcgagcacaacaaggaacctcgaaacgcccatccagtgaccgtctcggggcacactcattcagccatgctcaacaaccttgatcggctgatcgagttcctggctaataactcggagacatctccgctcgatttgtcctacaccactactgctcgtcgaaggcaccacaacttccgtgccaccgtcgtcgagagctcgactcctcggattctcgctgccctgaagctcaagagggcgtcgctagcggaaggcacatttatcccgccaaagaaaccacggtccgtcgtgttcacgttcacggggcaaggatctgcatatccagccctggcacgcgagatttacaacacgtccacgcagttcagggctgatgtttggcattttgacggcatcgcccgtcaacaaggattccccaccttccttccgctcatcactgacgccagccttgccgacatcaatgtcctgtctccggctcagacccagattggtcttgtctgcatacaagtcgcgctcgcacgcctctggagctcctttggtatcaagcccagcgccgtcatcggacacagcctcggagaatacgcagcgttgcaagtcgcgggtgtcttgtcggtgagcgatatgattctgcttgtcggctcgagagcgcgtctcatggagagtaaatgcgaaaggaactctcacgcaatgcttgcagtcgccgcccctgggtgttccgtccagtcaatggcagcagagtgtctggatggcgtcgacctggcttgttccaacacgccccgagaatccgtcttcgcaggagaaaagtccaagattgagcagcttgcgcaaaagctgaatgagtgcaagatccgctctactcagctacgcgtgccttatgcctttcactccacccaagtcgacagcatcttggaggactttggcaatattgccagcgtcgtcaacatgggcgtcccgcatacaacagtcataagcccgacgcgcggtagggttttggatgcgggagacaagatagacggtgagtacctgaagcatcactgccgccacacggtacaatttgcccaggcgatagagcacagccgccaagccggcgtcatcaccgatgcgacgacattgatcgaggtcggaccccatccgatctgtagtggcatggtgagatctacgctgggcgagaaaacgcagactctgtcgacactgaaccggaaagagaacccgtggaatgccattgtaggcagcctggcttcgctgcatgacgccggccatactgtgaactggtccgaataccagcgagatttcgagtctgcttgtcgtttgatacacctcccctcttatgctttcgacaacaagaattattggatcgactatcggaacaactggacgctgcgcaagggcgacccttccacggctgcagcagacgcgcagagcgtcgtcgcggaccggagcggcaacgcgaagccatacatctcaagctccgtccatcgcgtggtggaagaggattacaacagcgctcaaccgagggtcgtgtttgagactgacctgtcacacccagacatgcacacggccatgtctggacaccgcgtgaatggctctgcactttgcccttcttctctgtatgccgacattgccttgaccattgcaaagtacattcgggaccaaccaggctcaggactggatcaagatgggcatgacgtctgcgacatggaggtgacgcaacctctgattatcaatcctcaccgcgaccaagagaacagaacgttgcgcgtcgtgacacatgtcgataaaagaagccacatcctgtctttggagtattcctcttacggaccagcaccagcactcaaaactccagtcaagcacgccatgtgccgcgtcgagtttggtaatcggcaaaaatggtcgagacgatgggttcacgacttacacctggtcaaggaccgcattgcgggcttgaaggtcgcgacccagtccggcaaagcgagcagcatcacgaagcggcttgcctaccgtctattcgcaaaccttgtcgactatgctccagacttccagcgcatgggtcaagtctggctagatggcgtgggccgcgaagcaaccgccgtcgtctccctcgacagacgagagacggatgccaactttaccttcagcccttaccacctagacggcttactccacctgtcgggcttcatcatgaacggcaacgatgatattgatgccaacgaagcagtgtacatctctcacggctggcaaagtcttcgcttggcagagcctctcgtgccagacaccaactaccatgtctatgtcaagatgcttcccgtcgacaagaccatggttgccggaaacgtctatgtcttgcacgacgacaaggttgttgcattgtgcgaaagcatccgcttccagcgtgtccctcgcgcggtactggacatgctgctccctccagttccgacgagtcgtcctcgtcgagctgcaggccctgccccggagagatttaaacatggaggcagtccaccagtcgtgaaagtcgctgagcgggtggctccaacaaagaagcagttgccgttggctccagcacaactcccggccacatcagggactgtttcgacaccacagtccgagctcttcatgcaggtcatcgccaacgaaatcggactcggcacaggtgaactgaacgtcacagacaacctggtcgacctgggtgtggactccctgatgtcgctggctttggctggacagctagttgagcagttcggagtgaatgttgatcatggcgaattgatgcagtgtgccagcatcaaagacctgctcaggcttctccaagataaacaggagccgccgccggcttccacccagaacgaagaacacgctggcaccagcagcagcagcagcggaagcagtgcgtcttcctcggccggcggcttgggtaccgtgacgccccccaccagagccacaactcctgctgtgaaaccggccgacgacatggggcgtctcgtatactccatcgtcctggaagagacgggcatggaaggagaggacttccagtcttctgccgagctgagtgatctcggggtcgactccctcatgagcctcgctatccttgccaagcttcgcgaagcaggcgtcgatctgagtccaacctttttccttgacaaccgtacaatggacgacgtctttcgtacgctcggcaaaggtaaggaccctggcgcagaagaagaacagcaagtgattgacagcaccgcatcgcaggcatccaacctccgctctgaagccgctgaagccactgcagccgctgccgccggctcccgaccccatgcgcagtgtatcctactgcaaaaggcaaccgcatctccggctgggcaatcattgttcctcttcccagatggctcgggctcaccgtttgcctacgcagctctggaaagactagatccaacgctcgacgtctatgggctcgcctgcccgtttttgaaaagcccgtcagccttcaaagaccatggtattgaagcaacggcggcagagtatatcaagacacttctggccaagtgtcccgcaggctcactccatctcggcggctggtccgtcggtggcgttctcgcgttcgaggcagcaaagcaactgaccgaggtccacggccgccaagtagccagcctggtattgatcgatgcgccctgtccactgaccttgccgcctatgaacgggaggctgattaagttcttggactctcttcaattatttgctcctcctggcgagcggaatgcacgatcccgagcggatacggaaaagcaccgcatggttttggagcacttcgatgcgactgttgaatgcctcggccgctacaggccggcatcgatggcaatggcaatccggacattggtaatatgggctagtgatggtgttgttggacacggggatgctatcccggaccgcatgttgttggatgatcccatggcaaactggatactccaggatcgagggactcccggccctcacggatgggacaagctcctgccatctcacaacttggagatcgtgacaacgcccggtaatcacttttcgatgatggtgggtggcaatgttcgggcactctcgttgggtttgcaggcgcattttcaaggtttaaatgccaataatgtaggcaagtga | |

Table S. 6: Predicted protein sequences of *P. marquandii* nrPKS and accessory proteins.

| **Gene** | | **Predicted protein sequence** |
| --- | --- | --- |
| *stpA* | MYDTVGTAMADGTPARLVYFGNEFPNDDLSDIFRKLHQHSKDRRFRLLSAFLDEVILVLQQEFAKLPHHVRSQVPHFDNIVTLSEMGFLRELGLGAAMESAFLLTLQVGLFIGHHEAKDQELNLPKGPTMLAGLGVGLFTGASVALSTSLAEVVKNAAELLRVSFRLGVYIGDFSSKLESPQPDGSLQSWSHVITGMTEESVSSEASRLNEELGSHAISKLFISAADKTSVSLSGPPSRIKAAFQHSSELRYSKSIPLPVYDGLCHAKHVYGQRDYDAVMDSENSLVPMSRKLQLPLISPRTGKPFLATEAGDLMKEIASELMTSAIYLDKVTDGILGHVDSVSTADELYLATFRTSIAFKGILQTLENTFPDVKILKSDMADWVHEDFGDRRPGNVGCSKLAIVGMACRMPGGGNDLDQFWELMEQGRDVHTTVPLDRFDLATHYDPSGNTGNAATTPFGNFIDRPGFFDAAFFNMSPREAEQTDPMHRLAIATAYEAMEMAGMVTGRTASTRRERIGTYYGQASDDWRELNGAQNIGTYAVPGGVRGFIAGRINYFFKFSGPSFCVDTACSSSMAAIQLACTALWAGEIDTAVAGGVNIITDPDNYCGLGNAHFLSKTGQCKVWDKDADGYCRADAIGSVVLKRLEDAEADNDNILGVVTAAATNHCADAISITHPHAGHQKENYRRVLHNAGVNPLDVSFIEMHGTGTQAGDAMESESVLDVFAPLRPRRRADQKLLLGAVKSNIGHSEAAAGVSSLIKMLLCFEKSLIPPHVGIKTEINPRIPKDLDRRNANMAMELTPWVRPAGKKRIAMVNSFGAHGGNTTLLLEDPSERDRPRLSLESADGRALYPIVISAKSKKSLQANIEGLLGYLEKNPGLDLADVSYTTCARRTHYNLRVATSASTVSGLQKFLRNAIDNKVGLETKAIPPNIPSVVLTFTGQGASYKGIRQDLFDEVPFFRDQVLQLDQLVQRLGFPSVVPAITGSDDDEVQSPVISQLSIVVLEIALARFWSYMGVKPSAVIGHSLGEYAALAVAGVLSASDVLYLVGRRAQITQELCTPYSRAMLSVLGDLDDITQVLKASPETKAVEYEVSCQNTHVDNVLGASREDIESIQKVLEAKAFKCTRLELPFAFHTCQMDAVIDELEALAETVPFKAPSIPVLSTMLGTAVFDGKTINANYLRRQTRNTVRFADAIEAARDMGIVDDQTVWVDVGPHPVCVGFVKKLIPTARIGSSCRRNEDNISTVVKTLVTLHAAGITPHWNEYYRHNETAYTLLHLPKYAWNETNYWIPYYGTWTLDKAFVKFGRKDGSVPAPVSAAPSFRTSTIHQVTSETIEASTASLHVLSDIQHPEFLAAVYGHTMNNCGVATSSIWTDMAWAVGEYLYKKLQPDVKEVHMNILDLEVLHGQVASKTKGAYQPLALEANLDLDTQIMSLAWYDVSVETGERDAESFATASVRFEDPDVWTSEWNRQTHLVQGRIETLQQLANENKANRISKRMAYTLFKKLVDYAEHYRGIDNMILHEYEAVADITLANDRHGTWHTPPHWIDSVCHLAGLIMNGSDASNTDDYFYVTPGADAFRLLKPLEAGGKYRSYVRMFPVPIEAGMHAGDVYILQDDTIVGVLTQIRFRRVHRLLLDRFFSAPTGDKVKNRDAHQTRPAASAPPKKTAAPAPVKAAPAPAPAPKPAPKAAPLVMQIHETASVSDSLSSSSTLSSSEASPMGNTTPQTGNTTPQTGNVTPKMEELDTGVVGQCLQIMSRETNLEMSELSADAAFTHLGVDSLMSLVLSEKFRNELGVDIKSSLFLECPTVGEFKEWVDQNC | |
| *stpB* | MATPSPLEESFWQEYLSDQAAKLPPLSHVEDVTDRVVRIMGGNPGTMKLQGTNTYLVGTGQSRILIDTGEGSPEWIQRLIRVLQDRGLDISHVLLTHWHRDHTGGAPDLVAFDPSFAHRIYKNQPDRDQNPIQDGQVFAVEGATIRAVFTPGHAVDHMCFLLEEENALFTGDNVLGHGFSVVMDLAVYMNSLDYMAAKGCATGYPGHGAKIANLPAKMHEYIHHNEVRIQKVLSALTWKGTGMKGGMTLQEIIRSIYGDVQGDIADYALAPFLTQILWKLAEDGKVGFSPGEPKKARWFGLGGVRKATSIAASVVSDSD | |
| *stpC* | MAIKSPAAPVLIIGAGLSGLAAGRILANHGVPTIVFEESSPELGESFAMGLRDWCYQPLLEALGGVSLKSMIKAVAPDRHVGGSGLVDFCMRDNATGNILVTTPDDKRPVVTRANRNAVRAWLADCGDDDLDVRYHHKLASVSGTLGNMTAVFENGAQYNGCLIIAADGVDSAVRTQLLPGLVPQILPAVIYNGRFQLSRQEFDQHIKPRTNGSNVLSGIGDGFNTNITVCNMTDAKVNLDWSYSRRARGDTDALFDNSEGAKTRGVTGTIPEALVEELSSYQLADPWGRYVNAEAIKQHSVFQWVSRCVFVPTEEAKKNGQQGVVFVGDAWHTMPVFSGEGGNHGVLDSVELAMAIVEKDSLEQAISAYYDGARHRVEEAVKRSKTRFFWLHRPMAEWQEVSEKRKMAEERVAKAGTVTA | |
| *stpD* | MPPPASTATAESKPKAKAKASAKAKESYKTKRFHYDHWVLGIVSTYAWGCPTSRYTLPQYRANIGENHLDIGIATGYYLRHGGIPLTTNITVVDTNKPAMEYALERLGRRDARAIVADILKPLPIQDKFDSVSMYYLLNCIPAGVEHKCAVFSHIRNNMTPNGVIHGASIVGKGVRVDNKFAARMRGKLLAAGIFQNKEDSPFDFEKALRQNFHEVEVRVVGTVFLFRAACPRLDDADATSPDS | |
| PKS1 | MAYTSHVLLFPHEHTALLEAIHDLSVRARTRPKLRTFLDKASRVLHREVVALTRPERDSIGEFDDLVDLAERHVRKPDVVVETALLTSFQIGQLLVFAENHPGALSDKPVDTFFPVGYGMGLIAAAVAATATTEDAIIALGLEAVAVAARLAADLQRTSKDIEDSEDAWSRVIAGVGQEELEQQLAMANANCRPLQTAYIAQILPDAIVVMGPPSTLDTLSHGQTWRMAQPTARSTVPARCIMFGPHLPPIDDAKVVGTAPVLEEAPCQRPLYSGHTTVREALAPNTCKTLGDALRVAVATIAHRPVQVELAFLDLAAGLRETGNKDVALTTVGSSAVTSKLCSILEKHGQNITLAECEPALAAFGNDLDSVSRHEIAVVGMSGRFPESDSLDELWSLLSSGKTTHREVPPSRFNVDDFYDPSRRTHNALLSRHGCFIRKPGDFDHRLFNISPREAAQMDPVQRMFLMTTYEALEMAGYSPTDDGKDPMRIATYFGQTVDDWKTINEQQGIDTHFLPAVNRSFAPGRIAHYFRWAGGFYSIDTGCSSSATALCLAREALASGECDAAVVGGGTLLNAPEWFAGLSQGGFLSPTGACKTFSDAADGYCRGEGVAVVVLKRVADAVRNKDNILAVVAGAARNSNAGAGSMTYPGEQAQAALYRRVLRQAGVRPQDVSVVEMHGTGTQAGDKVEMQALQHVFASPVQARRQRPLVVGAIKASLGHSEAASGVVSLIKSILMLRHNTIPPQPGQPFTLNPHLRPIMGADIQLANGQTWTRNGTTPRYALVNNFDAAGGNVSMLLHDTPSFAEQPPPTRPDERSRHVVVTSGRTVTSQKANKSLLRAYLAKYPDVSLADLAYTTTARRMHHVHREAYVARSSEELLRQLDQRSADDEISSEPTPSVVFAFTGQGSQYMGMGGGLYRTSPTFRRLLDSYQSLCNAQGLPCNILDVILASGEDVNSEWAARDMQVATVALEIALARYWELLGVRPTLVIGHSLGEYAALCIAGVFSVGDALAMAYERAALIFTRCEPAEASMLAVGLPASTVRWRLRDSAAVAGCEVTCVNGPSSTVVGGPVAAVEALQVYLNSDNATVATSRLRVPFAFHTCQMNPVLDDLELAAKSVKFNTPDLPVASSLLGRVVQPGEDGVFNARYLRRHTREPVVFLDAVRACEEQGLIQDQSLIIEIGPHPACIGLISSSLQKVTVRGYPSLRRGRDDWESISQCAAAAYCTQLPVAWAEFHKDHVDSLRMVTDLPTYAFDKQEFWHSYKTKAVLESHASKASPVSPPASSRLSTTCLHSVEQYRRDGSRLLATFGVDLTDRHLSSAIGGHVVDGVAICPASIFMDMAYTAAAYLDNKSRNASSSSSLGGYELTNLSMLTPLVLRDDVGLPRVFVDAVLDPSTDAVSVRFLSRKDEVASPVEHGSCLIRLNQPEAATTHTWSRIRSLVKARVRSLDESARPKQVHAMDKSLFYKLFSEIVDYSVPFHAVEEATVAADFQDAVMTIHCDSAAELGSFTCNPFAIDALVHIAGFLLNVNVSKPKNEVHIANHIGSLRVLGDLESDGPFRAYTAIRDQDAKTGTSLCDVYLTNAQDKLVALCTDICFKRLDRDFFAILTGSARAVPAKPRTNQTTPRCRWQDRSSSSSDDTSSETDTPDASSVSSLSDTVDLSAALLAIVAEHCGMTVHELRRSKGVTFSQFGVDSQMSISILAEFQRTTDVELPAAFFINFPTPDAVNKELGNEQLEETKVQERKPRSPHQLPAKVRRGTERSPEPMGSSAHLLSLVAESLGLEAGDLTSSTKFQAIGMDSMLSIRITSQFYRDTGIELPAAFFSEHPTVAAACAELDGPVETAAPDERLRNSSAVSTLHIPIIPTVSAANVSALARQHKIDNAVSRAVLIQGQSCSCDAPLFMTCDGPGTVETYIHLSALPNGRRIYALESPFLESPETFDLSIQEMASIFIRTIRRFQPHGPYLIGGWSAGSVYAYEVAHRLTTQGETIGALIILDMRPPCPSPISIVTDDVFDKAGVFEGINRARDLPKDLLLKKKAHLMGTCRAMSRYNAPAFALNRQPRQVAVVWAKLGVENSKDAPIAAMCRPGVDIGKKPEQMGPAEFERYLSSWFYGRREQFGTNGWEDLLGDNISVHAVDGDHFSMMFPPFSTAVGKIVSDTVALGLNTDLTEIHVAVGCDIAYTT | |
| PKS2 | MGHPGSLYLFGDQTFDAEPHLYKLLESKNSNAILRDFLEKAYDALRVQIFQLPKPMRDELPRFTSIEDVLLSKRCMGSSGCVPLDMAVTCIYQLSSFILSQAGKEYPTSEDACILGLCTGALAAVAVSCSRSLTELTPRAVTAVVIAFRVGVCVSQMRLRLVSPSELSRTWFMMVAGQSASQAVHRFREESSLPLTAKPFISAYTPNGLAVSGPPSSLDELVSSTHFRGLKYKAKHIYAPYHAPHLYSMADIEDVLAPLDMDRGPQSLKKIPLLSSAGDWIQEANPGALFKEALEQILLQPIRWNKIMDGLRTGPGSLWEAKTCKIEVFGSRADQLIYSALKQSPTHSRMLSSRSSIHKKTDLGSLDESGIQSRPKIAIIGMSGRFPGGANNNEQFWDLLREGLDVHEEVPPLHWSKAHVDATGSRKNTGATPYGCWLSDPASFDARFFNISPREAPQIDPAQRIALMTAYEALEQAGVVPDATPSTRRDRVGVFYGVTSNDWMETNSAQNIDTYFIPGGNRAFIPGRINYFFKFSGPSYAIDTACSSSLAAIHTAANSLWQRDIDTAIAGGTNVLTNPDFTAGLDRGHFLSRTGNCKTFDDSADGYCRGEGVGTVILKRLEDAVAENDPILGVILGAYTNHSAEAESITRPHTGAQRAIFSKILNASAVSAETVGYVEMHGTGTQAGDATEMASVLEVFAPQNQPARQSPVFLGSAKCNIGHGEAASGVSSLIKVLLMMKNNLIPPHVGIKTKINHKFPTDLKERNVHIAREAVPWQASGSKLRRAFVNNFSAAGGNSALLIEEAPSPTHGDDQQHVPGANLVAISAKTGPSLQGNLRSLQEFLQRNKDNIKVGQLSYTTTARRVHHPHRVMLAGSSVDELLGQIDIAVRDQLGMTRPKNKQPKVIFAFTGQGAQFPGMGRQMMETYAMFRTELRQLDHIARNLGFPSIIPVYMASEDQALDEFPPIQVQLASVCMQIALARLWASWGIMPEAVVGHSLGEYAALNVAGVLSDSDAIYLVGTRAKLLQDLCTRGTHSMLVVRASADTISTTLKNTPYQIACMNSPIETVLAGPDQEITLASQILGDAGIKTTKLKIPYAFHSSQIDPILAPFEKSAHSVRYLTPKYPVLCPLDGTLVTTDGTFGPAYLGRHCRQPVNLLQALQTARQGSIITEQTILLEVGPHPAVSGMVKATLGQQMTVISALNRKSAKDVLADALKVLYQAGADIAWTVYHNDFKASQRVITGLPAYSWDLKPYWIQYVNDWSLRKGDPPLVVNETEKTREKLESTTIHRVVEESSSNCGTKQHIIVEADIARSDLNPLVQGHMVDGIPLCTPSVYADMALTVGRYLVLQKQGSGVNTSNNHVDVSDMTVFKALIAKPQGPQPLQGHIEVDWKSNQAAMKFVSFDSKGKPQEHAKCTIRFTDRSLLLDKLQKESASIKTSMQRLRQGIAQETAARFNRPMVYRMIRPLAQFHDDYRAIDEVVLDSNTLEASASVSFGAVKKGGQFALHPAIIDAFTQSCGFTMNCNDHTDLDKEVYMNHGWGRFQVFEDIHFDRPYTSYTRMVEGKDKLWYGDVVIFDGETIVAAFQQIDIQCVPRRVLQVILSLESGISPSKRGAAGAPVSSKSPKTASTTKTAIASNLNKATIPEVETARSSSRISTALQIIADESGVALEELTDNSAFADLGVDSLLGLTISARFREELDLDLEFDALCITYPTIGELKSFIAPAAMDTGKTEVQHESSALISDKAYETVAFSHLPTNDVAVLKSKSDHNQIDFTNVLNIVSEESGIALEELTGDTYFADAGVDSLLSLVIVSRFREELDIDIPMDSLFVDCATVDDLRSFLLTTPSSGSSRSDENTDGNSIPTTGTDKMFDTNVQVHAITLTPPTSGASSDTEDCEELQPLVVVPRASSLVLQGNPKTASKTLFLFPDGAGSASSYASIPTIASSTCVIALNSPFLKEADKLRAAFLDDIVIDGYLAEIRRRQPFGPYHLGGWSAGGILAYRAAQILIKSQEQVSSLTLIDSPPPLKGLDRLPQHFYDYCESLLLFGHKSSSPLSTGKPPEWLIPHFNATIDLLHEYRAEPMQNKGLTSIPKVNIVWAAACIADNPRLPKLPPHPDDTGGMKFLLERRTDFGGNGWETLFPGSESSITINKVDGAHHFSMMREPYSQKLAEFLRQAMA | |
| PKS4 | MVTAADVGHREPEIVAFGDLASSECIVPLQNLLRSKSNADLTDFFARVGFGLRQYLGSLSSSTQELFPNFTTLNDLVYRWEEATGRPALHVFLLSVLQSAQFICHYSGKQAREFPSAASTCIISSCVGEFVAAAASCSKSTSSFVPLAVEAALTAFRTGLKSYLIGSAIAAGSKDVIETWSVSVSPTTDASVEEMLSMFAESRGETESCRSIWVSASTPTGATSLGGKPSVLSSFVNANKDKLRVKPLNVTSPFHAPHLFSERDVDEIMRDVAADVGKDAMHRITLISSQTASEAETDLTFIGRLRMAVHGVLRDQLDLNLIFESCGTYLTQLGSRRCRLTTFTYGATPILAATLANAFTIQLDIEDASQSSVDESLPTAQFSESKIAIVGYSGRFPSAESNEAFWELLRAGRDVHREVPSDRFDWRTHYDADGKIPNTSRVKNGCWVDNPGLFDARFFSMSPREAENTDPAQRLAILTTYEAMEMAGFVPNRTPSSQADRVGVFLGSTSDDWREINASQNVDTYFIPGGNRAFGPGRISYYFRLSGPSISVDTACSSSFVAIQAACSYLRSGQCDTAIAGGTNILTNPNIFSGLDRAHFLSTTGNCNAFDDEADGYCRADAVGTVMLKRLEDAEADNDPIFGVIVETNINHCGHTESITRPHEGDQAALFQQILRQANYNPLDVSVIEMHGTGTQAGDATEMNSILSTFVPQRERTKAEPTRPLYIGSAKASVGHSEAASGVTSLIKVLLMLKHNEIPPHCGIKTKINRSYPLDLTERGVHIAMKPTPWLRHSKETKRAAFLNNFSAAGGNTAMLLEEAPRKLRNIDNPSGLHSHLVAVTAKTPKSLVGNAHSLVAWLRKNGDAALADLSYTTTARRMQHDYRMMVSAQSIPSLIQSLESLSYQKPQSLKAILPPTKRPKLVFIFTGQGSLYIGMGKNLYQTYNSFCQDILQLNHLAESCGFLSFIGLIDGSTSVDVHTVSAVTSQLALLCVQIALVKLLGRWRIRPGAVIGHSLGEYAALYAAGVISAGDAIYLVGKRATLLEQHCKKGTHGMLVVKGSQQETERLLQSVENDFEVACANHPTAHVVAGPKDQIPAIVAEARKAGLSTVELNVPFAFHTNQINPILSEFDMAAAQAGLVFNPPKIPVISPLLKRVVCQGEENTLNMSYLVRASRGMVDFSTALNTAQRDGLFGARAIWLEVGAHPLCGTMVKQTLGPEEEVLITLKEKVELSKTLLKSVEALYLAGWDIDWNEYHRELPVTPQVLELPHYAWDLKNYWIDYKNDFCVNRNGHLTKPDAIEAPIRYKYISPAAQKVIEEVHEKSESSMTVESDIFDPDLLPVLHGHAVNGAFLCPSSLYAEIGLTIGAYLLSEQKMCRETTGLEVRDLHIDQPLIAKPDEKTHCFRVQVHADWTSNNITLAIFSVDTSGSKTVSHATLNVCVAPHQRWLADWKRNTYLVTSRLKALASDENSQKLRRRMVYKLFASLVDYDQAFKGMSEVLLNSDELEAVSTVNFQVSDSRGEFGIDARWIDSLGQVSGFIMNANDTMNNQEKVFINHGWEKLRYAEKLDASKTYQAYCRMQLAEKTTYIGDVFVLDDDRIVAVYEGVKFIGMGRQVLNHVLPPKRSDTGKTPIVPTPVRRVASLSNLNENVRQSRSLERVRRTESKMPDAHMGVDRMTEIMCQEIGVPASDLEPESQLADLGIDSLLSLTITSRIREELGMEVLSTALLECQSVGELQSLFGCSESFISATTTDFDSLSNSSGESGDHESSLSTPSEICGSEWSEDMPVRRTLWETIAGETATSIEELTPSTTIADIGIDSLLAITMSVSLCEKFSAHIPSTLILGCETLLDIETALCKVLGIVQGSCAASPRNRTDLRNVPRKPSSTAGDLGISDNPPATSALLSGSPRTAKSILIMFPDGSGSAASYATLAPQLPRDTALYGLNCPWRKNGLELTRSSITVPQMVASQIVEVRRIIESHKQHGWRNNTSGTPNLILGGWSAGGILAYEAVQQFAAEGITIRKLVLLDSPDPVNMRAPPRKMSYFLEALSKSGGRLGKAPDWVLDHFDGMMGVLGGYRPAPLPESLVLDTLLVYARKGAWNEYDEPNTCLRANEDHDVSWILRDRTDFTAGGWKRLLGTRQLSVKIVDDADHFSLMEDERRTKRVGNFIIAFL | |
| PKS5 | MAKIFCHVFGDCHGEKYDLLNKYFSKQQSGILATALIDQAAISLQSEVISSKYPQQAPISPFNTIKQLNEQHHASNVKHLGIDNALNCILQLCHYVDYVEKSEQAFCDPHQSIFLGGGVGLLCAALIASSPTPATLISNALQAILLSFRLGSYVGSVAERISNPNQRSNPVQEWAYNFPGLDLESAELYLNEFNESNTISKVRRAYISGISRGHVQISAPPSILQKIIDQGVFTCNPIRCSVYGPYRAPHLHSSIEVDDILSAADSNLQERANKCRLKSPTVSSGETWLSGCETTLVVHAATHQYLSQRFLASKLARNCASALTASKSKHCAVIAHGCHELAEELIVTLTGLTSIGFTHERSCQTTERIAGENSPCELNWNSKLAIVGMGGRFPDADNTDELWDLLCDGDDVHRIIPEDRFPVESHVDPEGKATNTSYTPYGCWVKSPGLFDHKFFNMSPREALQTDPMQRLAIVTAYEALEMSGYTPNRTPSTRLDRIGTFYGQTSDDWREINAAQEIDTYYITGGIRAFGPGRINYFFKFKGPSYNIDTACSSSAAALQVACTSLLAKECDTAVVGGLSILTNPDLFCGLSRGQFLSKKGSCATFDDAADGYCRADACASLVVKRLEDAISDKDKVLAVILGTATNHSADSISITHPHGPTQSSLYSAILDKAGVDPIDVDYIEMHGTGTQAGDSTEMASVLNVFAPENRRRDLDRPLYLGTVKPNVGHGGAASGVTSVIKLLKMLQHDTIPPHVGIKTGSVLNRAFPKDLAARNVNIVFKNTPFKRRDGKPRLAFVNNFSAAGGNTALLLEDAPPQLPPVPDPRDAHIISVTGKSKSALMRNAEKLAGWMAMHPEIPISDVAYTTTARRIQYDWRISVAASTLQEAQAAIETSIRDDKDCHVGSSKPPLCFVFTGQGSSYKGMGQEYYKSYSCFRKWICDFERIAHMHGFPSFMSLINCDDSESCDTSPIAVQLAILCFEMAALELWLSWGVKPAAVLGHSLGEYAALYASGVLSASDTIYLVGERAKLVVQHCSRDSHAMMAVNAPVAGVLPILDQYNVQIACLNSPNETVLSGSSIDISRAAEQLKTDGLCCTLLRVPFAFHTSQMDPILDKLDRLAHNVSFSNPEVPFVSSLLGRPVDEIGPTYIRDHARGVVRFSDAVCSAQKLASPDSAMLWLEVGPHSSCTNMIRTIIGTSAIAIPTARRGESMHKVLCHSISCLHGCGVDLDWNEFHRDFSKSTTLVDLPSYSFEKKNHWIPYEGNWTLSRRKGSTSAALLQNRKLSTTSVHQIVRQVVLAGVAMIDTETNLSRDDIRPTITGHRCNGVALCPASLYAEIAMAVSEYAYKLICPGHGPPGLNVADLSIQKALLFDDKADAHILHCTVQTSGTGDHASVVFHTRNGLLKNEHARCTVYYGDLRVWAAELARVNYLVEARIDALASGNASHAISKYNQPAAYQSFASFVDYGPQYQGMKEVLLCAKSGEATAELSFQASNEGNFFFNPYWIDGCCHLSGFILNAKEAADSSEEVFISEGWESLRFIERLREGTTYRSYTRMQPVHGMSLVAGDVHILRDKCIIGVANGVKFRSVPKRALHLLLPPTITCRTSNSQRQSIAELALQTDNVNPKITKKGQGYATFPALYRTTAQTSLMDEFLRIVQVEIGCETEDLGDNATFTSLGCDSLMVLVIASRVHEELEINIRPQAFLEYPTIGDFKEFLASLRITDTRSSDASTSSTVSGVTDEPDIGQLSDSESSTSRLGDKTCEEQEETKKSLDTAMADAPIRTIERVECLRDQFPNRKAKSVLLQGSRQIAFKNLFMIPDGSGSAYSYSELGSLGSEWAIWGLVSPFMKTPHEYTCGVEGIARKFLEEMKQRQPVGPYVLAGWSIGGTIAYEITRQLTRSDEQVSHLILIDAPCPLLDDPLPRHLGRWFADIGIIGSKMGASLCIPDWILPHFDMSAKAFADYKPVPISRDHCPITMAVWCEDGVCRTQQDPRPHPYPTGHALFLLENREDFGPNRWDELLASEKIQTKRMRGNHFTMMREPLTQDLTAIIYQAVMSDKP | |
| PKS6 | MYRSETTDRRTLVVFGDQTSNVTSIAKQLFLGVPQSAAATEFLRDASAALMAQRDRLRPFQRNHLPDFKNLHDIARIYDEASGVCHPSITSVMLCATQLLQLFRYFEEHRPGPNENHHVTTVGLCTGSLAAAAFAASNGVEDLRWLAVPTISMAFQLGLQAATASSMLYEQDAARLGSWSVAIPNMPEDEAISALSVYDAGHHLSLRHRCFVSAVGLKSVTISGAPPALRAFTDTLAASQPERRLVELPVFAAFHAVHIHPFLDFPSFLSRCGVSLPLLARFKPVHRLLSPLTGEPVVANDAFGLFKTAVCETLQAPLRLDLIVEGFAKFIQDEKISSISVEVVSPTTVADGIVAALVQQTQASVSSRPIVALPSAGEIRSNPPFNNVPLAIVGMSGRFPDADSAEALWDLLAQKIDAHRVVPKDRFDVNVHVDPAGKAKNTSWTPYGCFINRPGEFDPRFFNMSPKEALQTDPMQRLALVTAYEALEQAGFVPDRTRSSASHRVGTFYGQTSDDYRDVNSAQDIGTYFITGGIRAFGPGRINYFFKFSGPSYSVDTACSSSLAAIQLACTAIWNGECDTAVAGGLSILTSPDLFSGLSRGQFLSKTGSCKTFDNAADGYCRADGIGTVVIKRLKDAESDRDNVLAAVLGAGTNHSAHAISITHPHAETQSDLYRQVLQQAGVDPHDVSYIEMHGTGTQAGDGTEMKSVSDVFAPPSSKARASDNPLYVGAIKSSVGHGEASSGVISLIKAMTILKKSLIPPQAIKGELNVGFPDLKARNIRIARHMTPFPERKGIKKTVLVNNFSAAGGNTALLIQEPPETRIEHNKEPRNAHPVTVSGHTHSAMLNNLDRLIEFLANNSETSPLDLSYTTTARRRHHNFRATVVESSTPRILAALKLKRASLAEGTFIPPKKPRSVVFTFTGQGSAYPALAREIYNTSTQFRADVWHFDGIARQQGFPTFLPLITDASLADINVLSPAQTQIGLVCIQVALARLWSSFGIKPSAVIGHSLGEYAALQVAGVLSVSDMILLVGSRARLMESKCERNSHAMLAVAAPGCSVQSMAAECLDGVDLACSNTPRESVFAGEKSKIEQLAQKLNECKIRSTQLRVPYAFHSTQVDSILEDFGNIASVVNMGVPHTTVISPTRGRVLDAGDKIDGEYLKHHCRHTVQFAQAIEHSRQAGVITDATTLIEVGPHPICSGMVRSTLGEKTQTLSTLNRKENPWNAIVGSLASLHDAGHTVNWSEYQRDFESACRLIHLPSYAFDNKNYWIDYRNNWTLRKGDPSTAAADAQSVVADRSGNAKPYISSSVHRVVEEDYNSAQPRVVFETDLSHPDMHTAMSGHRVNGSALCPSSLYADIALTIAKYIRDQPGSGLDQDGHDVCDMEVTQPLIINPHRDQENRTLRVVTHVDKRSHILSLEYSSYGPAPALKTPVKHAMCRVEFGNRQKWSRRWVHDLHLVKDRIAGLKVATQSGKASSITKRLAYRLFANLVDYAPDFQRMGQVWLDGVGREATAVVSLDRRETDANFTFSPYHLDGLLHLSGFIMNGNDDIDANEAVYISHGWQSLRLAEPLVPDTNYHVYVKMLPVDKTMVAGNVYVLHDDKVVALCESIRFQRVPRAVLDMLLPPVPTSRPRRAAGPAPERFKHGGSPPVVKVAERVAPTKKQLPLAPAQLPATSGTVSTPQSELFMQVIANEIGLGTGELNVTDNLVDLGVDSLMSLALAGQLVEQFGVNVDHGELMQCASIKDLLRLLQDKQEPPPASTQNEEHAGTSSSSSGSSASSSAGGLGTVTPPTRATTPAVKPADDMGRLVYSIVLEETGMEGEDFQSSAELSDLGVDSLMSLAILAKLREAGVDLSPTFFLDNRTMDDVFRTLGKGKDPGAEEEQQVIDSTASQASNLRSEAAEATAAAAAGSRPHAQCILLQKATASPAGQSLFLFPDGSGSPFAYAALERLDPTLDVYGLACPFLKSPSAFKDHGIEATAAEYIKTLLAKCPAGSLHLGGWSVGGVLAFEAAKQLTEVHGRQVASLVLIDAPCPLTLPPMNGRLIKFLDSLQLFAPPGERNARSRADTEKHRMVLEHFDATVECLGRYRPASMAMAIRTLVIWASDGVVGHGDAIPDRMLLDDPMANWILQDRGTPGPHGWDKLLPSHNLEIVTTPGNHFSMMVGGNVRALSLGLQAHFQGLNANNVGK | |

# Analytical data

**2,4-Dihydroxy-6-methylbenzoic acid (Orsellinic acid) (2)**

R*_f_* 0.12 (DCM/MeOH, 95:5).

IR (ATR): *ṽ* [cm^–1^] 3543, 2648, 1615, 1495, 1455, 1353, 1254, 1211, 1171, 995, 891, 825, 796, 728, 624, 582, 471.

^1^H NMR, COSY (600 MHz, DMSO-d_6_) *δ*_H_ 13.43 (s_br_, 1H, O*H*), 12.12 (s_br_, 1H, O*H*), 10.15 (s, 1H, O*H*), 6.17 (dd, *J* = 2.5, 0.7 Hz, 1H, 5-C*H*), 6.11 (d, *J* = 2.5 Hz, 1H, 3-C*H*), 2.39 (s, 3H, 1-C*H*_3_).

^13^C NMR, HSQC, HMBC (151 MHz, DMSO-d_6_) *δ*_C_ 173.3 (1C, 1-*C*OOH), 164.5 (1C, 2-*C*_q_), 162.0 (1C, 4‑*C*_q_), 142.9 (1C, 1-*C*_q_), 111.0 (1C, 5-*C*H), 104.8 (1C, 6-*C*_q_), 100.5 (1C, 3-*C*H), 23.5 (1C, 6-*C*H_3_).

HRMS (ESI) *m*/*z*: [M–H]^–^ Calcd for C_8_H_7_O_4_ 167.0350; Found 167.0354.

The analytical data are in accordance with the literature [7].

**6,8-Dihydroxy-3-methyl-1*H*-isochromen-1-one (Saccharonol A) (7)**

R*_f_* 0.51 (DCM/MeOH, 95:5).

IR (ATR): *ṽ* [cm^–1^] 3254, 3079, 1680, 1644, 1622, 1573, 1506, 1482, 1383, 1360, 1253, 1236, 1182, 1154, 1106, 1071, 694, 864, 833, 790, 716, 689, 621, 549, 529, 434, 415.

^1^H NMR, COSY (600 MHz, DMSO-d_6_) *δ*_H_ 10.95 (s_br_, 1H, 8-O*H*), 10.85 (s_br_, 1H, 6-O*H*), 6.47 (d, *J* = 1.0 Hz, 1H, 4-C*H*), 6.33 (d, *J* = 2.2 Hz, 1H, 5-C*H*), 6.30 (d, *J* = 2.2 Hz, 1H, 7-C*H*), 2.20 (d, *J* = 1.0 Hz, 3H, 3-C*H*_3_).

^13^C NMR, HSQC, HMBC (151 MHz, DMSO-d_6_) *δ*_C_ 165.7 (1C, 6-*C*_q_), 165.5 (1C, 1-*C*OO), 162.7 (1C, 8‑*C*_q_), 154.2 (1C, 3-*C*_q_), 139.8 (1C, 4a-*C*_q_), 104.3 (1C, 4-*C*H), 102.4 (1C, 5-*C*H), 101.4 (1C, 7-*C*H), 97.9 (1C, 8a-*C*_q_), 18.9 (1C, 3-*C*H_3_).

HRMS (ESI) *m*/*z*: [M+H]^+^ Calcd for C_10_H_9_O_4_ 215.0855; Found 215.0877.

The analytical data are in accordance with the literature [8].

**6,8-Dihydroxy-3-(2-oxopropyl)-1*H*-isochromen-1-one (4)**

R*_f_* 0.44 (DCM/MeOH, 95:5).

IR (ATR): *ṽ* [cm^–1^] 3357, 3209, 1714, 1687, 1651, 1609, 1582, 1459, 1411, 1357, 1323, 1287, 1203, 1150, 1059, 1024, 964, 868, 795, 660, 575, 521, 467, 456, 418.

^1^H NMR, COSY (600 MHz, DMSO-d_6_) *δ*_H_ 10.91 (s_br_, 2H, 8-O*H*, 6-O*H*), 6.56 (s, 1H, 4-C*H*), 6.39 (d, *J* = 2.2 Hz, 1H, 5-C*H*), 6.34 (d, *J* = 2.2 Hz, 1H, 7-C*H*), 3.77 (s, 2H, 1‘-C*H*_2_), 2.20 (d, *J* = 1.0 Hz, 3H, 3‘-C*H*_3_).

^13^C NMR, HSQC, HMBC (151 MHz, DMSO-d_6_) *δ*_C_ 203.4 (1C, 2‘-*C*O), 165.8 (1C, 6-*C*_q_), 165.2 (1C, 1‑*C*OO), 162.7 (1C, 8‑*C*_q_), 151.2 (1C, 3-*C*_q_), 139.2 (1C, 4a-*C*_q_), 106.9 (1C, 4-*C*H), 103.0 (1C, 5-*C*H), 101.9 (1C, 7-*C*H), 98.1 (1C, 8a-*C*_q_), 47.0 (1C, 1’-*C*H_2_), 29.8 (1C, 3’-*C*H_3_).

HRMS (ESI) *m*/*z*: [M+H]^+^ Calcd for C_12_H_11_O_5_ 235.0601; Found 235.0603.

The analytical data are in accordance with the literature [9].

**(*S*)-6,8-Dihydroxy-3-(2-hydroxypropyl)-1*H*-isochromen-1-one ((+)-Orthosporin) (5)**

${\text{[}\text{α}\text{]}}_{\text{D}}^{\text{2}\text{1}}$ +42.2 (*c* 0.23, MeOH).

R*_f_* 0.34 (DCM/MeOH, 95:5).

IR (ATR): *ṽ* [cm^–1^] 3460, 3064, 2979, 1678, 1644, 1633, 1575, 1495, 1397, 1283, 1267, 1226, 1173, 1149, 1115, 1065, 1047, 1007, 936, 841, 794, 757, 688, 546, 487.

^1^H NMR, COSY (600 MHz, DMSO-d_6_) *δ*_H_ 10.99 (s, 1H, 8-O*H*), 10.85 (s_br_, 1H, 6-O*H*), 6.48 (s, 1H, 4-C*H*), 6.36 (d, *J* = 2.2 Hz, 1H, 5-C*H*), 6.30 (d, *J* = 2.2 Hz, 1H, 7-C*H*), 4.82 (d, *J* = 4.9 Hz, 1H, 2‘-O*H*), 4.01–3.92 (m, 1H, 2‘-C*H*), 2.53 (dd, *J* = 14.4, 5.3 Hz, 1H, 1‘-C*H*_2-A_), 2.47 (dd, *J* = 14.4, 7.7 Hz, 1H, 1‘-C*H*_2-B_), 1.13 (d, *J* = 6.2 Hz, 3H, 3‘-C*H*_3_).

^13^C NMR, HSQC, HMBC (151 MHz, DMSO-d_6_) *δ*_C_ 165.7 (1C, 1‑*C*OO), 165.6 (1C, 6-*C*_q_), 162.7 (1C, 8‑*C*_q_), 155.4 (1C, 3-*C*_q_), 139.7 (1C, 4a-*C*_q_), 105.4 (1C, 4-*C*H), 102.6 (1C, 5-*C*H), 101.4 (1C, 7-*C*H), 98.2 (1C, 8a-*C*_q_), 64.0 (1C, 2‘-*C*H), 42.7 (1C, 1’-*C*H_2_), 23.4 (1C, 3’-*C*H_3_).

HRMS (ESI) *m*/*z*: [M–H]^–^ Calcd for C_12_H_11_O_5_ 235.0612; Found 235.0620.

The analytical data are in accordance with the literature [10]. The absolute configuration was determined by comparison of the specific rotation with literature data [9].

**(*R*)-6,8-Dihydroxy-3-(2-hydroxy-4-oxopentyl)-1*H*-isochromen-1-one**

**((+)-Citreoisocoumarin) (6)**

${\text{[}\text{α}\text{]}}_{\text{D}}^{\text{2}\text{1}}$ +91.6 (*c* 0.06, MeOH).

R*_f_* 0.37 (DCM/MeOH, 95:5).

IR (ATR): *ṽ* [cm^–1^] 3165, 2923, 1680, 1622, 1578, 1509, 1462, 1361, 1282, 1238, 1169, 1064, 1025, 987, 669, 852, 798, 716, 694, 649, 619, 595, 574, 548, 531, 504.

^1^H NMR, COSY (600 MHz, DMSO-d_6_) *δ*_H_ 10.99 (s_br_, 2H, 8-O*H*, 6-O*H*), 6.48 (s, 1H, 4-C*H*), 6.35 (d, *J* = 2.2 Hz, 1H, 5-C*H*), 6.30 (d, *J* = 2.2 Hz, 1H, 7-C*H*), 5.04 (s_br_, 1H, 2‘-O*H*), 4.31–4.22 (m, 1H, 2‘-C*H*), 2.62–2.52 (m, 3H, 3‘-C*H*_2_, 1‘-C*H*_2-A_), 2.49–2.45 (m, 1H, 1‘-C*H*_2-B_), 2.10 (s, 3H, 5‘-C*H*_3_).

^13^C NMR, HSQC, HMBC (151 MHz, DMSO-d_6_) *δ*_C_ 207.3 (1C, 4‘-*C*O), 165.9 (1C, 6-*C*_q_), 165.6 (1C, 1‑*C*OO), 162.7 (1C, 8‑*C*_q_), 154.6 (1C, 3-*C*_q_), 139.6 (1C, 4a-*C*_q_), 105.8 (1C, 4-*C*H), 102.7 (1C, 5-*C*H), 101.5 (1C, 7-*C*H), 98.1 (1C, 8a-*C*_q_), 64.6 (1C, 2‘-*C*H), 50.4 (1C, 3’-*C*H_2_), 41.0 (1C, 1’-*C*H_2_), 30.6 (1C, 5’‑*C*H_3_).

HRMS (ESI) *m*/*z*: [M–H]^–^ Calcd for C_14_H_13_O_6_ 277.0718; Found 277.0720.

The analytical data are in accordance with the literature [11].

**7,9,10-Trihydroxy-3-methyl-1*H*-benzo[*g*]isochromen-1-one (nor-Toralactone) (3)**

R*_f_* 0.42 (DCM/MeOH, 95:5).

IR (ATR): *ṽ* [cm^–1^] 3017, 3009, 1679, 1642, 1581, 1511, 1366, 1348, 1285, 1234, 1156, 1103, 1019, 1001, 960, 870, 835, 775, 660, 629, 575, 522, 417.

^1^H NMR, COSY (600 MHz, DMSO-d_6_) *δ*_H_ 12.92 (s_br_, 1H, O*H*), 10.27 (s, 1H, 7-O*H*), 10.22 (s_br_, 1H, O*H*), 6.96 (s, 1H, 5-C*H*), 6.56 (d, *J* = 1.8 Hz, 1H, 6-C*H*), 6.43 (s, 1H, 4-C*H*), 6.39 (d, *J* = 1.8 Hz, 1H, 8-C*H*), 2.20 (s, 3H, 3-C*H*_3_).

^13^C NMR, HSQC, HMBC (151 MHz, DMSO-d_6_) *δ*_C_ 166.7 (1C, 1‑*C*OO), 162.9 (1C, 10-*C*_q_), 160.9 (1C, 7‑*C*_q_), 158.6 (1C, 9‑*C*_q_), 152.3 (1C, 3-*C*_q_), 141.7 (1C, 5a-*C*_q_), 131.8 (1C, 4a-*C*_q_), 111.0 (1C, 5-*C*H), 107.1 (1C, 9a-*C*_q_), 104.2 (1C, 4-*C*H), 101.6 (1C, 8-*C*H), 101.3 (1C, 6-*C*H), 96.7 (1C, 10a-*C*_q_), 18.9 (1C, 3‑*C*H_3_).

HRMS (ESI) *m*/*z*: [M–H]^–^ Calcd for C_14_H_9_O_5_ 257.0455; Found 257.0459.

The analytical data are in accordance with the literature [12].

**1,3,8,10,11-Pentahydroxytetracene-5,12-dione (Saintopin) (1)**

R*_f_* 0.29 (*n*Hex/EtOAc/MeOH/AcOH, 5:5:0.5:0.1).

IR (ATR): *ṽ* [cm^–1^] 3409, 2924, 2853, 1593, 1383, 1352, 1200, 1110, 768, 619.

^1^H NMR, COSY (600 MHz, DMF-d_7_) δ 15.17 (s, 1H, 11-O*H*), 7.45 (s, 1H, 6-H), 7.13 (d, *J* = 2.4 Hz, 1H, 4-H), 6.56 (d, *J* = 2.4 Hz, 1H, 2-H), 6.55 (d, *J* = 2.3 Hz, 1H, 7-H), 6.22 (d, *J* = 2.3 Hz, 1H, 9-H).

^13^C NMR, HSQC, HMBC (151 MHz, DMF-d_7_) δ 183.9 (5-C), 183.2 (12-C), 178.8 (11-C), 166.5 (10-C), 165.4 (1-C), 162.6 (3-C), 161.3 (8-C), 138.7 (6a-C), 135.8 (4a-C), 132.3 (5a-C), 115.7 (6-C), 115.1 (10a-C), 112.4 (12a-C), 108.1 (2-C), 107.2 (11a-C), 104.9 (4-C), 103.1 (7-C), 101.4 (9-C).

HRMS (ESI) *m*/*z*: [M+H]^+^ Calcd for C_18_H_11_O_7_ 339.0499; Found 339.0508.

**5,12-dioxo-5,12-dihydrotetracene-1,3,8,10,11-pentayl pentaacetate**

Due to the very poor solubility of saintopin, we additionally recorded NMR spectra after per-acetylation, which increased solubility and the resulting data further validates the conclusions drawn. Therefore, saintopin (3.5 mg) was suspended in DCM (1 mL) and pyridine (1 mL). Acetic anhydride (0.5 mL) was added and the mixture was stirred at room temperature for 3 h. The solution gradually turned from red to yellow. The reaction was quenched by addition of aqueous NaHCO_3_ solution. The mixture was extracted twice with DCM and the organic layers were dried over MgSO_4_ and the solvent was removed under reduced pressure. The crude was purified by flash column chromatography (silica, DCM/EtOAc 19:1) to yield 1.4 mg of saintopin pentaacetate as a yellow solid.

R*_f_* 0.62 (DCM/EtOAc, 9:1).

IR (ATR): *ṽ* [cm^–1^] 2925, 1771, 1680, 1619, 1600, 1372, 1312, 1191, 1144, 1066, 1023, 908.

^1^H NMR, COSY (600 MHz, CDCl_3_) δ 8.71 (s, 1H, 6-H), 8.01 (d, *J* = 2.4 Hz, 1H, 4-H), 7.80 (d, *J* = 2.3 Hz, 1H, 7-H), 7.28 (d, *J* = 2.4 Hz, 1H, 2-H), 7.21 (d, *J* = 2.2 Hz, 1H, 9-H), 2.56 (s, 3H, Ac), 2.46 (s, 3H, Ac), 2.44 (s, 3H, Ac), 2.37 (s, 3H, Ac), 2.37 (s, 3H, Ac).

^13^C NMR, HSQC, HMBC (151 MHz, CDCl_3_) δ 180.8 (5-C), 179.7 (12-C), 169.1 (Ac-CO), 169.0 (Ac-CO), 168.8 (Ac-CO), 168.3 (Ac-CO), 168.0 (Ac-CO), 154.7 (3-C), 151.6 (1-C), 150.8 (8-C), 148.2 (10-C), 147.8 (11-C), 137.4 (6a-C), 136.1 (4a-C), 130.1 (5a-C), 127.7 (6-C), 124.5 (12a-C), 123.7 (2-C), 122.7 (10a-C), 120.8 (11a-C), 120.3 (9-C), 119.4 (7-C), 118.4 (4-C), 21.4 (CH_3_), 21.2 (CH_3_), 21.2 (CH_3_), 21.1 (CH_3_), 21.1 (CH_3_).

LRMS (ESI) m/z (%): 489.1 (100) [M-OAc]^+^, 571.0 (23) [M+Na]^+^.

HRMS (ESI) *m*/*z*: [M+Na]^+^ Calcd for [C_28_H_20_NaO_12_]^+^ 571.0847; Found 571.0853.

# ^1^H- and ^13^C{^1^H}-NMR spectra

Spectrum S. 1: ^1^H-NMR spectrum (DMSO-d_6_, 600 MHz, 294 K) of Orsellinic acid.

Spectrum S. 2: ^13^C{^1^H}-NMR spectrum (DMSO-d_6_, 151 MHz, 294 K) of Orsellinic acid.

Spectrum S. 3: ^1^H-NMR spectrum (DMSO-d_6_, 600 MHz, 294 K) of Saccharonol A.

Spectrum S. 4: ^13^C{^1^H}-NMR spectrum (DMSO-d_6_, 151 MHz, 294 K) of Saccharonol A.

Spectrum S. 5: ^1^H-NMR spectrum (DMSO-d_6_, 600 MHz, 294 K) of 6,8-Dihydroxy-3-(2-oxopropyl)-1*H*-isochromen-1-one.

Spectrum S. 6: ^13^C{^1^H}-NMR spectrum (DMSO-d_6_, 151 MHz, 294 K) of of 6,8-Dihydroxy-3-(2-oxopropyl)-1*H*-isochromen-1-one.

Spectrum S. 7: ^1^H-NMR spectrum (DMSO-d_6_, 600 MHz, 294 K) of (+)-Orthosporin.

Spectrum S. 8: ^13^C{^1^H}-NMR spectrum (DMSO-d_6_, 151 MHz, 294 K) of (+)-Orthosporin.

Spectrum S. 9: ^1^H-NMR spectrum (DMSO-d_6_, 600 MHz, 294 K) of (+)-Citreoisocoumarin.

Spectrum S. 10: ^13^C{^1^H}-NMR spectrum (DMSO-d_6_, 151 MHz, 294 K) of (+)-Citreoisocoumarin.

Spectrum S. 11: ^1^H-NMR spectrum (DMSO-d_6_, 600 MHz, 294 K) of nor-Toralactone.

Spectrum S. 12: ^13^C{^1^H}-NMR spectrum (DMSO-d_6_, 151 MHz, 294 K) of nor-Toralactone.

Spectrum S. 13: ^1^H-NMR spectrum (DMF-d_7_, 600 MHz, 294 K) of Saintopin.

Spectrum S. 14: ^13^C{^1^H}-NMR spectrum (DMF-d_7_, 151 MHz, 294 K) of Saintopin.

Spectrum S. 15: ^1^H-NMR spectrum (CDCl_3_, 600 MHz, 294 K) of saintopin pentaacetate.

Spectrum S. 16: ^13^C{^1^H}-NMR spectrum (CDCl_3_, 151 MHz, 294 K) of saintopin pentaacetate.

# References

1. Edgar RC. MUSCLE: a multiple sequence alignment method with reduced time and space complexity. BMC Bioinformatics. 2004;5:113. doi:10.1186/1471-2105-5-113.

2. Trifinopoulos J, Nguyen L-T, Haeseler A von, Minh BQ. W-IQ-TREE: a fast online phylogenetic tool for maximum likelihood analysis. Nucleic Acids Res. 2016;44:W232-5. doi:10.1093/nar/gkw256.

3. Letunic I, Bork P. Interactive Tree of Life (iTOL) v6: recent updates to the phylogenetic tree display and annotation tool. Nucleic Acids Res. 2024;52:W78-W82. doi:10.1093/nar/gkae268.

4. Liu M, Ohashi M, Hung Y-S, Scherlach K, Watanabe K, Hertweck C, Tang Y. AoiQ Catalyzes Geminal Dichlorination of 1,3-Diketone Natural Products. J Am Chem Soc. 2021;143:7267–71. doi:10.1021/jacs.1c02868.

5. Geib E, Baldeweg F, Doerfer M, Nett M, Brock M. Cross-Chemistry Leads to Product Diversity from Atromentin Synthetases in Aspergilli from Section Nigri. Cell Chem Biol. 2019;26:223-234.e6. doi:10.1016/j.chembiol.2018.10.021.

6. Wieder C, Künzer M, Wiechert R, Seipp K, Andresen K, Stark P, et al. Biosynthesis of the Antifungal Polyhydroxy-Polyketide Acrophialocinol. Org. Lett. 2025. doi:10.1021/acs.orglett.4c04656.

7. van Bui M, Huynh BLC, Pham NKT, Nguyen TAT, Nguyen TTT, Nguyen KPP, Nguyen TP. Usneaceratins A and B, two new secondary metabolites from the lichen Usnea ceratina. Nat Prod Res. 2022;36:3945–50. doi:10.1080/14786419.2021.1901288.

8. Feng L-X, Zhang B-Y, Zhu H-J, Pan L, Cao F. Bioactive Metabolites from Talaromyces purpureogenus, an Endophytic Fungus from Panax notoginseng. Chem Nat Compd. 2020;56:974–6. doi:10.1007/s10600-020-03206-9.

9. Ishiuchi K, Nakazawa T, Ookuma T, Sugimoto S, Sato M, Tsunematsu Y, et al. Establishing a new methodology for genome mining and biosynthesis of polyketides and peptides through yeast molecular genetics. Chembiochem. 2012;13:846–54. doi:10.1002/cbic.201100798.

10. Hallock YF, Clardy J, Kenfield DS, Strobel G. De-O-methyldiaporthin, a phytotoxin from Drechslera siccans. Phytochemistry. 1988;27:3123–5. doi:10.1016/0031-9422(88)80012-8.

11. Watanabe A, Ono Y, Fujii I, Sankawa U, Mayorga ME, Timberlake WE, Ebizuka Y. Product identification of polyketide synthase coded by Aspergillus nidulans wA gene. Tetrahedron Letters. 1998;39:7733–6. doi:10.1016/S0040-4039(98)01685-2.

12. Newman AG, Vagstad AL, Belecki K, Scheerer JR, Townsend CA. Analysis of the cercosporin polyketide synthase CTB1 reveals a new fungal thioesterase function. Chem Commun (Camb). 2012;48:11772–4. doi:10.1039/c2cc36010a.
